# Atomic Precision CoCu Heterodimers with Pseudo‐D_3h_ Symmetry Enable Tandem Nitrate Reduction

**DOI:** 10.1002/advs.202523909

**Published:** 2025-12-22

**Authors:** Akash Prabhu Sundar Rajan, Jayaraman Theerthagiri, Piyapa Junmon, Wanwisa Limphirat, Nuttapon Yodsin, Myong Yong Choi

**Affiliations:** ^1^ Department of Chemistry (BK21 FOUR) Research Institute of Advanced Chemistry Gyeongsang National University Jinju Republic of Korea; ^2^ Department of Chemistry Faculty of Science Silpakorn University Nakhon Pathom Thailand; ^3^ Beamline Division Synchrotron Light Research Institute (SLRI) Nakhon Ratchasima Thailand; ^4^ Core‐Facility Center for Photochemistry & Nanomaterials Gyeongsang National University Jinju Republic of Korea

**Keywords:** CoCu heterodimer, pseudo‐D_3h_ symmetric structure, dual‐site tandem catalytic effect, pulsed laser techniques, langmuir‐hinshelwood‐type hydrogenation

## Abstract

The electrochemical reduction of nitrate (eNO_3_RR) to ammonia (NH_3_) is an efficient method for mitigating nitrate (NO_3_^−^) pollutant while offering sustainable NH_3_ generation under ambient environments. However, optimizing NO_3_^−^ adsorption on catalytic surfaces and promoting adsorbed hydrogen formation remain challenging. Herein, we introduce pulsed laser irradiation in liquid for the first time to design a metal–metal–ligand‐coordinated CoCu heterodimer catalyst with a pseudo‐D_3h_ symmetry anchored on nitrogen‐doped graphene oxide (CoCu‐HeD/NGO), enabling a tandem catalytic effect for the eNO_3_RR. The catalyst reaches a remarkable Faradaic efficiency of 91% at −0.4 V vs. RHE and a high NH_3_ production rate of 25 mg h^−1^ cm^−2^ at −0.5 V vs. RHE. Combined theoretical and in situ spectroelectrochemical analyses reveal that the synergistic interaction among Co and Cu dual sites enhances NO_3_^−^ adsorption, weakens N─O bonds, and facilitates the establishment of Langmuir–Hinshelwood‐type hydrogenation intermediates, steering the tandem reaction pathway toward selective NH_3_ formation. Furthermore, a Zn–nitrate battery with a CoCu‐HeD/NGO cathode integrates energy generation and NH_3_ synthesis with environmental remediation, delivering 5.26 mW cm^−2^ power density and stable discharge performance. Practical NH_3_ production is verified via Ar stripping–acid‐trapping methods. This work establishes a new paradigm for the rational design of site‐selective electrocatalysts for hybrid energy‐to‐chemical platforms.

## Graphical abstract

The Co─Cu heterodimer anchored on nitrogen‐doped graphene oxide substrate facilitates electrochemical nitrate reduction in alkaline medium. Co site promotes water dissociation to supply a proton, while Cu sites enhance nitrate adsorption and activation. The dimer‐metal centres allow the Langmuir–Hinshelwood (LH)‐type hydrogenation step in the nitrate reduction mechanism, wherein in situ generated hydrogen at the Co site reacts with adsorbed nitrate intermediates at the Cu site, driving selective ammonia production with concurrent OH^‒^ formation.

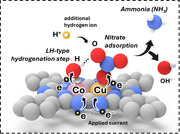

## 1 Introduction

As one of the foremost indispensable chemicals in modern society, the sustainable synthesis of ammonia (NH_3_) under ambient conditions is of paramount importance for global agricultural productivity, especially given the growing demand for decentralized fertilizer production and carbon‐neutral fuel technologies [1, 2, 3, 4]. Traditionally, large‐scale NH_3_ production relies on the Haber–Bosch method, which associates atmospheric N_2_ with H_2_ under high temperature and pressure, consuming over 1–2% of the global annual energy supply and emitting substantial quantities of CO_2_. Thus, the progress of alternative NH_3_ generation routes that are both energy‐effective and environmentally benign remains a critical challenge in catalysis and sustainable technology [3, 5]. Electrocatalytic NH_3_ synthesis from nitrogen‐containing sources (N_2_, nitric oxide (NO), nitrate (NO_3_^−^), and nitrite (NO_2_^−^)) has recently emerged as a compelling substitute to the Haber–Bosch strategy for green NH_3_ generation [2, 6]. Compared to direct N_2_ electroreduction, NO‐based reduction is more thermodynamically favorable due to the lesser dissociation energy of N═O (204 kJ mol^−1^) relative to N═N (945 kJ mol^−1^), and the aqueous solubility of NO‐based catalysts improves catalyst–reactant interactions [3, 7, 8, 9]. Importantly, wastewater from fertilizer plants, nuclear power stations, and electroplating industries contains nitrate‐rich effluents, providing abundant feedstock for waste‐to‐value NH_3_ conversion via electrochemical nitrate reduction (eNO_3_RR) [8, 10].

Despite these advantages, the practical implementation of eNO_3_RR remains hindered by the complexity of the multi‐electron and proton‐coupled reaction mechanism involving deoxygenation/hydrogenation from NO_3_^−^ to NH_3_ [11, 12, 13, 14]. This complexity often results in sluggish kinetics, poor product selectivity, and extensive competition from the HER, especially under alkaline conditions [15, 16, 17, 18, 19, 20]. Among various materials, Cu‐based materials are most studied for the eNO_3_RR because of their unique electronic structure, which improves the NO_3_^−^ adsorption for the initial reduction of NO_3_^−^ to NO_2_^−^ [21, 22, 23, 24]. However, Cu‐based samples exhibit limited capability for generating active hydrogen species for NO_2_^−^ hydrogenation and premature desorption of NO_2_ intermediates, resulting in suboptimal selectivity toward NH_3_ [25, 26, 27, 28]. By contrast, dual‐site catalysts have developed as a capable strategy to overcome scaling correlation limitations by enabling cooperative interactions between adjacent, chemically distinct metal centers [29, 30, 31]. Such synergistic interactions can break linear scaling constraints, modulate intermediate binding energies across different sites, and enhance proton–electron transfer dynamics. Recent studies have demonstrated that incorporating Co as a secondary catalytic site, with its strong affinity for water dissociation, can efficiently supply H* under alkaline conditions [32, 33]. This dual‐site catalyst can also enable the Langmuir–Hinshelwood (LH) mechanism, where H* generated on one metal center directly participates in the hydrogenation of *NO*_x_* species adsorbed on an adjacent site. The LH mechanism offers a thermodynamically and kinetically favorable pathway to overcome rate‐limiting steps in the eNO_3_RR in alkaline media [34, 35]. This spatial decoupling of proton and nitrate activation pathways promotes efficient H* spillover, markedly accelerating the kinetics and performance of the eNO_3_RR [13, 36, 37, 38, 39, 40, 41, 42]. Recent reports on Co─Cu catalysts are mainly based on bulk particles or dual‐atom systems. Bulk catalysts often rely on heterogeneous interfaces, which create non‐uniform active sites and limit control over tandem pathways. In other hand, dual‐atom systems improve atomic dispersion but generally lack direct metal–metal interactions, restricting efficient intramolecular H transfer. These drawbacks hinder precise mechanistic control and suppress the full potential of tandem catalysis [40, 43, 44, 45].

Herein, we designed CoCu heterodimer electrocatalytic sites anchored on nitrogen‐doped graphene oxide (CoCu‐HeD/NGO) via pulsed laser irradiation in liquid (PLIL). Co and Cu atoms exhibit a metal–metal–ligand (M–M–L_2_) coordination with two pyrrolic‐N ligands and one metal atom embedded in the NGO matrix, forming a well‐defined N_2_–Co–Cu–N_2_ dimer architecture with pseudo‐D_3h_ symmetry. In situ spectroelectrochemical analysis combined with density functional theory calculations reveals a bifunctional synergy: the Co site facilitates water dissociation, while the adjacent Cu site preferentially adsorbs *NO*_x_* intermediates. The spatial proximity of Co and Cu centers enables a LH‐type hydrogenation mechanism, wherein H* on Co undergoes direct intramolecular transfer to *NO*_x_* intermediates on Cu, accelerating the rate‐limiting deoxygenation and hydrogenation steps toward NH_3_ production. This tandem mechanism effectively decouples the proton–electron transfer pathway and considerably suppresses the HER, thereby improving both the selectivity and activity of the catalyst toward NH_3_. Consequently, the CoCu‐HeD/NGO catalyst demonstrates high NH_3_ activity (yield rate = 19.40 mg h^−1^ cm^−2^) and selectivity (Faradaic efficiency, FE = 96%) for the electrosynthesis of NH_3_ in an alkaline catholyte boosted by an acidic anolyte. In addition, the Co─Cu heterodimer structure weakens N─O bonds in NO_3_^‒^ ions, thereby lowering the energy barriers for NH_3_ production. Inspired by the exceptional site‐specific eNO_3_RR activity of CoCu‐HeD/NGO, a Zn–nitrate battery was fabricated using CoCu‐HeD/NGO as the cathode, delivering a high power density of 5.26 mW cm^−2^ and sustained NH_3_ generation over 10 h with ultrastable FE. To validate practical NH_3_ production, a combination of Ar stripping and acid trapping was employed for reliable NH_3_ quantification. These results offer valuable insights into the rational design of heteronuclear CoCu dual‐catalytic sites, presenting a promising strategy for developing efficient eNO_3_RR catalysts.

## 2 Results and Discussion

### 2.1 Formation of Heterodimer Catalysts and Structural Analysis

**FIGURE 1:**
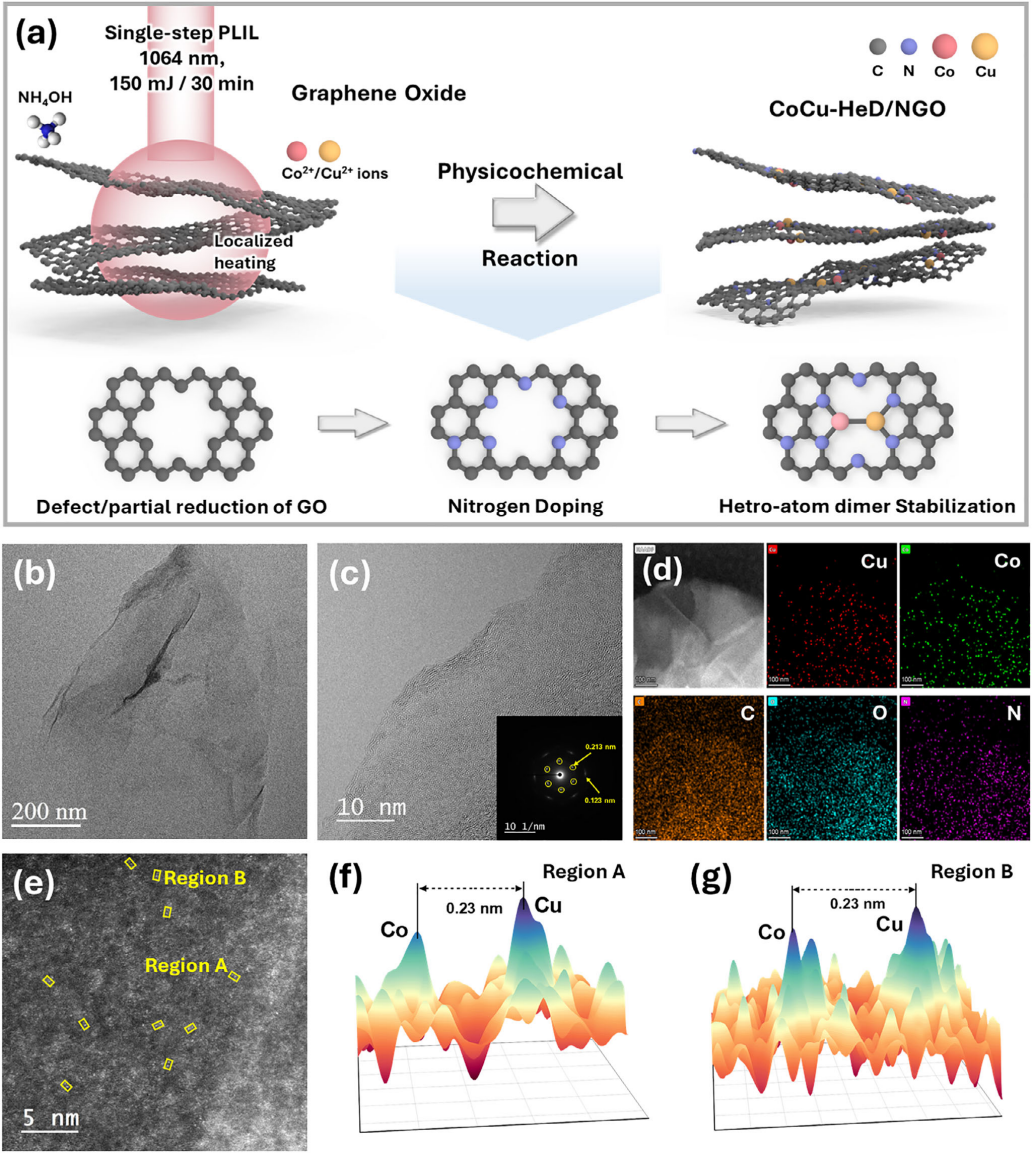
(a) Schematic design for the synthesis of CoCu‐HeD/NGO via PLIL; (b and c) low‐ and high‐magnification HRTEM images with SAED pattern shown in inset (c); (d) HAADF‐STEM image with EDS elemental mapping of CoCu‐HD/NGO; (e) HAADF‐STEM image of CoCu‐HeD/NGO showing bright spots corresponding to hetero‐single‐atom dimers (marked by yellow circles); (f and g) corresponding 3D‐contour plots of CoCu‐HeD/NGO highlighting dual‐atom interactions.

Conventional methods for synthesizing single‐atom or dimeric catalysts, such as high‐temperature pyrolysis or freeze‐drying, are typically time‐consuming and energy‐intensive. By contrast, PLIL offers several advantages, including ambient‐condition processing, rapid synthesis, absence of byproducts, surfactant‐free operation, and precise control over atomic dispersion and coordination environments, making it a highly efficient and scalable alternative for advanced catalyst fabrication [46, 47]. Herein, an atomically dispersed heterometallic CoCu dimer catalyst immobilized on NGO was synthesized via a single‐step PLIL‐assisted dual‐atom doping and anchoring strategy (Figure 1a). This approach simultaneously enables three critical physicochemical processes: (i) graphene oxide (GO) reduction, (ii) nitrogen doping, and (iii) atomic‐scale dispersion and stabilization of metal species. The detailed experimental procedure is presented in the Supporting Information. Under the PLIL process, localized photothermal effects induce defect formation and oxygen depletion within the GO matrix, facilitating structural reconfiguration to partially reduced GO [48, 49]. Consistent with theoretical predictions and prior studies, the laser‐driven decomposition of NH_3_ precursors generates reactive N* and NH** radical intermediates, which selectively incorporate into defect‐rich carbon sites within GO [50, 51]. FESEM and EDS mapping of GO and NGO (Figure S1) reveal a substantial increase in nitrogen content from 2.03 to 12.37 wt.%, underscoring the efficiency of nitrogen incorporation in GO. XPS survey spectra of GO and NGO exhibit a concurrent attenuation of oxygen signals, indicating partial deoxygenation of GO alongside N‐doping during the PLIL process (Figure S2a). High‐resolution C 1*s* XPS spectra reveal a substantial reduction in C═O functionalities and the formation of carbon vacancies, which are crucial defect sites for N doping (Figure S2b,c) [52]. Notably, the use of an aqueous/organic solvent mixture (1:1 *v*/*v* ratio of DI water:EtOH) during the synthesis of materials dissipates excessive localized heating, lowering potential material degradation [53]. High‐resolution N 1*s* XPS spectra (Figure S2d) further resolve distinct nitrogen configurations, including pyridinic, pyrrolic, and graphitic species, unambiguously confirming selective functionalization of defect sites in GO with nitrogen moieties [54]. Upon simultaneous introduction of cobalt (Co) and copper (Cu) precursors during PLIL, CoCu heterodimers were successfully anchored onto the N‐sites of GO (NGO), forming CoCu‐HeD/NGO. In addition to CoCu‐HeD/NGO, Cu─Cu and Co─Co homodimers were synthesized on the NGO substrate using a similar synthetic strategy, referred to as Cu_2_‐HoD/NGO and Co_2_‐HoD/NGO, respectively. Despite the reductive environment, PLIL enables the formation of dimers through ultrafast reaction kinetics, spatial confinement, and strong interactions between metal ions (Co^2+^ and Cu^2+^) and the NGO support. High‐energy laser pulses generate localized photothermal and photochemical effects, rapidly reducing metal ions within nanoseconds and limiting atomic diffusion and aggregation. Concurrently, NGO offers abundant coordination sites, particularly pyrrolic and graphitic nitrogen, which serve as thermodynamically favorable anchors for the immobilization and stabilization of isolated metal atoms or dimers, preventing nanoparticle formation. Additionally, the confined reaction environment and low precursor concentrations further suppress particle growth, collectively enabling the formation of atomically dispersed catalytic sites. As shown in Figure S3, EDS analysis determined Co and Cu loadings in the CoCu heterodimer to be 1.47 and 1.82 wt.%, respectively. These values closely match those obtained from ICP‐OES, which measured Co and Cu loadings of ∼1.54 and ∼1.65 wt.%, correspondingly (Table S1).

Figure 1b,c presents the high‐ and low‐magnification HRTEM images of the CoCu‐HeD/NGO, respectively. Notably, no nanoparticles or clusters are observed on the GO lattice, suggesting atomic‐level doping within the NGO lattice. Furthermore, the SAED pattern of CoCu‐HeD/NGO (inset in Figure 1c) reveals a hexagonal diffraction pattern with well‐defined diffraction spots, characteristic of the graphene lattice. The diffraction rings resemble the (100) and (010) planes of graphene, with an in‐plane atomic distance (lattice parameter) of 0.213 nm [55]. Elemental mapping analysis (Figure 1d) using HAADF‐STEM–EDS, further confirms the homogeneous distribution of heteroatoms Co and Cu within the CoCu‐HeD/NGO catalyst. The aberration‐corrected HAADF‐STEM image (Figure 1e) reveals atomic‐sized bright dots corresponding to Co and Cu species on the CoCu‐HeD/NGO surface, confirming their atomic dispersion on NGO. Upon closer examination, diatomic Co─Cu pairs, predominantly in the form of dimers, are frequently observed on NGO, highlighting the tendency of these atoms to exist as paired species. 3D Contour plots corresponding to regions A and B in Figure 1e, presented in Figure 1f,g, further confirm a Co─Cu bond length of 0.23 nm, indicating dimer formation. The bright spot in the contour plots of CoCu‐HeD/NGO is identified as Cu owing to Z‐contrast between the heavier Cu and the lighter Co atom.

Additional histogram analyses at different spots of CoCu‐HeD/NGO (Figure S4) clearly confirm the presence of dimer sites on NGO. The X‐ray diffraction (XRD) patterns of GO, NGO, Cu_2_‐HoD/NGO, Co_2_‐HoD/NGO, and CoCu‐HeD/NGO (Figure S5) show only a single diffraction peak at 11.94°, corresponding to the stacking of GO layers [56]. After PLIL treatment, this diffraction peak broadens owing to defect formation in the GO layers. Notably, no secondary impurity peaks are observed, suggesting high phase purity of NGO. Combined with Raman spectroscopy (Figure S6), these results confirm the successful fabrication of CoCu heterodimer structures on the GO lattice without the production of metal clusters or nanoparticles. For systematic analyses, the detailed morphologic and compositional characterizations of the fabricated Cu_2_‐HoD/NGO and Co_2_‐HoD/NGO are exposed in Figures S5–S9.

### 2.2 Electronic State and Coordination Analyses

XPS was performed to understand the chemical and electronic states of Cu_2_‐HoD/NGO, Co_2_‐HoD/NGO, and CoCu‐HD/NGO. The XPS survey spectra (Figure S10a) clearly reveal the occurrence of C, O, and N in all samples. Additionally, Cu is detected in Cu_2_‐HoD/NGO, Co in Co_2_‐HoD/NGO, and both Cu and Co in CoCu‐HeD/NGO, consistent with EDS results. The C 1*s* core‐level spectra of Cu_2_‐HoD/NGO, Co_2_‐HoD/NGO, and CoCu‐HeD/NGO (Figure S10b) resembles that of NGO, featuring *sp* ^2^ C═C, C─N/C─O, C═O, COOH, and π–π* species at 284.6, 256.6, 286.7, 288.1, and 289.3 eV, correspondingly. The O 1*s* spectrum (Figure S10c) shows no M─OH and M─O species, suggesting the absence of metal–oxygen interactions in Cu_2_‐HoD/NGO, Co_2_‐HoD/NGO, and CoCu‐HeD/NGO. Contrarily, the N 1*s* core‐level spectra of Cu_2_‐HoD/NGO, Co_2_‐HoD/NGO, and CoCu‐HeD/NGO (Figure S10d) confirms the existence of pyridinic N (398.2 eV), pyrrolic N (400.0 eV), graphitic N (401.7 eV), and oxidized N (403.2 eV), along with a distinct peak at 399.2 eV, indicative of metal–nitrogen (M─N) coordination [38, 52]. Notably, the increase in pyrrolic N and M─N peak at 399.2 eV in the homo‐ and heterodimer samples suggest the probable formation of dimer structures with a porphyrin‐like configuration. Co and Cu 2*p* spectra of Co_2_‐HoD/NGO, Cu_2_‐HoD/NGO, and CoCu‐HeD/NGO display characteristic 2*p*_3/2_ and 2*p*_1/2_ peaks, suggesting the positive oxidation states of Co and Cu species (Figure S11b,c). Compared to Co_2_‐HoD/NGO, the Co 2*p* binding energy (B. E) in CoCu‐HeD/NGO shifts to lower values (Figure S11b); similarly, the Cu 2*p* also shifts to lower B.Es in CoCu‐HeD/NGO compared with Cu_2_‐HoD/NGO (Figure S11c). These energy shifts in the Co and Cu B.Es strongly suggest electronic interactions between Co and Cu at the atomic interface, likely owing to direct Co─Cu bond formation at the atomic scale. Bader charge analysis further elucidates the XPS results and provides quantitative insight into the electronic interactions within CoCu‐HeD/NGO. As shown in Figure S12, the Co and Cu atoms exhibit Bader charges of +0.41 |e| and +0.61 |e|, respectively, indicating partial electron depletion on both metal centers. The net positive charge of +1.02 |e| on the CoCu dimer is balanced by an equivalent negative charge (–1.02 |e|) localized on the NGO substrate, revealing a significant interfacial charge transfer from the support to the bimetallic site. Moreover, the relatively lower positive charge on Co compared to Cu demonstrates electron migration from Cu to Co. The lower binding energies observed for Co 2*p* and Cu 2*p* in CoCu‐Hed/NGO relative to Co_2_‐HeD/NGO and Cu_2_‐HeD/NGO can thus be rationalized by considering the cooperative interaction of both metal sites with the NGO support, which modifies the overall electronic environment of the Co–Cu ensemble. These findings collectively confirm that the synergistic charge redistribution from NGO to the CoCu dimer and between the Co and Cu atoms, which is expected to modulate the local electronic environment and facilitate the activation of adsorbed intermediates during the electrocatalytic process.

**FIGURE 2:**
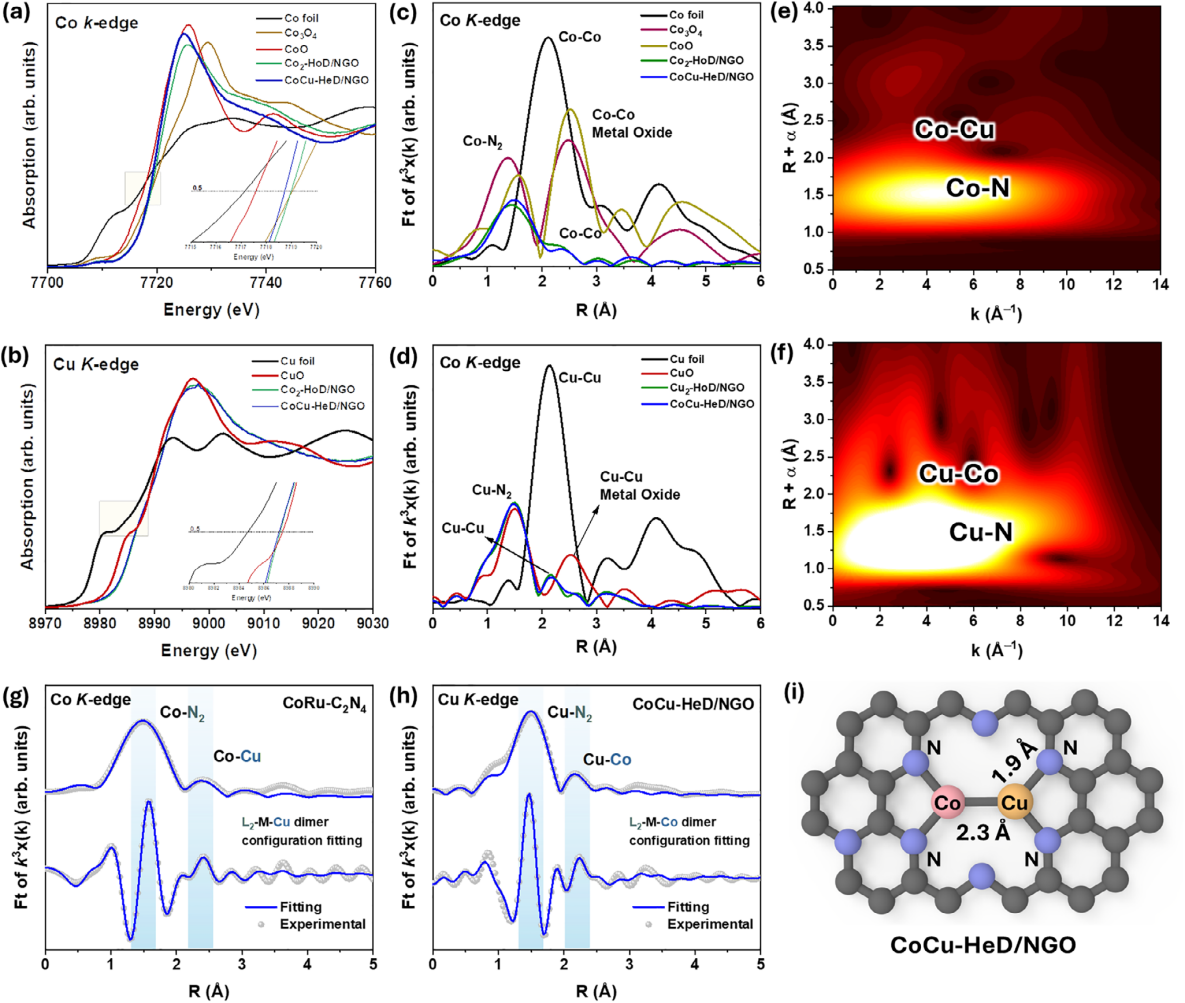
XANES spectra of (a) Co *k*‐edge and (b) Cu *k*‐edge; FT‐EXAFS spectra in R‐space of Cu_2_‐HoD/NGO, Co_2_‐HoD/NGO, and CoCu‐HeD/NGO at (c) Co and (d) Cu *k*‐edges; Wavelet‐transformed k^2^‐weighted EXAFS contour spectra of CoCu‐HeD/NGO at (e) Co and (f) Cu *k*‐edges; Fitting results of the EXAFS spectra of CoCu‐HeD/NGO at (g) Co and (h) Cu *k*‐edges in R space of Cu *k*‐edge; and (i) Illustrating the pseudo D_3h_ structure of CoCu‐HeD/NGO.

The chemical features and coordination environment were analyzed using XANES and EXAFS (These measurements were performed at the Core‐Facility Center for Photochemistry & Nanomaterials, Gyeongsang National University, using an in situ cryo X‐ray absorption spectrometer, IC‐XAS (NFEC‐2025‐07‐307201)), as shown in Figure 2a–d. The Co *k*‐edge XANES spectra of Co_2_‐HoD/NGO and CoCu‐HeD/NGO exhibit absorption edge energies between those of metallic Co^0^ and cobalt oxides (CoO and Co_3_O_4_), representing a valence state higher than Co^0^ (Figure 2a). Similarly, the Cu *k*‐edge spectra reveal that the Cu oxidation state in Cu_2_‐HoD/NGO and CoCu‐HeD/NGO surpasses metallic Cu^0^, as evidenced by higher edge energies than Cu foil and CuO (Figure 2b). As shown in Figure S13a, a 1*s* to 4*p_z_* shakedown transition characteristic of square‐planar coordination with pseudo‐D_3h_ local symmetry at 7709.9 eV appears in both the Co *k*‐edge XANES spectrum of Co_2_‐HoD/NGO and CoCu‐HeD/NGO [57]. These weak pre‐edge peaks imply the formation of a pseudo‐D_3h_ symmetric system, with each Co atom coordinated to two pyrrolic‐N atoms and one metal atom (N_2_─Co─Co─N_2_) [58]. Compared to the homodimer, the heterodimer exhibits a profound reduction in pre‐edge peak intensity, suggesting Cu incorporation into *D*_3h_ symmetric structure alongside Co. The CoCu heterodimer environment was modeled as a Cu─Co─N_2_ structure, where each metal atom is coordinated and stabilized by two pyrrolic‐N atoms. Similar to the Co pre‐edge, the Cu pre‐edge of both homo‐ and hetero‐dimer samples also exhibits a pre‐edge peak (1*s* to 4*p_z_* shakedown transition), indicating formation of a pseudo‐D_3h_ symmetry along with pyrrolic‐N molecules and Co atoms in CoCu‐HeD/NGO (Figure S13b) [59]. However, owing to Jahn–Teller distortions of the Cu atom, the intensity of the ideal pseudo‐D_3h_ symmetry peak is lower compared to the Co pre‐edge peak [60]. The Co white line absorption peak in the near‐edge position for Co_2_‐HoD/NGO and CoCu‐HeD/NGO is near to the Co_3_O_4_ reference, suggesting that Co atoms in the catalyst possess a positively charged valence state (Figure 2a). The precise oxidation states of Co in Co_2_‐HoD/NGO and CoCu‐HeD/NGO were determined as +2.58 and +2.22, respectively, by analyzing the XANES energy at the half‐edge jump (Figure S14a). Meanwhile, the oxidation state of Cu determined via XANES profiles for Cu_2_‐HoD/NGO and CoCu‐HeD/NGO are +1.86 and +1.79, respectively (Figure S14b). These oxidation states of Co and Cu align well with Co 2*p* and Cu 2*p* XPS spectra. The distinct oxidation states of Co and Cu suggest partial electron sharing between Co and Cu via ionic interactions, indicating strong electronic coupling between Co and Cu metal centers. This electronic interplay leads to the redox flexibility of Co and Cu, enabling efficient charge transfer at the catalyst‐electrolyte interface during eNO_3_RR and making CoCu‐HeD/NGO an effective electrochemical catalyst.

Figure 2c,d present the FT, k^2^‐weighted EXAFS spectra at the Co and Cu *k*‐edge for CoCu‐HeD/NGO, along with Co_2_‐HoD/NGO and Cu_2_‐HoD/NGO homodimer samples to analyze their local coordination environments [38, 40, 58, 61]. In the Co *k*‐edge FT‐EXAFS spectra (Figure 2c), major peaks in the R‐space appear at 1.44 and 1.50 Å for Co_2_‐HoD/NGO and CoCu‐HeD/NGO, assigned to first‐shell scattering of the Co─N coordination. The observed difference in average Co─N bond length between Co_2_‐HoD/NGO and CoCu‐HeD/NGO further indicates a distorted pseudo‐D_3h_ symmetry in CoCu‐HeD/NGO. Furthermore, second‐shell scattering peaks at 2.30 and 2.40 Å confirm Co─Co and Co─Cu bonding, revealing M─M coordination in Co_2_‐HoD/NGO and CoCu‐HeD/NGO. In contrast to Co FT‐EXFAS, the Cu *k*‐edge FT‐EXAFS (Figure 2d) shows first‐shell scattering at 1.50 and 1.47 Å for Cu_2_‐HoD/NGO and CoCu‐HeD/NGO, respectively, confirming the asymmetric or distorted nature of the CoCu heterodimer. Second‐shell scattering peaks at 2.15 Å (Cu_2_‐HoD/NGO) and 2.16 Å (CoCu‐HeD/NGO) confirm the formation of Cu─M dimer. Comparing Co and Cu *k*‐edge EXAFS spectra, the difference in bond lengths between homo‐ and hetero‐dimers reveals that reduced electron density around the Co site weakens its orbital overlap with the N atom, while the redistributed electrons at the Cu site enhance orbital hybridization between Co and Cu in CoCu‐HeD/NGO. This Co─Cu orbital hybridization shifts the metal *d*‐band center of CoCu‐HeD/NGO, thereby tuning the active site for effective NO_3_^−^ reduction to NH_3_. The FT‐EXAFS results correlate well with wavelet‐transformed EXAFS contour spectra at Co and Cu *k*‐edges (Figure 2e,f and Figure S15).

Furthermore, the structural information and coordination numbers (CNs) of CoCu‐HeD/NGO, along with Co_2_‐HoD/NGO and Cu_2_‐HoD/NGO, were analyzed via least‐square EXAFS fitting of R‐space oscillations. The EXAFS results with M–M–L2 configuration for CoCu‐HeD/NGO, Co_2_‐HoD/NGO, and Cu_2_‐HoD/NGO are shown in Figure 2g,h and Figure S16, with fitting parameters listed in Table S2. The simulated curves fit well with the experimental spectra. The first scattering shell of Co─N and Cu─N in CoCu‐HeD/NGO was well fitted with CNs of 2.05 and 2.21, respectively. Meanwhile, the second scattering shell of Co–Cu/Cu─Co paths fitted CNs of 1.22 and 1.32. Similarly, EXAFS spectra were modeled using Co─N_4_ and Cu─N_4_ coordination for comparison. Results indicate that exclusive coordination of Co and Cu with four nitrogen atoms yields poorer FT curve fitting than the M–M–N_2_ configuration, as shown in Figure S17. These fitting results strongly imply that Co and Cu in CoCu‐HeD/NGO, Co_2_‐HoD/NGO, and Cu_2_‐HoD/NGO adopt an M–M–L_2_ coordination environment with pseudo‐D_3h_ symmetry, involving two nitrogen atoms and one neighboring metal atom (Figure 2i and Figure S18). This M–M–L_2_ configuration closely aligns with structural models from STEM and XPS analyses, rather than conventional dual‐atom configurations such as Co–N_4_ or Cu–N_4_. Consequently, the successful formation of Co–Cu dimer sites on the NGO substrate is expected to synergistically increase the eNO_3_RR performance of CoCu‐HeD/NGO.

### 2.3 Electrocatalytic eNO_3_RR Performance

Driven by first‐principle predictions and the unique coordinated CoCu dimer structure, we performed eNO_3_RR experiments in a three‐electrode H‐cell setup for all prepared samples (NGO, Cu_2_‐HoD/NGO, Co_2_‐HoD/NGO, and CoCu‐HeD/NGO). The major liquid product, NH_3_, formed during the eNO_3_RR was measured and estimated by the indophenol blue process [62, 63]. Ultraviolet–visible (UV–vis) calibration curves for NH_3_ determination using NH_4_Cl solutions of known concentrations as standards via the indophenol blue method are depicted in Figure S19. To elucidate the role of proton (H^+^) during the eNO_3_RR, we first explored the eNO_3_RR performance of the CoCu‐HeD/NGO catalyst under alkaline and acidic anolyte conditions. In all cases, the cathodic compartment contained 0.1 KNO_3_ in 1.0 M KOH, separated from the anolyte by a Nafion membrane. As revealed in Figure S20a, the LSV curve recorded with the acidic anolyte exhibits a slightly higher onset potential than in the alkaline anolyte. However, at highly negative potentials, current density increases faster under acidic conditions and remains higher over the entire potential range compared to alkaline conditions. Notably, the LSV curve in the alkaline anolyte exhibits a low current density, likely owing to mass transport limitations from insufficient proton availability during the hydrogenation steps of the eNO_3_RR [64]. In addition to the LSV results, the slower reaction kinetics under alkaline anolyte conditions is further evidenced by Tafel slope investigation (Figure S19b). The NH_3_ yield rate (YR) and FE were evaluated after 1 h of eNO_3_RR electrolysis at diverse applied potentials (Figure S21a,b). The CoCu‐HeD/NGO catalyst operating with an acidic anolyte exhibited an impressive NH_3_ YR of 19.40 mg h^−1^ cm^−2^ with an FE of 96% and a current density (*j*) of 115 mA cm^−2^. By contrast, under alkaline anolyte, the YR and FE decreased to 12.56 mg h^−1^ cm^−2^ and 78%, correspondingly (Figure S22a,b). These results clearly indicate that the eNO_3_RR is more favorable with acidic anolyte, where the enhanced availability and H^+^ transport from the anode to the cathode accelerate hydrogenation of nitrogen‐containing intermediates. In comparison, alkaline anolyte, where H^+^ is generated solely through sluggish water dissociation, limits proton availability and thus hampers efficient NH_3_ production [65].

**FIGURE 3:**
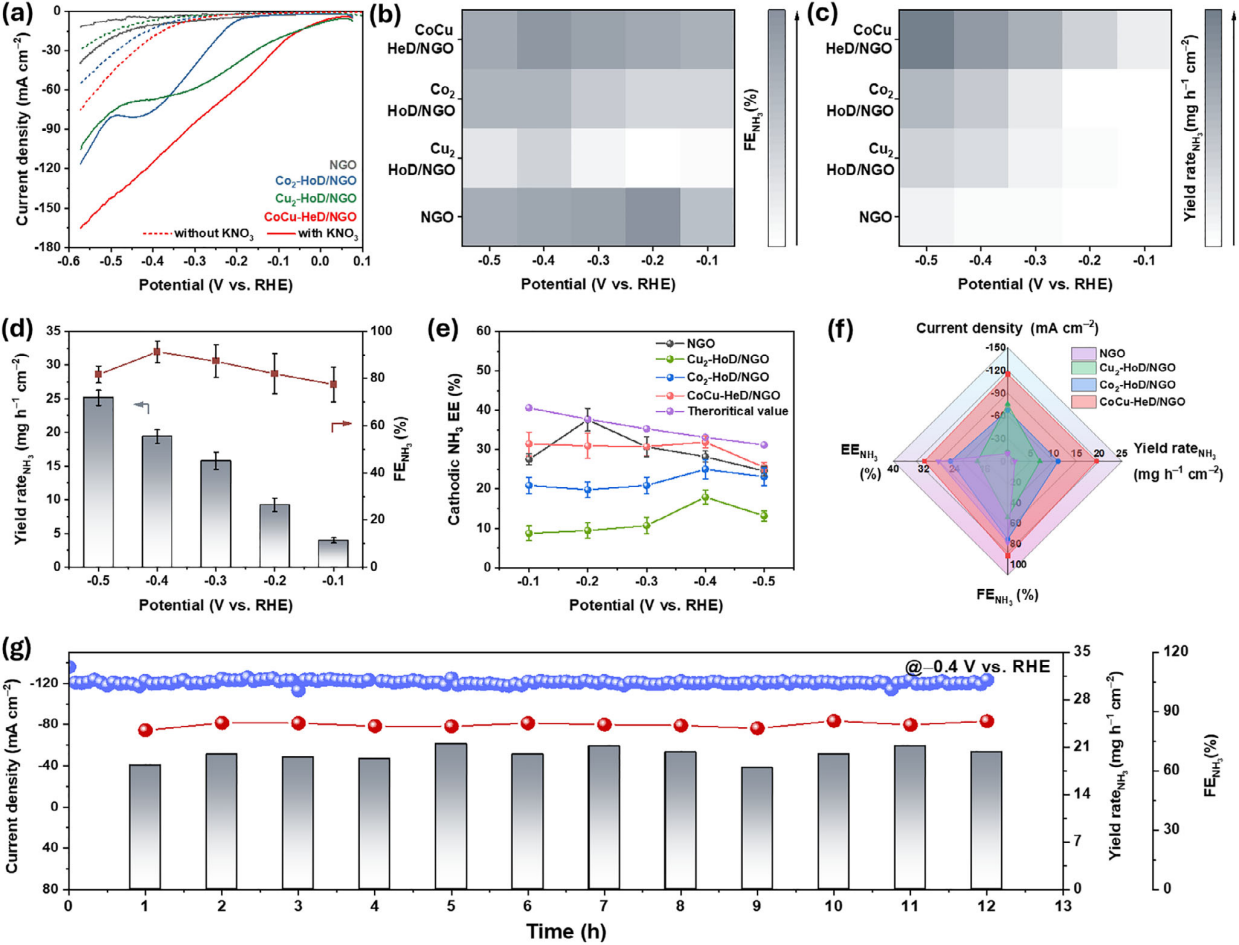
Electrocatalytic eNO_3_RR performance: (a) LSV in 1.0 M KOH electrolyte with (solid line) and without (dotted line) 0.1 M KNO_3_ for NGO, Co_2_‐HoD/NGO, Cu_2_‐HoD/NGO, and CoCu‐HeD/NGO; heatmaps of (b) NH_3_ FE(%), (c) NH_3_ YR (mg h^−1^ cm^−2^) produced over NGO, Co_2_‐HoD/NGO, Cu_2_‐HoD/NGO, and CoCu‐HeD/NGO at various potentials; (d) potential‐dependent NH_3_ yield rate and FE over CoCu‐HD/NGO; (e) cathodic energy efficiency (EE) for NH_3_ on NGO, Co_2_‐HoD/NGO, Cu_2_‐HoD/NGO, and CoCu‐HeD/NGO; (f) comparative electrocatalytic eNO_3_RR performance of CoCu‐HD/NGO; and (g) time‐dependent cyclic stability test of CoCu‐HeD/NGO with catholyte replaced every 1 h for 12 cycles at −0.4 V vs. RHE (error bars signify standard deviations from three independent studies).

Furthermore, the electrocatalytic eNO_3_RR activity of all samples was assessed in an H‐cell configuration using a 0.5 M H_2_SO_4_ acid anolyte and an alkaline catholyte comprising 1.0 M KOH and 0.1 M KNO_3_. As shown in Figure 3a, the LSV curve for CoCu‐HeD/NGO shows a markedly enhanced current density at increasingly negative potentials in the presence of NO_3_^−^. As expected, CoCu‐HeD/NGO exhibits higher current density than Cu_2_‐HoD/NGO and Co_2_‐HoD/NGO. The NH_3_ YR and FE were determined after 1 h of eNO_3_RR electrolysis (Figure S23) at diverse potentials from −0.1 to −0.5 V vs. RHE using UV–vis spectroscopy (Figure S24). CoCu‐HeD/NGO achieves the highest NH_3_ FE and yield rate across all tested potentials, indicating its superior tandem catalytic activity for the eNO_3_RR (Figure 3b,c). The FE of CoCu‐HeD/NGO exhibits a volcano‐type dependence on applied potential, reaching 77% at −0.1 V vs. RHE and peaking at 96% at −0.4 vs. RHE, where the corresponding *j* reached 115 mA cm^−2^. FE slightly decreased to 81% at −0.5 V vs. RHE (Figure 3d). Although NGO shows a slightly higher FE at −0.2 V, the corresponding yield rate at this potential is negligible, indicating the superior overall activity of CoCu‐HeD/NGO. At the maximum FE, CoCu‐HeD/NGO delivers an NH_3_ YR of ∼19.40 mg h^−1^ cm^−2^ at −0.4 V vs. RHE, which is 2.76 and 1.76 times higher than those of Cu_2_‐HoD/NGO (7.04 mg h^−1^ cm^−2^) and Co_2_‐HoD/NGO (11.03 mg h^−1^ cm^−2^), respectively. A peak NH_3_ half‐cell energy efficiency (EE) of 31.90% ± 1.5% was achieved with CoCu‐HeD/NGO, demonstrating superior energy utilization compared to the other catalysts (Figure 3e). To confirm NH_3_ originated from the eNO_3_RR using CoCu‐HeD/NGO, isotopic labeling experiments using ^1^H NMR spectroscopy were conducted in 1.0 M KOH/0.1 M ^14^NO_3_^−^ (Figure S25: calibration curve derived from peak integral areas). Typical triplet peaks corresponding to ^14^NH_4_^+^ appeared in the H^1^ NMR spectra after 1 h of eNO_3_RR electrolysis using ^14^NO_3_^−^ [66]. Increasing peak intensity with applied potential further confirmed NH_4_^+^ originated from the eNO_3_RR, not contamination (Figure S26a). NH_3_ yield rate determined from NMR was 19.03 mg h^−1^ cm^−2^ at −0.4 V vs. RHE, consistent with the UV–vis results, validating the reliability of NH_3_ production (Figure S26b). Overall, CoCu‐HeD/NGO outperforms homodimer catalysts on all metrics for the eNO_3_RR, as shown in Figure 3f. Remarkably, it exhibited exceptional long‐span stability, sustaining a *j* of about −120 mA cm^−2^ over a 10 h cyclic with stable NH_3_ YR (∼19.94 mg h^−1^ cm^−2^) and FE (∼84%) (Figure 3g). Post‐electrolysis characterization, including XPS (Figure S27), confirms the retention of elemental composition and electronic structure of CoCu‐HeD/NGO, indicating its outstanding structural and chemical stability toward the eNO_3_RR.

### 2.4 Understanding the Tandem Catalytic Effect on CoCu‐HeD/NGO

**FIGURE 4:**
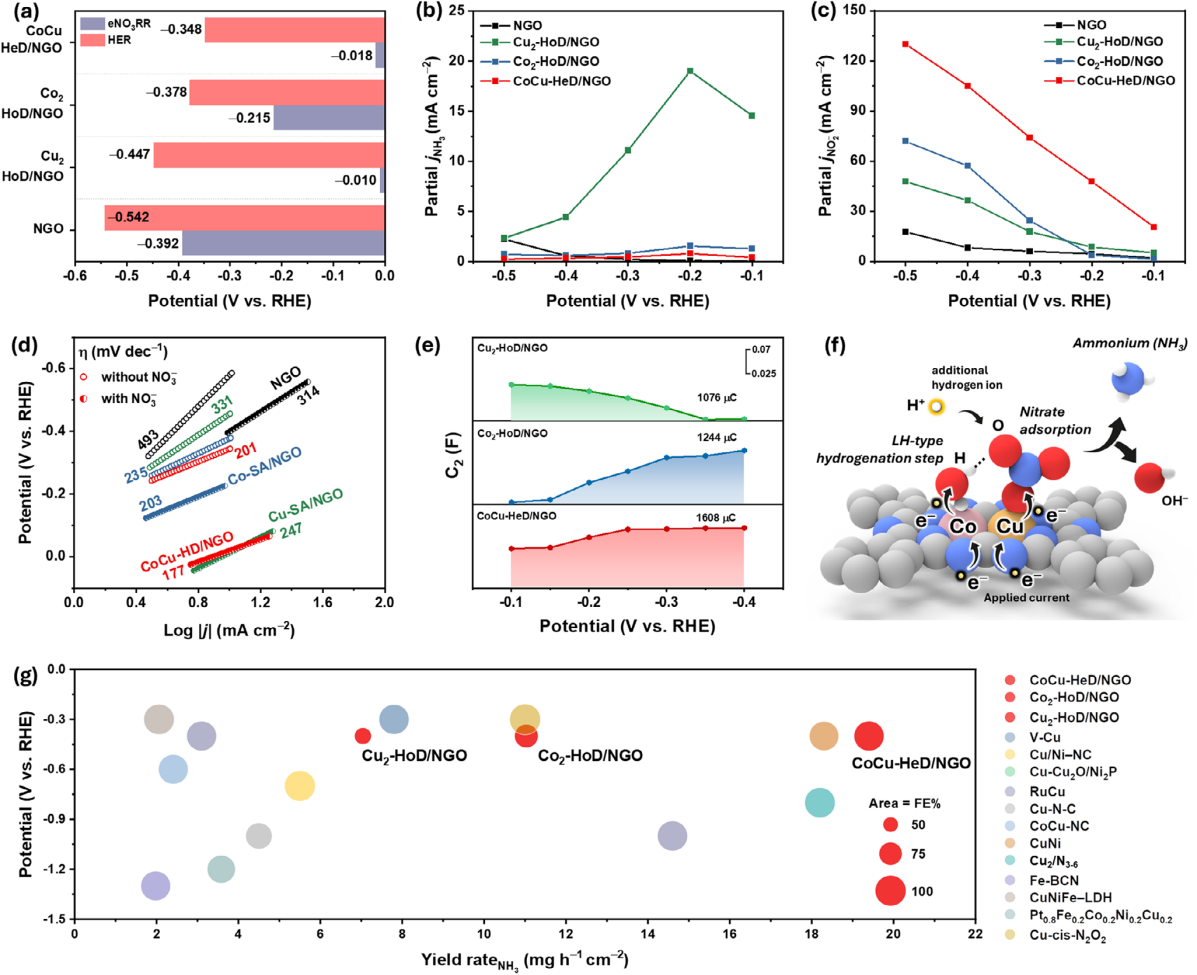
(a) Potential derived from LSV at −10 mA cm^−2^ for different catalysts for both HER and eNO_3_RR; partial *j* of (b) NH_3_ and (c) NO_2_^−^ for all catalysts at different potentials; (d) Tafel plots of NGO, Co_2_‐HoD/NGO, Cu_2_‐HoD/NGO, and CoCu‐HeD/NGO in 1.0 M KOH with and without 0.1 M KNO_3_; (e) integrated plot of C_2_ vs. V of Co_2_‐HoD/NGO, Cu_2_‐HoD/NGO, and CoCu‐HeD/NGO in 1.0 M KOH; (f) Illustration of nitrate reduction to ammonia on a CoRu dimer catalyst, highlighting NO_3_^−^ adsorption, electron transfer, and LH‐type hydrogenation steps; (g) comparison of eNO_3_RR performance of CoCu‐HeD/NGO with other catalysts reported in the literature (circle area indicates FE).

To understand the superior NH_3_ yield rate and FE across all applied potentials, it is essential to explicate the underlying reaction mechanism of the eNO_3_RR (NO_3_^−^ to NH_3_). The reaction generally proceeds via the deoxygenation of NO_3_^−^ to intermediate nitrogen species, followed by stepwise hydrogenation to form NH_3_ [67, 68]. During these stages, Cu homodimer exhibits a strong adsorption affinity toward NO_3_^−^ at lower onset potentials than Co homodimer and CoCu heterodimer, as indicated by the potential essential to reach −10 mA cm^−2^ (Figure 4a). At high applied potentials, in addition to NH_3_, NO_2_^−^ is formed as a major byproduct and key intermediate, identified and estimated using the Griess method via UV–vis spectroscopy (Figures S28 and S29) [69]. A comparative analysis of the FE and yield rates of NH_3_ and NO_2_^−^ for NGO, Cu_2_‐HoD/NGO, Co_2_‐HoD/NGO, and CoCu‐HeD/NGO is presented in Figures S30–S33. Cu_2_‐HoD/NGO exhibits substantially higher yield of prematurely desorbed NO_2_^−^ compared to the other catalysts, suggesting the inefficient conversion of intermediates to NH_3_. Remarkably, the synergistic interaction among Co and Cu in the CoCu heterodimer modulates the *d*‐band center of Cu, thereby reducing the desorption of the NO_2_^−^ intermediate [70]. This implies that Cu *d*‐center in Cu_2_‐HoD/NGO has a weaker affinity for key intermediates adsorption, such as NO_2_^−^ and NH*_x_* ^*^, lowering selectivity toward NH_3_, as detailed in the theoretical analysis. Partial current densities for NH_3_ (*j*_NH3_) and NO_2_^−^, calculated from their respective FEs, are shown in Figure 4b,c at diverse potentials. CoCu‐HeD/NGO delivers an outstanding *j*_NH3_ of 105.01 mA cm^−2^ at −0.4 V vs. RHE, highlighting its exceptional activity and selectivity toward complete NO_3_^−^ reduction to NH_3_.

Besides the desorption behavior of reaction intermediates, the performance of NO_3_^−^ reduction is also strongly influenced by the availability of optimally adsorbed hydrogen species (H*) on the catalyst surface, which are generated via water dissociation in alkaline media. The behavior of H* on different catalytic sites was initially investigated through cyclicvoltammetry (CV) in 1.0 M KOH [70]. As revealed in Figure S34a, a prominent signal at ∼0.3 V vs. RHE corresponds to hydrogen desorption, indicating abundant surface‐adsorbed H*. As expected, Cu_2_‐HoD/NGO exhibits negligible H* peak intensity, suggesting poor H* generation via water dissociation. The integrated H* peak area follows the following order: CoCu‐HeD/NGO > Co_2_‐HoD/NGO > Cu_2_‐HoD/NGO. Similarly, CV graphs recorded in the occurrence of NO_3_^−^ (Figure S34b) show consistent hydrogen desorption behavior at ∼0.3 V vs. RHE for all catalysts, highlighting the crucial role of Co sites in enhancing water dissociation and H* generation. These findings confirm H_2_O as the exclusive proton source under alkaline conditions and suggest the eNO_3_RR mechanism likely proceeds via a Langmuir–Hinshelwood (LH) pathway, where both H*‐ and NO_3_^−^‐derived intermediates co‐adsorb on the catalyst surface to facilitate proton‐coupled electron transfer [34]. Tafel slope analysis (Figure 4d) further reveals that the Co‐containing catalysts have faster kinetics for both the HER and eNO_3_RR. Notably, the CoCu heterodimer outperforms homodimeric counterparts, where the enhanced HER kinetics support the eNO_3_RR mechanism for efficient H* generation from water dissociation is essential for the protonation steps in NO_3_^−^ reduction. The moderate HER kinetics reflect an optimal H coverage that facilitates eNO_3_RR via the Langmuir–Hinshelwood pathway rather than competing with eNO_3_RR. To further probe H* adsorption behavior on the catalyst, EIS was conducted at different potentials (from −0.1 to −0.4 V vs. RHE) using 1.0 M KOH (catholyte) and 0.5 M H_2_SO_4_ (anolyte) (Figure S35). Nyquist plots were fitted using a dual‐parallel equivalent circuit model; extracted parameters for each circuit element are summarized in Figure S35 and Table S3 [71, 72]. The first parallel component (comprising a constant phase element, CPE; and charge transfer resistance, *R*_CT_) corresponds to the electron transfer kinetics of the eNO_3_RR process. The considerably low *R*_CT_ values across all the catalysts indicate efficient electron conductivity, enabling rapid charge transfer during the eNO_3_RR. The second parallel component accounts for the pseudocapacitance (*C*_2_) and resistance associated with H* adsorption (R_H*_) behavior, representing proton‐coupled electron transfer arising from water dissociation. Integration of *C*_2_ with respect to potential yields the hydrogen adsorption charge (*Q*_H_), which quantifies the H* adsorption capacity of the catalyst surface [73]. As shown in Figure 4e, CoCu‐HeD/NGO exhibits the highest *Q*_H_ value (1608 µC), surpassing that of Co_2_‐HoD/NGO (1244 µC) and Cu_2_‐HoD/NGO (1076 µC), highlighting the pivotal role of Co as the main active site for H* adsorption. Additionally, *C*_dl_, correlated with ECSA, was calculated using CV graphs in the non‐Faradaic window at different scan rates (Figure S36). *C*_dl_ values for NGO, Co_2_‐HoD/NGO, Cu_2_‐HoD/NGO, and CoCu‐HeD/NGO are 46.18, 77.37, 67.23, and 95.58 µF cm^−2^, respectively (Figure S37). The high *C*_dl_ of CoCu‐HeD/NGO indicates a large ECSA and a numerous exposed active site, accredited to the strong electronic and geometric interaction among Co and Cu within the heterodimer structure. Collectively as shows in the Figure 4f, these findings suggest that the CoCu heterodimer acts as a dual‐site catalyst following an LH‐type mechanism, where Cu preferentially adsorbs NO_3_^−^ and Co actively promotes H* adsorption, thereby enabling efficient and selective NH_3_ production during the eNO_3_RR. This structural feature enhances the overall eNO_3_RR catalytic performance of CoCu‐HeD/NGO, establishing it as one of the most effective catalysts for NH_3_ synthesis via the eNO_3_RR (Table S4 and Figure 4g).

### 2.5 Insights Into CoCu‐HeD/NGO Catalytic Active Sites for the eNO_3_RR via In Situ Raman and Ex Situ FTIR Spectroelectrochemistry

To provide solid experimental support for the DFT analysis, in situ Raman spectroelectrochemistry (Figure S38) and ex situ FTIR spectra (Figure S39) were collected for the CoCu‐HeD/NGO catalyst during the eNO_3_RR at various potentials. In situ Raman spectra recorded from 800 to 1800 cm^−1^ during the eNO_3_RR using the CoCu‐HeD/NGO catalyst at diverse potentials from 0 to −0.5 V vs. RHE, along with the deconvoluted Raman spectrum at −0.4 V vs. RHE, highlighting the vibrational features of intermediate species during the eNO_3_RR, are depicted in Figure S38a,b. Raman bands observed at ∼1380 and ∼1640 cm^−1^ are attributed to adsorbed NH_3_ species formed during the reaction. Increasingly negative potentials cause intensification of these peaks, indicating a high yield of NH_3_ (Figure S38a). Deconvolution of the in situ Raman spectrum obtained at −0.4 V vs. RHE reveals two additional peaks at 1552 and 1624 cm^−1^, consistent with surface‐adsorbed HNO intermediates and adsorbed water molecules, respectively (Figure S38b) [74, 75]. This suggests that HNO is a key intermediate in the eNO_3_RR pathway, with adsorbed water molecules involved in subsequent hydrogenation steps. Notably, the absence of characteristic Raman peaks for metal oxides and hydroxides confirms the structural and chemical stability of CoCu‐HeD/NGO even at increasingly negative potentials. Further insights were obtained from ex situ FTIR spectroscopy to complement the in‐situ observation. Three‐dimensional FTIR spectra and the corresponding contour plot from 800 to 4000 cm^−1^ for the catholyte after 1 h of the eNO_3_RR using CoCu‐HeD/NGO at diverse potentials from 0 to −0.5 V vs. RHE, along with the deconvoluted FTIR spectrum at −0.4 V vs. RHE for two different spectral ranges, are displayed in Figure S39a–c. Distinct peaks at 3473 and 1632 cm^−1^ resemble to O─H stretching and H─O─H bending vibrations, respectively, confirming H_2_O molecules adsorbed on the catalyst surface, indicative of water dissociation (Figure S39a) [76]. In addition, deconvoluted FTIR spectra reveal vibrational fingerprints corresponding to the sequential deoxygenation and hydrogenation of NO_3_^−^ to NH_3_ (Figure S39b,c) [77, 78, 79]. These include characteristic signals for NO_2_^−^, NO, HNO, and NH*_x_* intermediates, strongly supporting the proposed eNO_3_RR reaction pathway: NO_3_ → NO_3_H → NO_2_ → NO_2_H → NO → HNO → H_2_NO → H_3_NO → NH_3_. These findings experimentally validate the DFT‐predicted mechanism and establish the pivotal role of water activation and surface‐bound intermediates in selective and efficient eNO_3_RR on the CoCu‐HeD/NGO catalyst.

### 2.6 DFT Calculations

**FIGURE 5:**
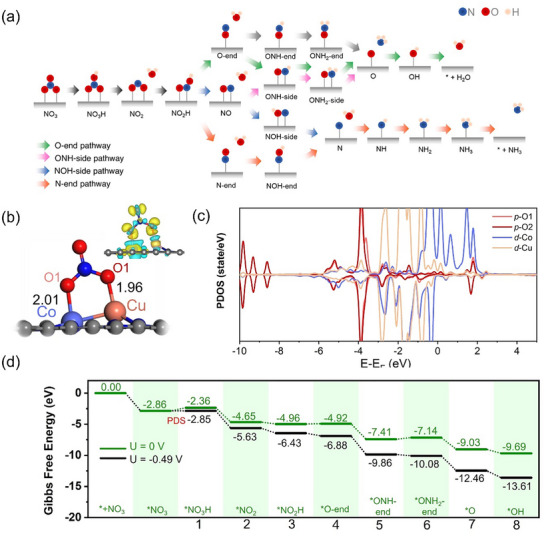
(a) Schematic depiction of the potential reaction pathways for the eNO_3_RR. (b) Most stable optimized structural models of NO_3_^−^ adsorption on the CoCu‐HeD/NGO catalyst. (c) PDOS analysis for NO_3_^−^ adsorption on the CoCu‐HeD/NGO catalyst. (d) Gibbs free energy diagrams of the most favorable eNO_3_RR pathways on the CoCu‐HeD/NGO catalyst.

To comprehensively understand the eNO_3_RR on the CoCu‐HeD/NGO catalyst, DFT calculations were conducted. The NGO catalyst was initially modeled as pristine graphene using a 7 × 7 × 1 supercell, then modified to create an NGO substrate suitable for metal doping. Subsequently, Co and Cu atoms were incorporated onto the surface of NGO to construct the CoCu‐HeD/NGO model (Figure S40), forming an M–M–L_2_‐coordinated CoCu heterodimer catalyst with pseudo‐D_3h_ symmetry. DFT calculations were used to investigate the eNO_3_RR pathways and elucidate the hydrogenation mechanism. As illustrated in Figure 5a, the eNO_3_RR process initiates with the chemisorption of NO_3_^−^ onto the active site, which is a critical step for high electrocatalytic efficiency. Figure 5b presents the most stable NO_3_^−^ ion adsorption configuration on the CoCu‐HeD/NGO catalyst, with a Gibbs free energy of −2.86 eV, indicating a strong affinity for NO_3_^−^. Charge density variation and PDOS confirm this robust interaction, revealing localized charge redistribution and orbital hybridization between the Cu and Co 3*d*‐orbitals of the catalyst and the O 2*p*‐orbitals of NO_3_^−^ (Figure 5c). These findings underscore the strong binding and activation of NO_3_^−^ on the CoCu‐HeD/NGO catalyst.

NO is a critical intermediate in the eNO_3_RR, with its adsorption configuration—either end‐on or side‐on—critically influencing subsequent hydrogenation pathways. As depicted in Figure 5a, three initial NO adsorption configurations were considered: O‐end, N‐end, and NO‐side. The NO‐side configuration can further evolve into two distinct orientations, yielding NOH‐side and ONH‐side pathways. Thus, the eNO_3_RR mechanism comprises four pathways: O‐end, N‐end, NOH‐side, and ONH‐side, each ultimately forming NH_3_. The eNO_3_RR is a highly complex process with multiple competing reactions; hence, an efficient catalyst must strongly adsorb NO_3_^−^ while operating at low overpotential. Herein, we comprehensively analyze reaction intermediates to evaluate the CoCu‐HeD/NGO catalytic performance during the eNO_3_RR. Gibbs free energy changes for elementary steps are summarized in Figure 5d and Figures S41–S44. The limiting potential (*U*_L_) for each pathway is determined by the largest Gibbs free energy change among the individual steps (*U*_L_ = −Δ*G*_max_/e^−^). Our results indicate that the eNO_3_RR on the CoCu‐HeD/NGO catalyst favors the O‐end pathway, following the sequence NO_3_(g) → *NO_3_ → *NO_3_H → *NO_2_ → *NO_2_H + *ON → *ONH → *ONH_2_ → *O → *OH. This pathway exhibits a Gibbs free energy change of only 0.49 eV (*U*_L_ = −0.49 V), which is slightly higher than that reported in our previous study [46]. Importantly, the potential‐determining step is the initial protonation step (NO_3_ + H⁺ + e^−^ → NO_3_H), which is similar to that on carbon‐based supported materials [80]. In aqueous media, the HER often competes, potentially diminishing the FE of the eNO_3_RR. To assess the selectivity of the CoCu‐HeD/NGO catalyst, we investigated the adsorption behavior of H^+^ on its surface (Figure S45). An ideal HER catalyst has |ΔG_*H_| close to 0 eV; strongly negative ΔG_*H_ (<0 eV) indicates excessively strong *H adsorption, which hinders hydrogen desorption and subsequently lowers the HER efficiency [81]. Our calculated ΔG_*H_ value of −0.58 eV is notably more negative than for the eNO_3_RR, suggesting lower intrinsic HER activity under the same electrochemical conditions. This result confirms the high selectivity of the CoCu‐HeD/NGO catalyst toward the eNO_3_RR over the HER.

### 2.7 Zn─NO_3_^−^ Battery and Practical NH_3_ Trapping Analysis

**FIGURE 6:**
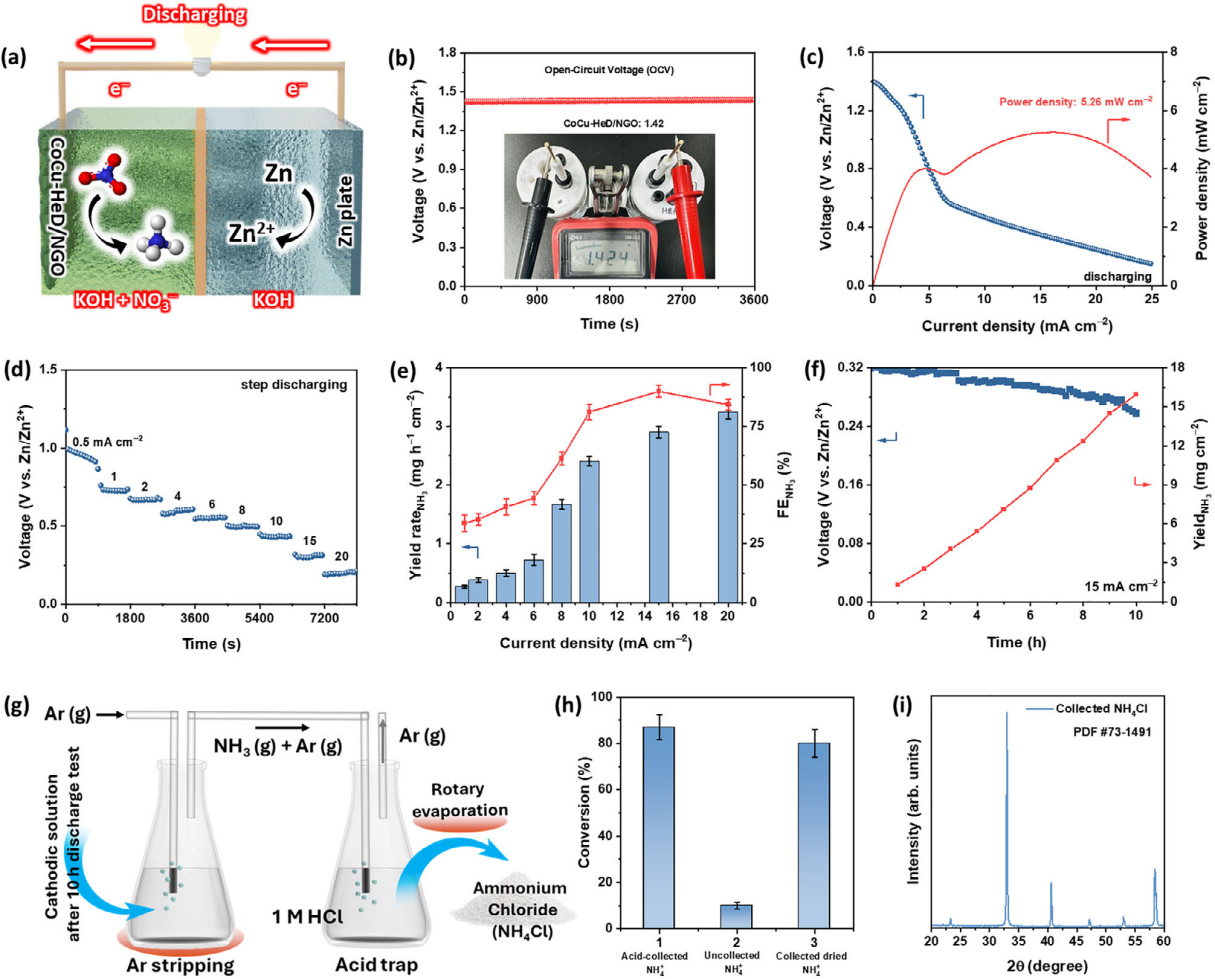
(a) Schematic representation of Zn─NO_3_^−^ battery with CoCu‐HeD/NGO cathode in 3.0 M KOH (anolyte) and 1.0 M KOH/0.1 M KNO_3_ (catholyte); (b) OCV test of the Zn–NO_3_^−^ battery with CoCu‐HD/NGO cathode; (c) discharge polarization curve and corresponding power density; (d) discharge profiles at different current densities; (e) NH_3_ yield rates and FE obtained at different discharging current densities; (f) long‐term discharge performance of the assembled Zn─NO_3_^−^ battery at 15 mA cm^−2^ for 10 h with NH_3_ yield; (g) schematic illustration of NH_3_ capture and conversion to solid NH_4_Cl during discharge at 15 mA cm^−2^; (h) conversion efficiency at each step of NH_3_ synthesis and recovery process; and (i) XRD pattern confirming the formation and purity of NH_4_Cl collected from acid trap.

Based on the excellent eNO_3_RR performance of CoCu‐HeD/NGO, a Zn–NO_3_^−^ galvanic cell was constructed by coupling the eNO_3_RR at the cathode with Zn oxidation at the anode, enabling simultaneous electricity generation and NH_3_ production (Figure 6a) [82, 83, 84, 85]. As shown in Figure 6b, the assembled Zn–NO_3_^−^ battery with CoCu‐HeD/NGO cathode maintained a stable OCV of 1.42 V vs. Zn/Zn^2+^ for 1 h. Discharge polarization analysis revealed a maximum power density of 5.26 mW cm^−2^ in 3.0 M KOH anolyte, outperforming those in 1.0 M KOH (1.22 mW cm^−2^) and 6.0 M KOH (4.68 mW cm^−2^), as depicted in Figure 6c and Figure S46. The superior performance of the Zn–NO_3_^−^ battery in 3.0 M KOH compared to 1.0 and 6.0 M KOH is attributed to the optimal balance between ionic conductivity and mass transport. At 1.0 M KOH, limited ionic conductivity hinders ion mobility, reducing the current output. Although 6.0 M KOH offers a higher ionic strength, its increased viscosity impairs mass transport and slows reaction kinetics via diffusion limitations. By contrast, 3.0 M KOH provides sufficient ionic conductivity with moderate viscosity, enabling efficient Zn oxidation and water dissociation, collectively enhancing the eNO_3_RR and overall power output. This power output is sufficient to drive low‐power electronic devices, highlighting the practical potential of the assembled cell. As demonstrated in Figure S47, the Zn─NO_3_^−^ battery continuously powered an electronic clock for 5 h. Furthermore, the time‐dependent discharge test presented in Figure 6d demonstrates the electrochemical Zn─NO_3_^−^ battery performance across a range of *j* from 0.5 to 20 mA cm^−2^. At a discharge *j* of 1 mA cm^−2^, the output voltage remained stable, gradually decreasing at elevated current densities, indicating robust rate capability. NH_3_ yield and FE at different *j* were measured using the colorimetric indophenol blue process (Figure 6e and Figure S48). At a discharge *j* of 15 mA cm^−2^, the Zn─NO_3_^−^ battery displayed a high FE of 89.97% with a corresponding NH_3_ YR of 2.9 mg h^−1^ cm^−2^. The long‐term galvanostatic discharge profile at 15 mA cm^−2^ (Figure 6f and Figure S49) showed a linear increase in NH_3_ yield over 10 h with stable FE (Figure S50) and minimal voltage decay, confirming the outstanding stability of the constructed Zn─NO_3_^−^ battery. Furthermore, the produced NH_3_ was efficiently converted into NH_4_Cl powder using an acid‐trapping method to demonstrate its practical applicability (Figure 6g and Figure S51). As shown in Figure 6h, ∼80% of NH_3_ vapor was successfully captured as NH_4_Cl (11.97 mg cm^−2^), and the purity of the product was confirmed via XRD analysis (Figure 6i). Overall, the Zn─NO_3_^−^ battery assembled with CoCu‐HeD/NGO demonstrates a promising sustainable energy‐conversion platform that simultaneously enables green NH_3_ synthesis and electricity generation.

## 3 Conclusions

We report the first use of a PLIL process to fabricate M–M–L_2_‐configured CoCu‐HeD/NGO with a pseudo‐D_3h_ symmetric structure, demonstrating a tandem catalytic mechanism for the selective eNO_3_RR to NH_3_. The synergistic interaction among Co and Cu sites enables the spatial separation of proton generation and NO_3_^−^ activation, following an LH‐type hydrogenation pathway. This strategy effectively overcomes conventional scaling limitations, accomplishing a high FE of 91% and an exceptional NH_3_ yield. In situ Raman spectroscopy, ex situ FTIR spectroelectrochemistry, and theoretical analyses exposed that the Co─Cu atomic interface functions as an electronic bridge, optimizing adsorption energies of key intermediates (H* and NO_3_^−^), thereby enhancing the eNO_3_RR kinetics and long‐term stability. Furthermore, the integration of the CoCu‐HeD/NGO catalyst into a Zn─NO_3_^−^ battery demonstrates its multifunctionality for simultaneous energy generation, NH_3_ synthesis, and NO_3_^−^ pollutant removal, underscoring the potential of atomic‐precision heterodimer catalysts in sustainable energy and environmental applications.

## Conflicts of Interest

The authors declare no conflicts of interest.

## Supporting information




**Supporting File**: advs73497‐sup‐0001‐SuppMat.docx.

## Data Availability

The data that support the findings of this study are available from the corresponding author upon reasonable request.
