# Supplementary material for: Atomic Precision CoCu Heterodimers with Pseudo‐D3h Symmetry Enable Tandem Nitrate Reduction
Source: Adv Sci (Weinh). 2025 Dec 22;13(10):e23909. doi: 10.1002/advs.202523909 (PMC12915090; doi:10.1002/advs.202523909)
Supplement: Supplementary file 1 — Supporting File: advs73497‐sup‐0001‐SuppMat.docx. [file ADVS-13-e23909-s001.docx]

**Supporting Information**

**for**

**Atomic Precision CoCu Heterodimers with Pseudo-D_3h_ Symmetry Enable Tandem Nitrate Reduction**

Akash Prabhu Sundar Rajan ^a,†^, Jayaraman Theerthagiri ^a,†^, Piyapa Junmon ^b^, Wanwisa Limphirat ^c^*,* Nuttapon Yodsin ^b,^*, Myong Yong Choi ^a,d,^*

^a^ Department of Chemistry (BK21 FOUR), Research Institute of Advanced Chemistry, Gyeongsang National University, Jinju 52828, Republic of Korea

^b^ Department of Chemistry, Faculty of Science, Silpakorn University, Nakhon Pathom, 73000 Thailand

^c^ Beamline Division, Synchrotron Light Research Institute (SLRI), Nakhon Ratchasima, 30000 Thailand

^d^ Core-Facility Center for Photochemistry & Nanomaterials, Gyeongsang National University, Jinju 52828, Republic of Korea

Correspondence: [yodsin_n@su.ac.th](mailto:yodsin_n@su.ac.th) (N. Yodsin); [mychoi@gnu.ac.kr](mailto:mychoi@gnu.ac.kr) (M.Y. Choi)

† These authors contributed equally to this work

**Table of Contents**

**SI. Experiment section**

SI.1 Chemicals

SI.2 Synthesis of CoCu-HeD/NGO via PLIL

SI.3 Characterization

SI.4 X-ray absorption spectroscopy measurements

SI.5 Electrochemical NO_3_^‒^ reduction reaction measurements

SI.6 Zn-nitrate battery

SI.7 Determination of NH_3_ product via indophenol blue method

SI.8 Determination NO_2_^−^ concentration using Griess method

SI.9 ^1^H nuclear magnetic resonance studies

SI.10 Faradaic efficiency and yield rate of NH_3_

SI.11 Calculation of the cathodic energy efficiency

SI.12 In situ Raman spectroelectrochemistry measurements

S1.13 Computational method

**Figures:**

**Figure S1.** FESEM image with EDS mapping of (a) GO, and (b) NGO.

**Figure S2.** (a) XPS survey spectra of GO and NGO; Core-level spectra of (b) C 1*s*, (c) O 1*s,* and (d) N 1*s* of GO and N-GO.

**Figure S4.** Intensity histogram profiles at different site of CoCu-HeD/NGO

**Figure S5.** XRD pattern of pure GO, NGO, Cu_2_-HoD/NGO, Co_2_-HoD/NGO, and CoCu-HeD/NGO.

**Figure S6.** Raman spectra of GO, NGO, Cu_2_-HoD/NGO, Co_2_-HoD/NGO, and CoCu-HeD/NGO.

**Figure S7.** FESEM image with EDS mapping of (a) Cu_2_-HoD/NGO and (b) Co_2_-HoD/NGO.

**Figure S8.** (a) Low- and high-magnification HAADF-STEM image with EDS elemental mapping of Cu_2_-HoD/NGO; (b) HAADF-STEM image of Cu_2_-HoD/NGO showing bright spots corresponding to hetero-single-atom dimers (marked by yellow circles); corresponding 3D-contour plots of Cu_2_-HoD/NGO highlighting dual-atom interactions.

**Figure S9.** (a) Low- and high-magnification HAADF-STEM image with EDS elemental mapping of Co_2_-HoD/NGO; (b) HAADF-STEM image of Co_2_-HoD/NGO showing bright spots corresponding to hetero-single-atom dimers (marked by yellow circles); corresponding 3D-contour plots of Co_2_-HoD/NGO highlighting dual-atom interactions.

**Figure S10.** XPS (a) Survey spectra of Cu_2_-HoD/NGO, Co_2_-HoD/NGO, and CoCu-HeD/NGO; Core-level spectra of (b) C 1*s*, (c) O 1*s*, and (d) N 1*s* of Cu-SA/NGO, Co-SA/NGO, and CoCu-HeD/NGO.

**Figure S11.** XPS Core-level spectra of (a) Co 2*p* and (b) Cu 2*p* of Cu-SA/NGO, Co-SA/NGO, and CoCu-HeD/NGO.

**Figure S12.** Bader charge analysis of the CoCu dimer anchored on nitrogen-doped graphene oxide (NGO). Charge density distribution showing the quantitative Bader charge on each atom, confirming electron donation from NGO to the dimer. Corresponding bar graph illustrating the Bader charge distribution across the Co, Cu, and NGO regions, highlighting interatomic and interfacial charge redistribution within the CoCu/NGO system.

**Figure S13.** XANES spectra and pre-edge features of as-prepared Co_2_-HoD/NGO, Cu_2_HoD/NGO, and CoCu-HeD/NGO.

**Figure S14.** Determination of oxidation states of as-prepared samples by half-edge jump in XANES energy (a) Co *k*-edge of Co_2_-HoD/NGO and CoCu-HeD/NGO, and (b) Cu *k*-edge of Cu_2_-HoD/NGO and CoCu-HeD/NGO.

**Figure S15.** WT images of the (a) Co *k*-edge from Co_2_-HoD/NGO, and (b) Cu *k*-edge from Co_2_-HoD/NGO.

**Figure S16.** Fitting results of the EXAFS spectra of (a) Co_2_-HoD/NGO in R space of Co *k*-edge and (b) Cu_2_-HoD/NGO in R space of Cu *k*-edge with M-M-L_2_ coordination configuration.

**Figure S17.** Fitting results of the EXAFS spectra of (a) Co_2_-HoD/NGO, (b) Cu_2_-HoD/NGO, and (c & d) CoCu-HeD/NGO in R space of Co *k*-edge and Cu *k*-edge with M-L_4_ coordination configuration.

**Figure S18.** Illustrating the pseudo-D_3h_ structure of Co_2_-HoD/NGO, Cu_2_-HoD/NGO.

**Figure S19.** UV-vis calibration curves for determining NH_3_ using ammonium chloride solutions of known concentrations as standards: (a) UV-vis spectra using the indophenol blue method with NH_3_, and (b) concentration-absorbance calibration curve at 651 nm for different NH_3_ concentrations.

**Figure S20.** Electrocatalytic performance of eNO_3_RR: (a) LSV and (b) Tafel slope in acidic and alkaline anolyte of for CoCu-HeD/NGO.

**Figure S21.** Electrolysis profile at different potential for CoCu-HeD/NGO with (a) alkaline and (b) acidic anolyte.

**Figure S22.** Comparing NH_3_ (a) yield rate, and (b) FE for CoCu-HeD/NGO with acidic and alkaline anolyte.

**Figure S23.** One-hour of continuous eNO_3_RR electrolysis profiles of (a) NGO, (b) Cu_2_-HoD/NGO, (c) Co_2_-HoD/NGO, and (d) CoCu-HeD/NGO at different potentials.

**Figure S24.** UV-visible absorbance spectra of NH_3_ produced during eNO_3_RR electrolysis using (a) NGO, ((b) Cu_2_-HoD/NGO, (c) Co_2_-HoD/NGO, and (d) CoCu-HeD/NGO at different potentials.

**Figure S25.** NMR calibration curves for determining NH_4_^+^ using ammonium chloride solutions of known concentrations as standards: (a) NMR spectra with different concentration of NH_4_^+^_,_ and (b) concentration-integral area calibration curve for different NH_4_^+^ concentrations.

**Figure S26.** (a) The ^1^H NMR spectra for the catholyte after 1 h of eNO_3_RR electrolysis under different potential with CoCu-HD/NGO; and (b) corresponding NH_4_^+^ yield rate comparing with UV-vis results obtained via indophenol blue method.

**Figure S27.** Core level XPS spectra of (a) Co 2*p*, and (b) Cu 2*p* for CoCu-HeD/NGO electrode before and after eNO_3_RR cyclic stability for 12 h.

**Figure S28.** UV-vis calibration curves for determining NO_2_^‒^ using KNO_2_ solutions of known concentrations as standards: (a) UV-vis spectra using the indophenol blue method with NO_2_^‒^_,_ and (b) concentration-absorbance calibration curve at 540 nm for different NO_2_^‒^ concentrations.

**Figure S29.** UV-visible absorbance spectra of NO_2_^‒^ produced during eNO_3_RR electrolysis using (a) NGO, (b) Cu_2_-HoD/NGO, (c) Co_2_-HoD/NGO, and (d) CoCu-HeD/NGO at different potentials.

**Figure S30.** Comparison of (a) yield rate and (b) FE for NH_3_ and NO_2_^‒^ during eNO_3_RR using NGO.

**Figure S31.** Comparison of (a) yield rate and (b) FE for NH_3_ and NO_2_^‒^ during eNO_3_RR using Cu_2_-HoD/NGO.

**Figure S32.** Comparison of (a) yield rate and (b) FE for NH_3_ and NO_2_^-^ during eNO_3_RR using Co_2_-HoD/NGO.

**Figure S33.** Comparison of (a) yield rate and (b) FE for NH_3_ and NO_2_^‒^ during eNO_3_RR using CoCu-HeD/NGO.

**Figure S34.** CV profiles of the NGO, Cu_2_-HoD/NGO, Co_2_-HoD/NGO, and CoCu-HeD/NGO catalysts with a scan rate of 50 mV s^−1^ in 1.0 M KOH solution: (a) without and (b) with NO_3_^−^ addition.

**Figure S35.** Nyquist plots for (a) Cu_2_-HoD/NGO, (b) Co_2_-HoD/NGO, and (c) CoCu-HeD/NGO catalysts at various potentials in 1.0 M KOH solution without NO_3_^−^ addition.

**Figure S36.** CV curves of (a) NGO, (b) Cu_2_-HoD/NGO, (c) Co_2_-HoD/NGO, and (d) CoCu-HeD/NGO catalysts in the non-Faradic regions from 0.1 to 0.3 V vs. RHE at different scan rates from 20-100 mV s^−1^ to estimate C_dl_ values.

**Figure S37.** Double-layer capacitance (C_dl_) plots for various catalysts calculated at 0.2 V vs. RHE using different scan rate profiles at non-faradic region.

**Figure S38.** (a) In situ Raman spectra in the range of 800 – 1800 cm^1^ at various potentials from 0 to ‒0.5 V vs. RHE, and (b) deconvoluted Raman spectrum at 0.4 V vs. RHE, highlighting the evolution of vibrational features associated with intermediate species during eNO_3_RR using CoCu-HeD/NGO catalyst.

**Figure S39.** (a) Three-dimensional FTIR spectra and corresponding contour plot in the range of 800 – 4000 cm^1^ for the catholyte obtained after 1 h of eNO_3_RR using CoCu-HeD/NGO at various potentials from 0 to ‒0.5 V vs. RHE, and (b and c) deconvoluted FTIR spectrum at 0.4 V vs. RHE for two different spectral ranges.

**Figure S40.** Schematic representation of the (a) side view with lattice constant, and (b) top view of the CoCu-HeD/NGO catalyst. Atom colors: purple for Co, rose gold for Cu, blue for N, and grey for C.

**Figure S41**. (a and b) The Gibbs free energy diagrams for eNO_3_RR on the CoCu-HeD/NGO catalyst through O-end pathway.

**Figure S42**. (a and b) The Gibbs free energy diagrams for eNO_3_RR on the CoCu-HeD/NGO catalyst through ONH-side pathway.

**Figure S43**. (a and b) The Gibbs free energy diagrams for eNO_3_RR on the CoCu-HeD/NGO catalyst through NOH-side pathway.

**Figure S44**. (a and b) The Gibbs free energy diagrams for eNO_3_RR on the CoCu-HeD/NGO catalyst through N-end pathway.

**Figure S45**. (a and b) The Gibbs free energy diagrams for HER on the CoCu-HeD/NGO catalyst through N-end pathway.

**Figure S46.** (a) Discharge polarization profiles and (b) corresponding power density plot of the assembled Zn-NO_3_^‒^ battery with CoCu-HeD/NGO cathode at different anolyte concentrations (1.0, 3.0, and 6.0 M KOH).

**Figure S47.** Photograph of an electronic timer powered by assembled Zn-NO_3_^‒^ battery with CoCu-HeD/NGO cathode for 5 h.

**Figure S48.** UV-vis absorbance spectra of NH_3_ produced during discharging of the Zn-NO_3_^‒^ battery with CoCu-HeD/NGO cathode under various current densities**.**

**Figure S49.** Time-dependent UV-vis spectra of NH_3_ produced at fixed time interval during discharging for 10 h of the Zn-NO_3_^‒^ battery with CoCu-HeD/NGO cathode at 15 mA cm^‒2^.

**Figure S50.** Calculated FE% for long-term durability test of the assembled Zn-NO_3_^‒^ battery with CoCu-HeD/NGO cathode at 15 mA cm^‒2^ for 10 h

**Figure S51.** Picture represents instrumental setup used for NH_3_ capture via acid trap method.

**Tables:**

**Table S1**. Comparison of elemental composition weight percentage of CoCu-HeD/NGO obtained from EDS and ICP-OES analyses.

**Table S2.** EXAFS structure parameters for different samples.

**Table S3.** EIS circuit fitting parameters for all catalysts.

**Table S4.** Comparison table for the eNO_3_RR performance.

**References**

**SI. Experiment section**

**SI.1 Chemicals**

Copper(II) acetate monohydrate (Cu(CH_3_COO)_2_·H_2_O, 99.0%), Cobalt(II) acetate tetrahydrate (Co(CH_3_COO)_2_·4H_2_O, ≥98.0%), sodium nitroprusside dihydrate (Na_2_[Fe(CN)_5_NO]·2H_2_O, ≥99%), sulfanilamide (H_2_NC_6_H_4_SO_2_NH_2_, ≥98%), N-(1-naphthyl) ethylenediamine dihydrochloride (C_10_H_7_NHCH_2_CH_2_NH_2_.2HCl, >98%), Nafion (5 wt%) solution, salicylic acid (C_7_H_6_O_3_, ≥99%), ammonium chloride (NH_4_Cl, ≥99.5%), and graphene oxide flake (GO powder) were purchased from Sigma Aldrich, USA. Ammonium hydroxide (NH_4_OH, 25-28%), absolute ethanol (C_2_H_5_OH, ≥99.9%), sodium citrate dihydrate (C_6_H_5_Na_3_O_7_·2H_2_O, ≥99%), sodium hydroxide (NaOH, ≥97.0%), potassium hydroxide flake (KOH, ≥ 93%), hydrogen peroxide (H_2_O_2_, 30%), and sulfuric acid (H_2_SO_4_, ≥98%) were obtained from Daejung Chemicals, South Korea. Potassium nitrate (KNO_3_, ≥99%) and sodium hypochlorite solution (NaClO, 6% – 14%) were procured from Samchun Chemicals, South Korea.

**SI.2 Synthesis of CoCu-HeD/NGO via PLIL**

To synthesis the homo and heterodimer, an effective and eco-friendly pulsed laser irradiation in liquids (PLIL) technique was employed. In a typical synthesis of CoCu-HeD/NGO, a fixed amount of commercial graphene oxide (GO, 5 mg) was dispersed in a mixed solvent of deionized water and ethanol (10 mL, 1:1 v/v) along with 5 mL of ammonium hydroxide (NH_4_OH). Subsequently, 0.4 mg each of cobalt and copper salts (Cu(CH_3_COO)_2_·H_2_O and Co(CH_3_COO)_2_·4H_2_O) were added to the mixture and ultrasonicated to ensure homogeneous dispersion of the metal precursors. The resulting solution was subjected to PLI using a Nd:YAG Surelite II-10 laser (wavelength: 1064 nm, pulse width: 7 ns, repetition rate: 10 Hz, energy: 150 mJ) for 30 min under continuous magnetic stirring, ensuring uniform irradiation and promoting three critical physicochemical processes: (i) partial reduction of GO, (ii) N doping, and (iii) the immobilization and stabilization of CoCu heterodimer on N-doped GO (NGO) substrate. After PLIL, the resulting CoCu heterodimer (CoCu-HeD/NGO) was centrifuged at 14,000 rpm for 10 min, washed with absolute ethanol, and vacuum-dried at 60°C for 3 h. For comparison, NGO was synthesized under identical conditions without the addition of metal salts. The corresponding Co and Cu homodimers (Co_2_-HoD/NGO and Cu_2_-HoD/NGO) were prepared using only the respective metal salt precursors under the same PLIL conditions.

**SI.3 Characterization**

The crystalline structure, morphology, and microstructure of the samples were investigated using X-ray diffraction (XRD, Bruker D2 Phaser) with Cu Kα radiation (λ = 1.54 Å) and field-emission scanning electron microscopy (FESEM, FEI Quanta 450 FEG). The surface chemical states and elemental composition of the as-prepared samples were analyzed by X-ray photoelectron spectroscopy (XPS, ESCALAB 250) using an Al Kα X-ray ray source (E = 1486.6 eV). UV–visible spectra were recorded using a Lambda 2S UV/Vis spectrometer. High-angle annular dark-field scanning transmission electron microscopy (HAADF-STEM) and corresponding energy-dispersive X-ray spectroscopy (EDS) elemental mapping were conducted using a Cs-corrected transmission electron microscope (JEOL JEM-ARM200F NEOARM). The metal compositions of both homo- and heterodimer catalysts were determined by inductively coupled plasma optical emission spectroscopy (ICP-OES, iCAP PRO XP Duo, Thermo Fisher Scientific). Fourier-transform infrared (FTIR) spectra were collected in attenuated total reflection (ATR) mode using a Nicolet iS10 FTIR spectrometer (Thermo Fisher Scientific).

**SI.4 X-ray absorption spectroscopy measurements**

X-ray absorption spectroscopy (XAS) measurements, including both X-ray absorption near-edge structure (XANES) and extended X-ray absorption fine structure (EXAFS), were performed at Beamline 8 of the Synchrotron Light Research Institute (SLRI), Thailand. All spectra were collected in fluorescence mode using a Vortex ME4 13-element silicon drift detector. Energy calibration was conducted using the first derivative of Co (7709 eV) and Cu (8979 eV) metal foils for K-edge alignment. The XAS data were processed, averaged, and normalized using the Demeter software package (version 0.9.18.2). Fourier transforms of the k^2^-weighted EXAFS spectra were performed in the k-range of 3–9 Å⁻^1^.

**SI.5 Electrochemical NO_3_^‒^ reduction reaction measurements**

The electrochemical NO_3_^‒^ reduction reaction (eNO_3_RR) experiments were conducted in a two-compartment H-type cell, where the cathodic chamber contained 1.0 M KOH/0.1 M KNO_3_ and the anodic chamber contained 0.5 M H_2_SO_4_. The two chambers were separated by a Nafion 117 proton exchange membrane (N-117, dupont). All electrochemical measurements were performed under ambient conditions using a CHI760E electrochemical workstation. To prepare the catalyst ink, 1.0 mg of the catalyst was dispersed in a mixture of ethanol (95 μL), deionized water (95 μL), and 10.0 μL of Nafion solution (5 wt.%), followed by sonication for 30 min to obtain a homogeneous dispersion. The resulting ink was drop-cast onto a 1 × 1 cm^2^ piece of carbon cloth and dried at room temperature for 3 h. The catalyst-coated carbon cloth (CC), a Hg/HgO (1.0 M KOH) reference electrode, and a graphite rod counter electrode were used as the working, reference, and counter electrodes, respectively. All measured potentials were converted to the reversible hydrogen electrode (RHE) scale using the Nernst equation:

E_RHE_ = E_0_ + E_Hg/HgO_ + 0.059 × pH

To reach a steady-state condition, the working electrode was subjected to 100 cyclic voltammetry (CV) cycles at a scan rate of 50 mV s^‒1^ in 1.0 M KOH. Linear sweep voltammetry (LSV) was performed from 0 to ‒0.6 V vs. RHE at a scan rate of 5 mV s^‒1^ with magnetic stirring at 300 rpm. Electrochemical impedance spectroscopy (EIS) was conducted over a frequency range of 100 kHz to 0.1 Hz at various potentials in 1 M KOH. Chronopotentiometric measurements were carried out at fixed potentials ranging from ‒0.1 to ‒0.5 V vs. RHE for 1 h in the H-cell to evaluate the Faradaic efficiency and yield rate of NH_3_. Post-electrolysis, the concentration of NH_3_ was quantified using UV–visible spectrophotometry.

**SI.6 Zn-nitrate battery**

The catalyst-loaded CC (1×1 cm^2^) and a Zn plate (1×1 cm^2^) were employed as the cathode and anode, respectively, in the Zn–nitrate (Zn-NO_3_**^‒^**) battery system. A standard H-type electrochemical cell was used, with the cathodic chamber containing 30 mL of 0.1 KNO_3_ + 1.0 M KOH, and the anodic chamber filled with 3.0 M KOH electrolyte. The two chambers were separated by a Nafion 117 proton exchange membrane to maintain ionic conductivity while preventing electrolyte crossover. All battery-related electrochemical measurements were conducted at room temperature using a CHI 760E electrochemical workstation and a WBCS3000L battery test system.

The power density (P) of Zn-NO_3_**^‒^** battery was determined by P = I ×V, where I and V are the discharge current density and voltage, respectively.

The electrochemical reactions in Zn-NO_3_**^‒^** battery is presented as following:

Cathode reaction: NO_3_^‒^ + 7H_2_O + 8e^‒^ → NH_4_OH + 9OH^‒^

Anode reaction: 4Zn + 8OH^‒^ → 4ZnO + 4H_2_O + 8e^‒^

Overall reaction: 4Zn + NO_3_^‒^ + 3H_2_O → 4ZnO + NH_4_OH + OH^‒^

**SI.7 Determination of NH_3_ product via indophenol blue method**

The yield of electrosynthesized NH_3_ during the eNO_3_RR and Zn-NO_3_**^‒^** battery experiments was quantified using the indophenol blue method. To minimize experimental error, all measurements were performed in triplicate, and the results were presented with error bars. Specifically, 50 μL of electrolyte was collected after the eNO_3_RR and battery tests, followed by the addition of 2 mL of a colorimetric reagent solution containing 1 M NaOH, 5 wt% salicylic acid, and 5 wt% sodium citrate. Subsequently, 1 mL of NaClO (0.05 M in H_2_O) and 200 μL of sodium nitroferricyanide solution (0.1 wt%) were added. The mixture was kept at ambient conditions for 2 h to allow complete color development. UV–vis absorption spectra were recorded, and the absorbance was measured at 658 nm. A calibration curve was generated using standard NH_3_ solutions prepared from NH_4_Cl in 1.0 M KOH. The absorbance values of all test samples were background-corrected by subtracting the absorbance of a blank sample without NH_3_.

**SI.8 Determination NO_2_^−^ concentration using Griess method**

The concentration of the secondary product, nitrite (**NO_2_^−^**) formed during the eNO_3_RR was measured using UV–vis spectrophotometry following the standard Griess method. The colorimetric reagent was prepared by dissolving sulfanilamide, N-(1-naphthyl)ethylenediamine dihydrochloride, and phosphoric acid in deionized water to a final volume of 50 mL. Electrolyte samples were appropriately diluted to fall within the detection range. Then, 200 μL of the diluted sample was mixed with 1 mL of the prepared Griess reagent, followed by the addition of 5 mL of deionized water. The mixture was allowed to react at room temperature for 20 min. The absorbance was measured at 540 nm using a UV–vis spectrophotometer. A calibration curve was constructed by treating standard KNO_2_ solutions of known concentrations under the same conditions. The concentration of NO_2_^‒^ in the test samples was determined using the linear fit of the calibration curve.

**SI.9 ^1^H nuclear magnetic resonance studies**

The formation and quantification of NH_3_ was further confirmed using ^1^H NMR (300 MHz, Bruker Avance-III 300 NMR). For the NMR analysis, 600 μL of catholyte obtained after 60 min of eNO_3_RR using HE-RSO at a fixed potential of −0.2 and −0.4 V vs. RHE was mixed with 600 μL mL of D_2_O. Subsequently, the pH of the mixture was adjusted to approximately 2 (acidic) by adding 0.5 M H_2_SO_4_. The prepared solution was then transferred to 5 mm NMR tubes for analysis. The recorded ^1^H NMR spectrum displayed three characteristic peaks corresponding to ^14^NH_4_^+^. The concentrations unknown ^14^NH_4_^+^ were quantified using a calibration curve generated from standard NH_4_Cl solutions (200, 400, and 600 ppm).

**SI.10 Faradaic efficiency and yield rate of NH_3_**

The faradaic efficiency (FE) of NH_3_ production was determined by the following equation:

𝐹𝐸(𝑁𝐻_3_) = (8×F×𝐶×V)/(M×Q) × 100%

Where F is Faraday constant (96485 C mol^−1^), C_NH3_ is the concentration of NH_3_ (μg mL^−1^) in the electrolyte, V is the volume of the electrolyte, Q is the charge consumed for NH_3_ generation (C). M is the relative molecular mass of NH_3_ (17.031 g mol^−1^)

The yield rate (YR, mg h^−1^ cm^−2^) of NH_3_ can be calculated using the following equation:

𝑌𝑅(𝑁𝐻_3_) = (𝐶×V)/(t×A)

Where C_NH3_ is the concentration of NH_3_ (μg mL^−1^) in the electrolyte, V is the volume of the electrolyte, t is the electrolysis time, and A is the geometric area of the electrode (~1 cm^2^).

**SI.11 Calculation of the cathodic energy efficiency**

The half-cell energy efficiency (EE) is defined as the ratio of the chemical energy stored in the produced ammonia to the input electrical energy. It is calculated using the following equation: EE_NH3_ = ((1.23-E^0^_NH3_) × FE_NH3_)/(1.23-E), where E^0^_NH3_ represents the equilibrium potential of eNO_3_RR to NH_3_ (0.69 V vs. RHE), FE_NH3_ is the Faradaic efficiency for ammonia, and E is the applied potential vs. RHE. The value 1.23 V vs. RHE is the equilibrium potential for water oxidation (i.e. assuming the overpotential of the water oxidation is zero).

**SI.12 In situ Raman spectroelectrochemistry measurements**

To investigate real-time surface changes on the electrode, in-situ Raman spectroscopy measurements were performed using a three-electrode electrochemical Raman cell in an electrolyte containing 1.0 M KOH/0.1 M KNO_3_. The working electrode consisted of catalyst-coated carbon cloth, while a Hg/HgO electrode and a graphite rod were used as the reference and counter electrodes, respectively. Raman spectra were acquired using a Raman microscope (Thermo Fisher Scientific, DXR3) equipped with a 532 nm Nd:YAG laser operating at 7 mW. Spectra were recorded in the range of 800 to 1800 cm^−1^ during bulk electrolysis at various applied potentials ranging from 0 to ‒0.5 mV vs. RHE.

**S1.13 Computational method**

The density functional theory (DFT) calculations were performed using the Vienna Ab-initio Simulation Package (VASP) [1, 2]. The ion-valence electron interactions were described using projector augmented wave (PAW) potentials, while the exchange-correlation effects between electrons were treated using the generalized gradient approximation (GGA) with the Perdew-Burke-Ernzerhof (PBE) functional. [3]. The electronic eigenfunctions were expanded using a kinetic energy cutoff of 450 eV, along with a k-point grid for accurate Brillouin zone sampling. The Brillouin-zone integration was performed using a Γ-centered 3 × 3 × 1 Monkhorst−Pack k-point grid. The electronic relaxation was considered converged when the total energy change between two consecutive optimization steps fell below 1 × 10^⁻5^ eV. Additionally, ionic relaxation was deemed complete when the forces acting on atoms were reduced to less than 0.02 eV/Å. The dispersion-corrected DFT-D approach was utilized to account for van der Waals interactions, specifically incorporating the DFT-D3 method proposed by Grimme. [4, 5].

The NO_3_^−^ adsorption energy (E_ads_) characterizes the interaction between the surface and adsorbed species, which is defined as E_ads_ = E_sys_ - E_x_ - E_sur_, where E_sys_ represents the total energy of adsorbing system, E_x_ represents the energy of adsorb species and E_sur_ denotes the energy of pure surface. The Gibbs free energy change (ΔG) for each elementary step in the eNO_3_RR process was determined based on the widely adopted computational hydrogen electrode (CHE) model [6]: ΔG = ΔE + ΔE_ZPE_ - TΔS + ∫C_p_dT, where the reaction energy difference (ΔE) is directly obtained from DFT calculations. ΔE_ZPE_ represents the change in zero-point energies, TΔS is the change in entropy between the products and reactants at room temperature (T = 298.15 K), and ∫C_p_dT is the enthalpic temperature correction. These parameters can be derived from the vibrational frequency analysis of the adsorbed species.

To avoid complications with directly calculating the energy of the charged NO_3_^–^ species using DFT methods, gaseous HNO_3_ and H_2_ were chosen as reference points. The following reactions were analyzed (equation 1-3):

NO_3_^–^ (aq) + H^+^ → HNO_3_ (l) (1)

HNO_3_ (l) → HNO_3_ (g) (2)

* + HNO_3_(g) → *NO_3_ + H^+^ + e^−^ (3)

To investigate the adsorption mechanism, reaction 3 **(equation 3)** was first studied. Under conditions of pH = 0 and 1 bar of gaseous H_2_, the chemical potential of (H^+^ + e^−^) is equivalent to the free energy per hydrogen in the computational hydrogen electrode (CHE) model [6-8]. The ΔG_NO3*_ was determined as follows: ΔG_NO3*_ = G_NO3*_ – G_*_ – G_HNO3_(g) + 1/2G_H2_(g) + ΔG_correct_, where G_NO3*_, G_*_, G_HNO3_, and G_H2_ represent the Gibbs free energies of NO_3_^−^ adsorbed on the system, clean substrate, gaseous HNO_3_, and gaseous H_2_, respectively. In practical experiments, NO_3_^−^ is found in the aqueous phase, while HNO_3_ is in the liquid phase, as shown in reactions 1 and 2 (**equations 1 and 2**). As a result, solvation effects must be accounted for in the G_NO3*_ term. The Gibbs free-energy changes (ΔG_sol_) for reactions 1 and 2 are 0.317 eV and 0.075 eV, respectively [9]. These reactions facilitate the conversion from NO_3_^−^ in the aqueous phase to HNO_3_ in the gaseous phase, leading to the corrected G_NO3*_ calculation as follows: $\Delta G_{{*NO}_{3}}^{corr}= {\Delta G}_{{*NO}_{3}}+ {\Delta G}_{sol}= {\Delta G}_{{*NO}_{3}}+0.392$

In addition, the limiting potential (U_L_) can be obtained to evaluate the activity of a catalyst following the equation of U_L_ = -DG_max_*/* |*e*|, where DG_max_ is the relative change of the reaction with the maximum free energy change, and *e* is the charge of an electron [10]. The potential determining step (PDS) giving the U_L_ is the step with the largest ΔG value.


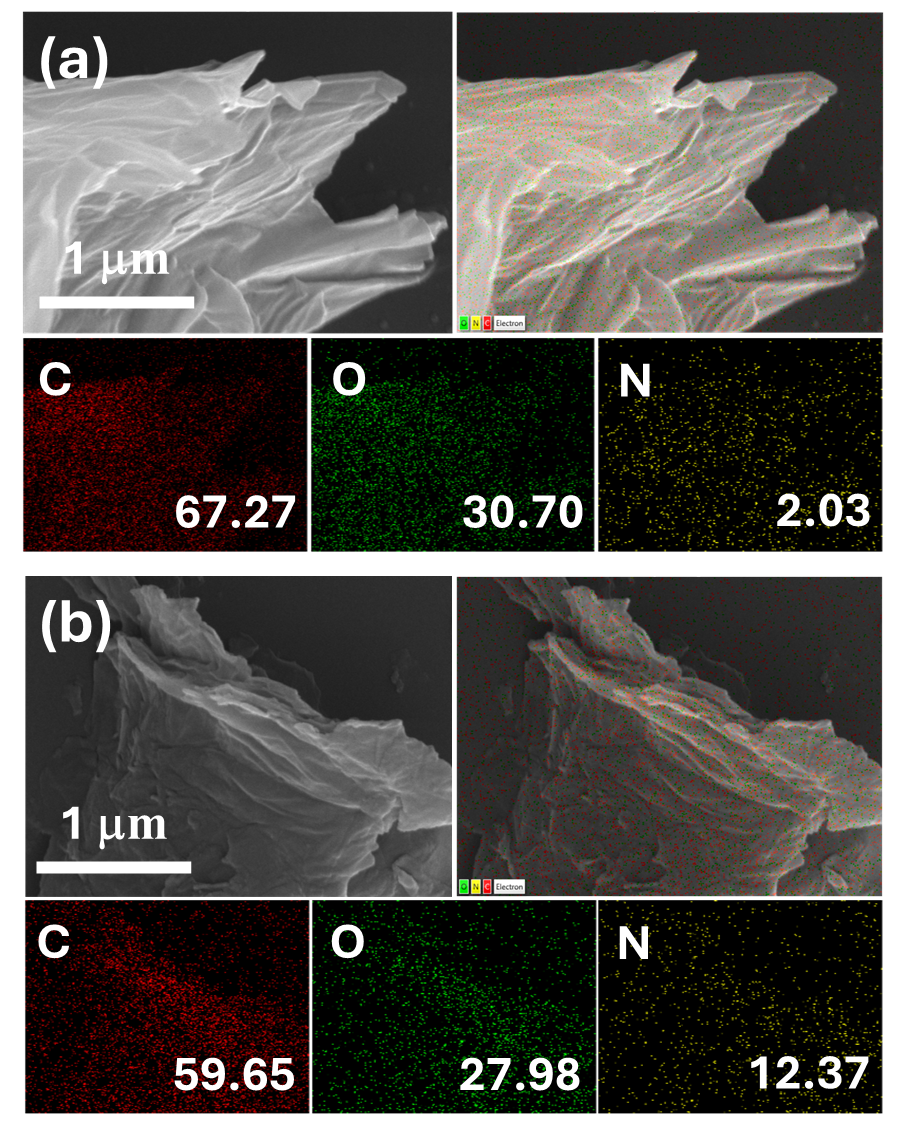


**Figure S1.** FESEM image with EDS mapping of (a) GO, and (b) NGO.


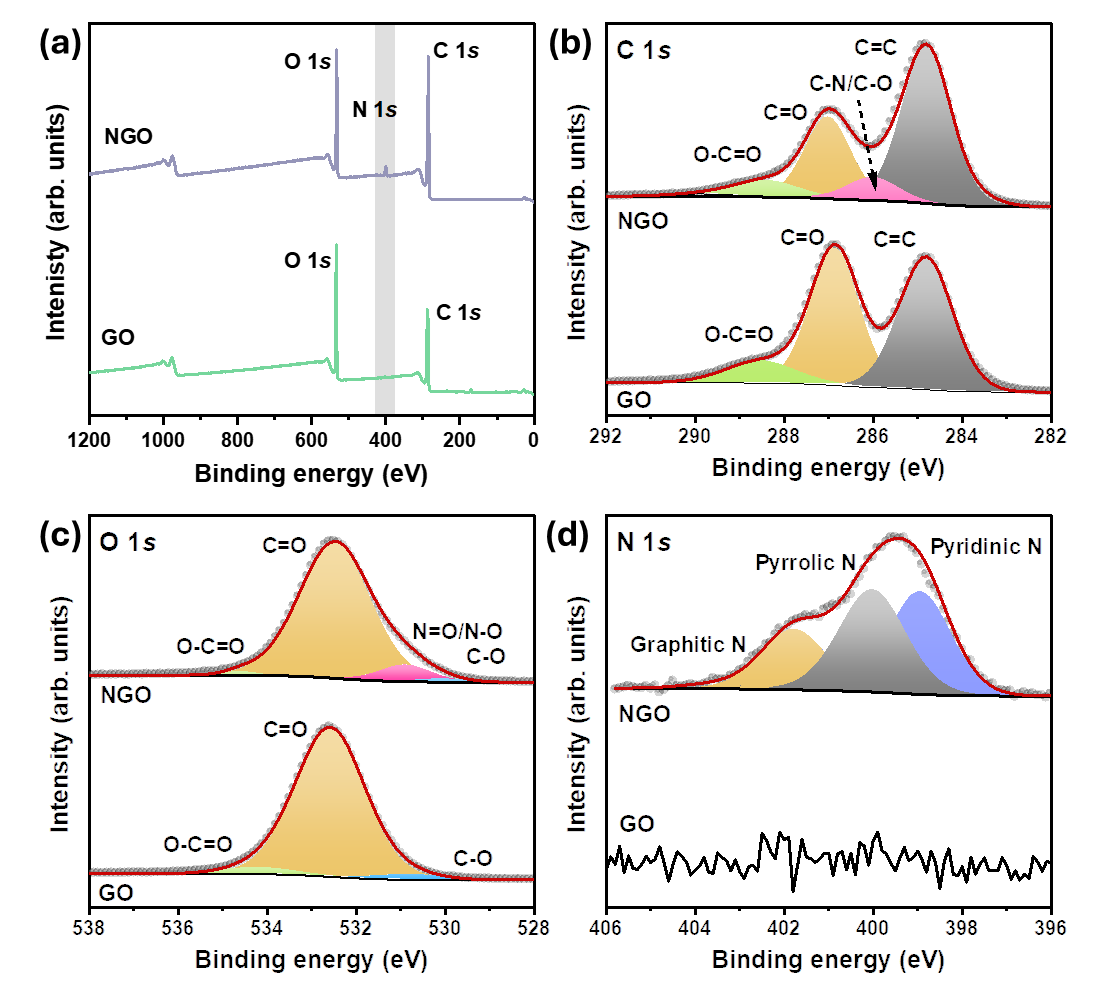


**Figure S2.** (a) XPS survey spectra of GO and NGO; Core-level spectra of (b) C 1*s*, (c) O 1*s,* and (d) N 1*s* of GO and N-GO.


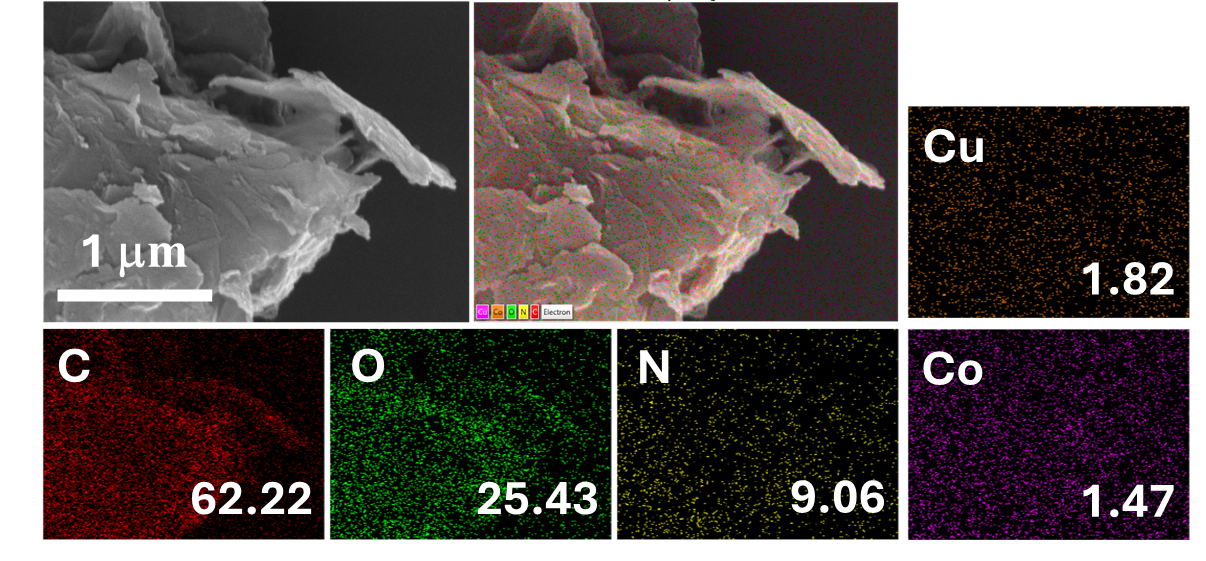


**Figure S3.** FESEM image with EDS mapping of CoCu-HeD/NGO.


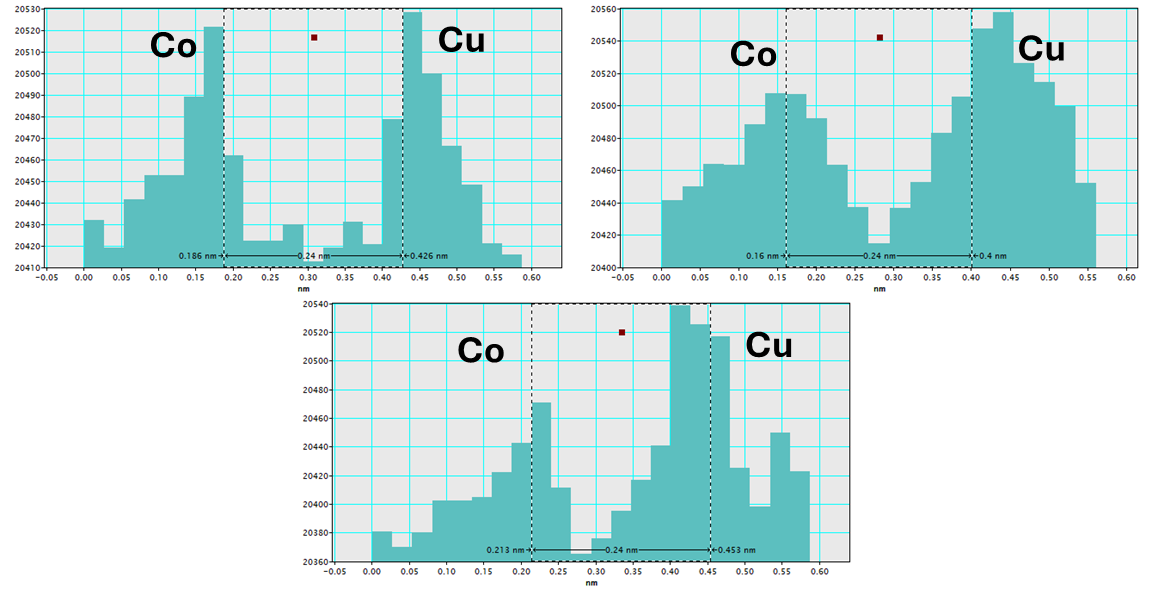


**Figure S4.** Intensity histogram profiles at different site of CoCu-HeD/NGO


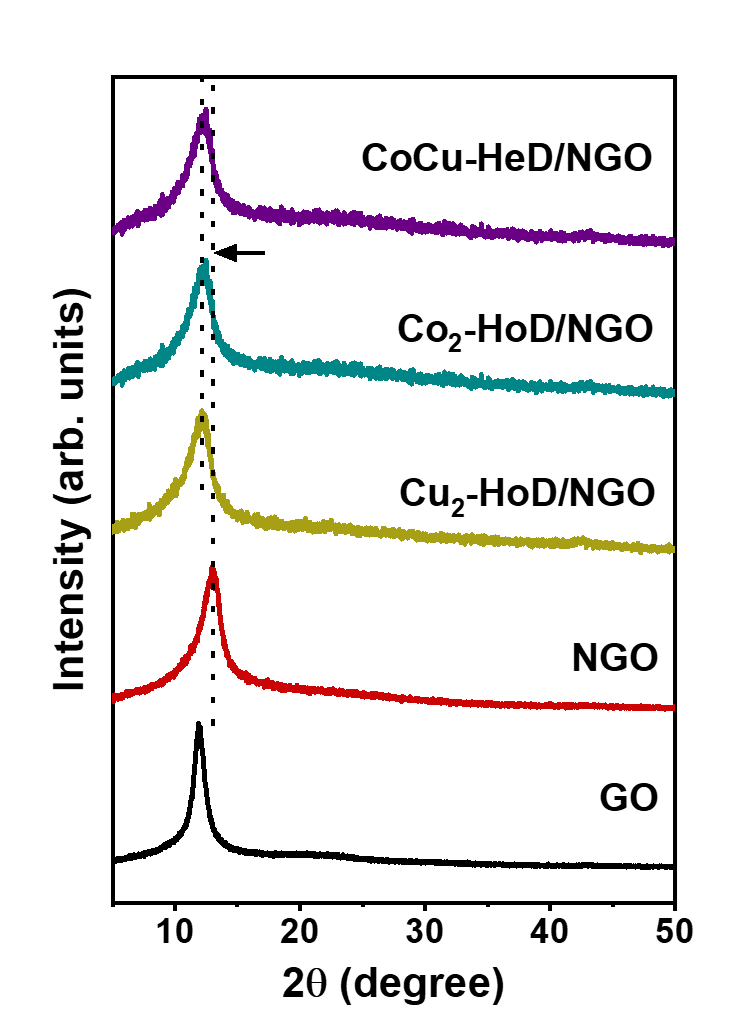


**Figure S5.** XRD pattern of pure GO, NGO, Cu_2_-HoD/NGO, Co_2_-HoD/NGO, and CoCu-HeD/NGO.


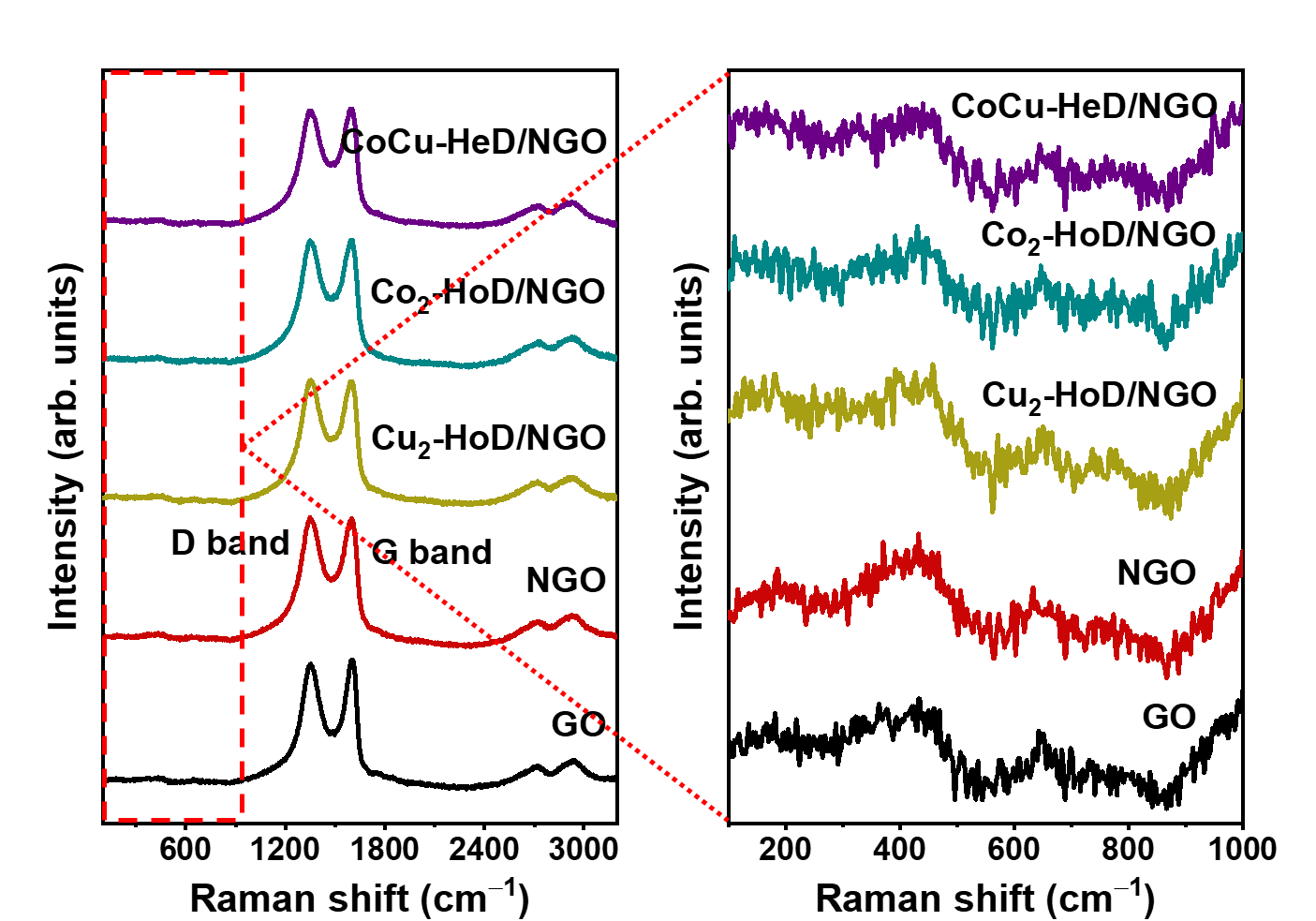


**Figure S6.** Raman spectra of GO, NGO, Cu_2_-HoD/NGO, Co_2_-HoD/NGO, and CoCu-HeD/NGO.


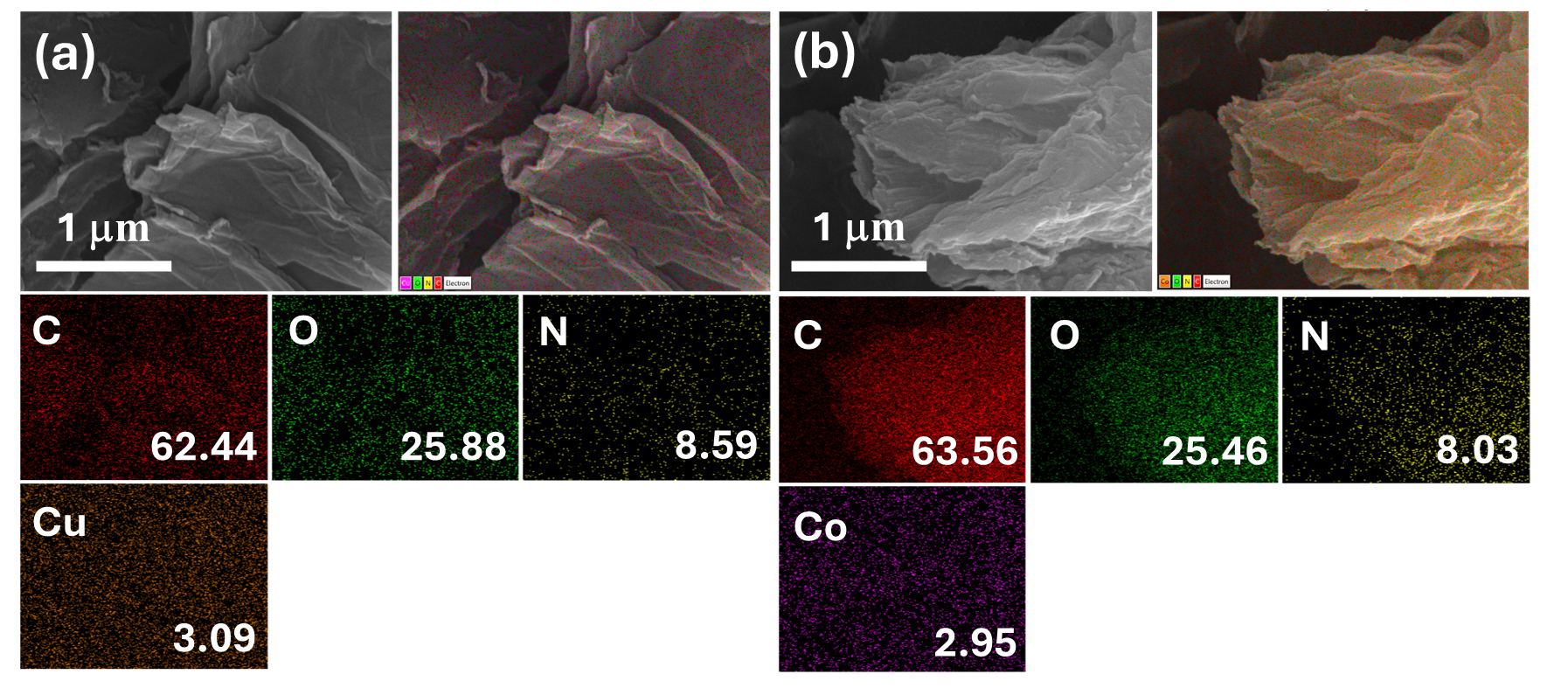


**Figure S7.** FESEM image with EDS mapping of (a) Cu_2_-HoD/NGO and (b) Co_2_-HoD/NGO.


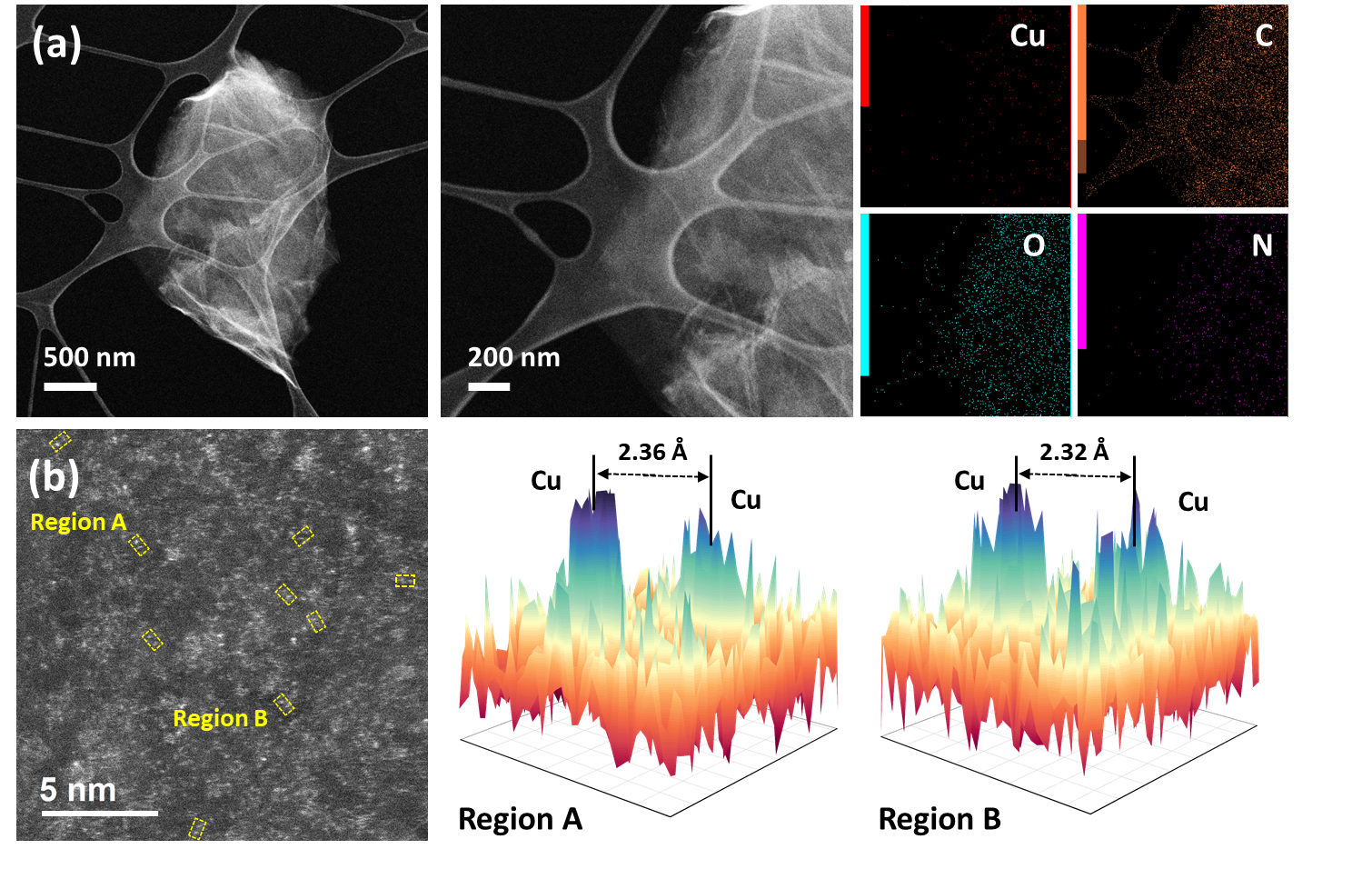


**Figure S8.** (a) Low- and high-magnification HAADF-STEM image with EDS elemental mapping of Cu_2_-HoD/NGO; (b) HAADF-STEM image of Cu_2_-HoD/NGO showing bright spots corresponding to hetero-single-atom dimers (marked by yellow circles); corresponding 3D-contour plots of Cu_2_-HoD/NGO highlighting dual-atom interactions.


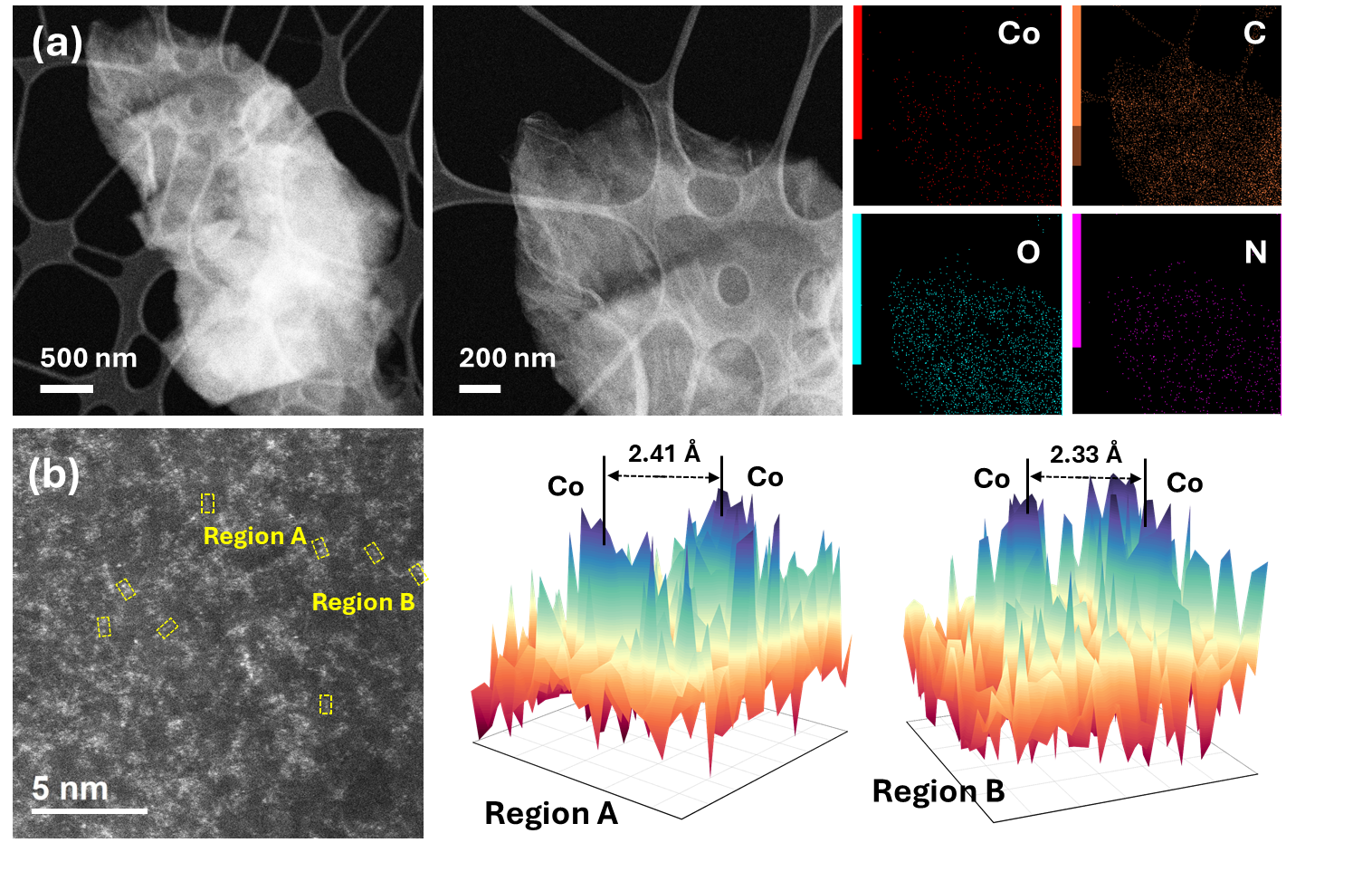


**Figure S9.** (a) Low- and high-magnification HAADF-STEM image with EDS elemental mapping of Co_2_-HoD/NGO; (b) HAADF-STEM image of Co_2_-HoD/NGO showing bright spots corresponding to hetero-single-atom dimers (marked by yellow circles); corresponding 3D-contour plots of Co_2_-HoD/NGO highlighting dual-atom interactions.


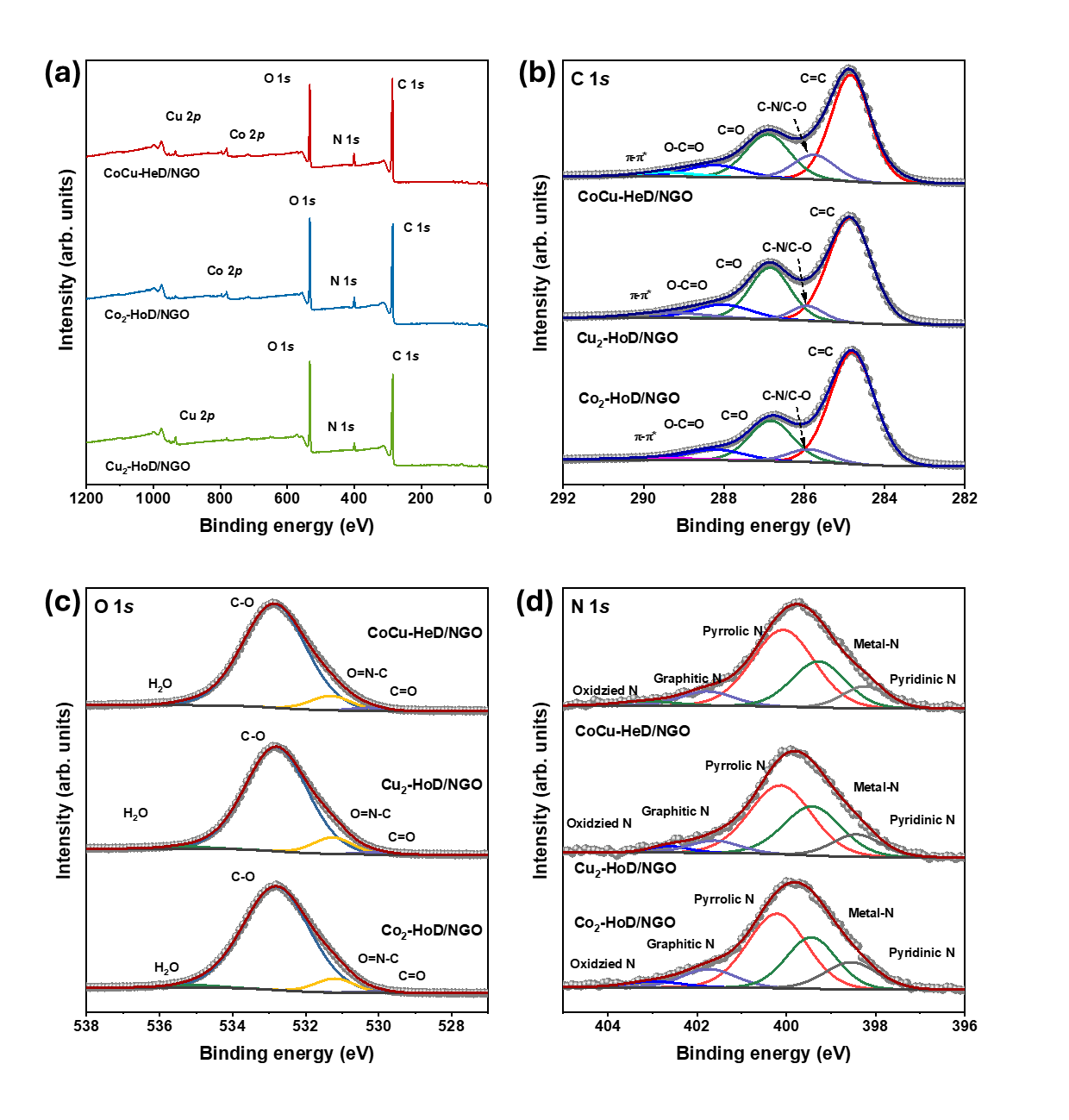


**Figure S10.** XPS (a) Survey spectra of Cu_2_-HoD/NGO, Co_2_-HoD/NGO, and CoCu-HeD/NGO; Core-level spectra of (b) C 1*s*, (c) O 1*s*, and (d) N 1*s* of Cu-SA/NGO, Co-SA/NGO, and CoCu-HeD/NGO.


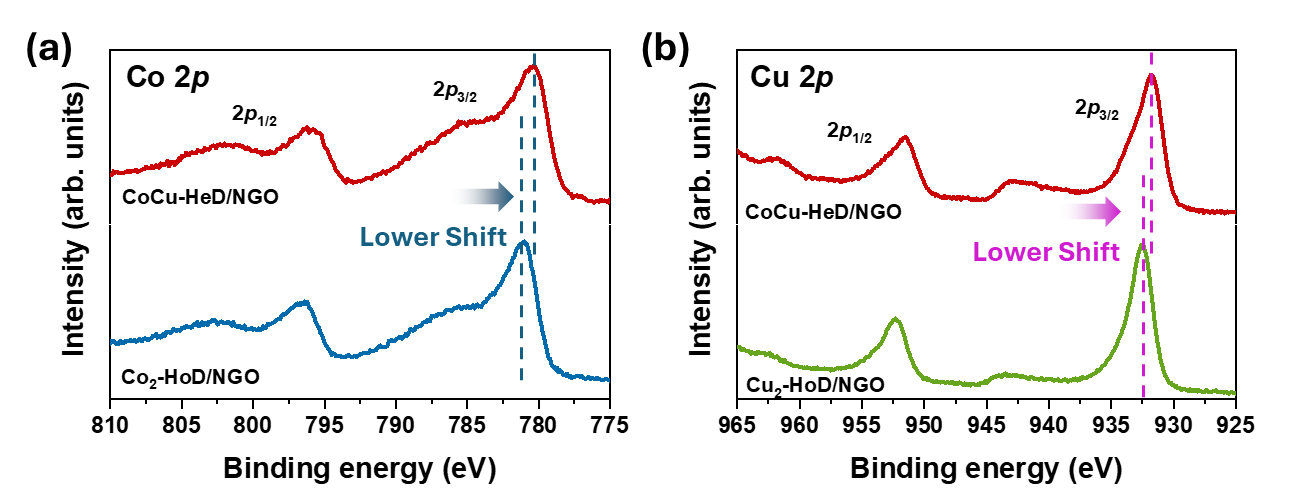


**Figure S11.** XPS Core-level spectra of (a) Co 2*p* and (b) Cu 2*p* of Cu-SA/NGO, Co-SA/NGO, and CoCu-HeD/NGO.


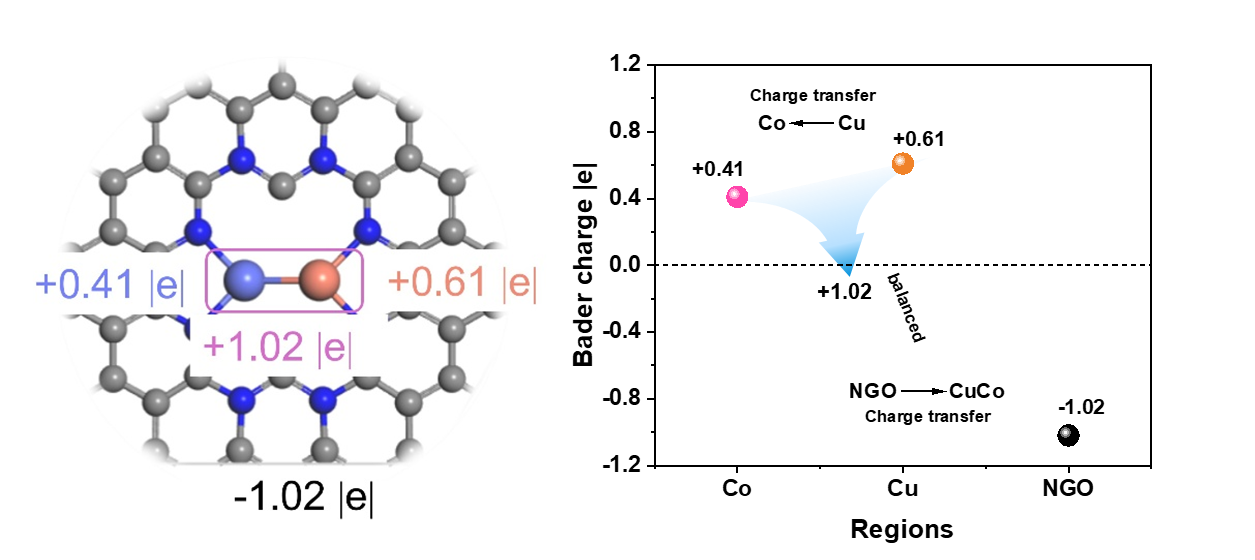


**Figure S12.** Bader charge analysis of the CoCu dimer anchored on nitrogen-doped graphene oxide (NGO). Charge density distribution showing the quantitative Bader charge on each atom, confirming electron donation from NGO to the dimer. Corresponding bar graph illustrating the Bader charge distribution across the Co, Cu, and NGO regions, highlighting interatomic and interfacial charge redistribution within the CoCu/NGO system.


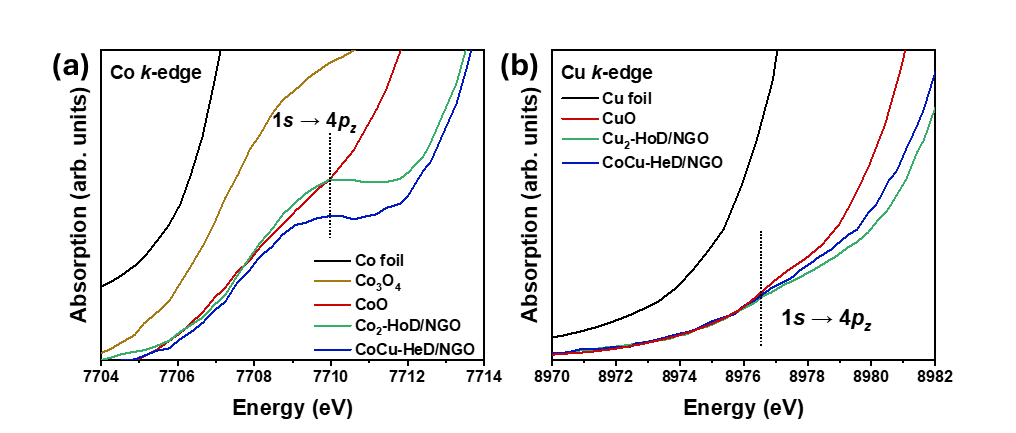


**Figure S13.** XANES spectra and pre-edge features of as-prepared Co_2_-HoD/NGO, Cu_2_HoD/NGO, and CoCu-HeD/NGO.


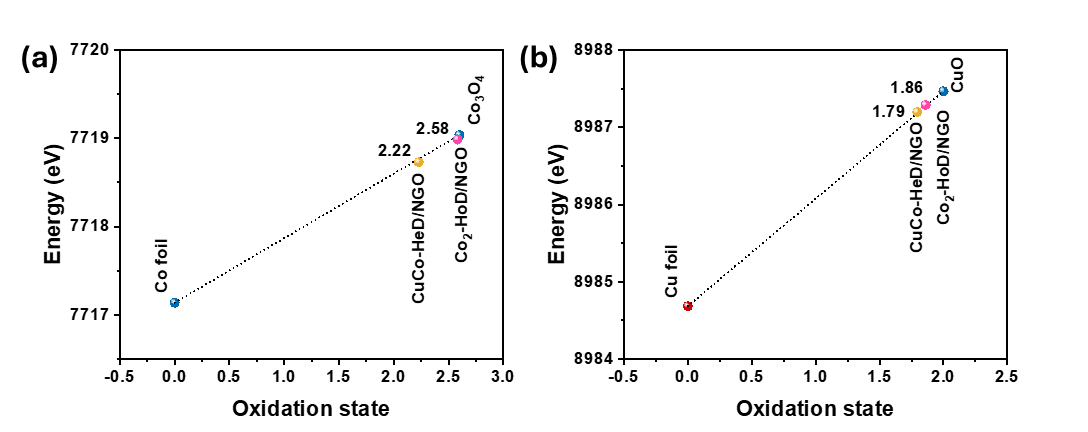


**Figure S14.** Determination of oxidation states of as-prepared samples with reference materials by half-edge jump in XANES energy (a) Co *k*-edge of Co_2_-HoD/NGO and CoCu-HeD/NGO, and (b) Cu *k*-edge of Cu_2_-HoD/NGO and CoCu-HeD/NGO.


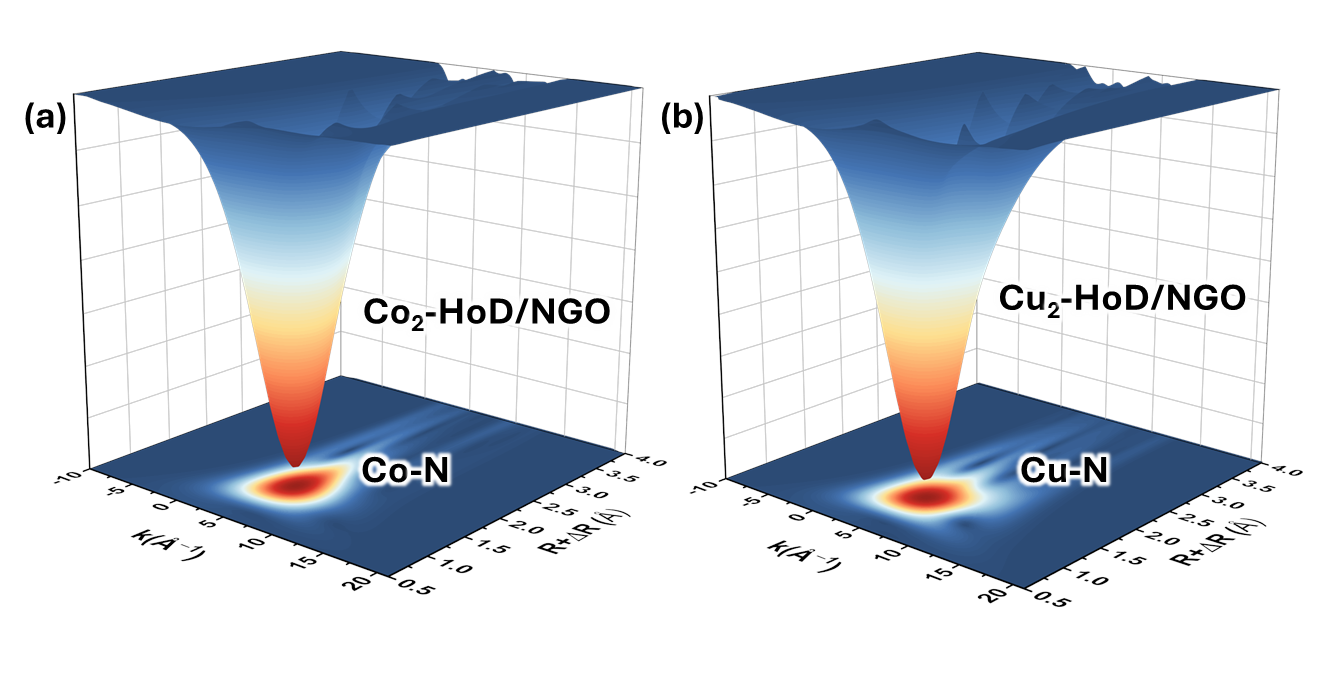


**Figure S15.** WT images of the (a) Co *k*-edge from Co_2_-HoD/NGO, and (b) Cu *k*-edge from Co_2_-HoD/NGO.


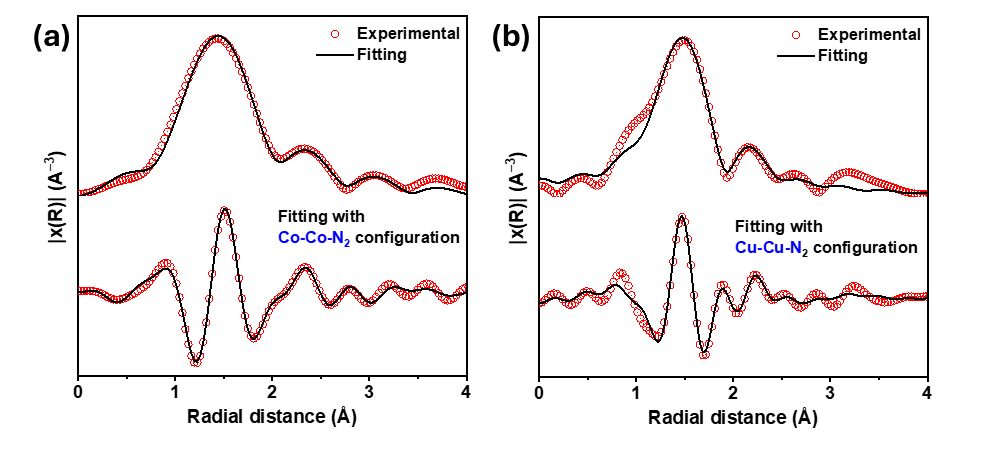


**Figure S16.** Fitting results of the EXAFS spectra of (a) Co_2_-HoD/NGO in R space of Co *k*-edge and (b) Cu_2_-HoD/NGO in R space of Cu *k*-edge with M-M-L_2_ coordination configuration.


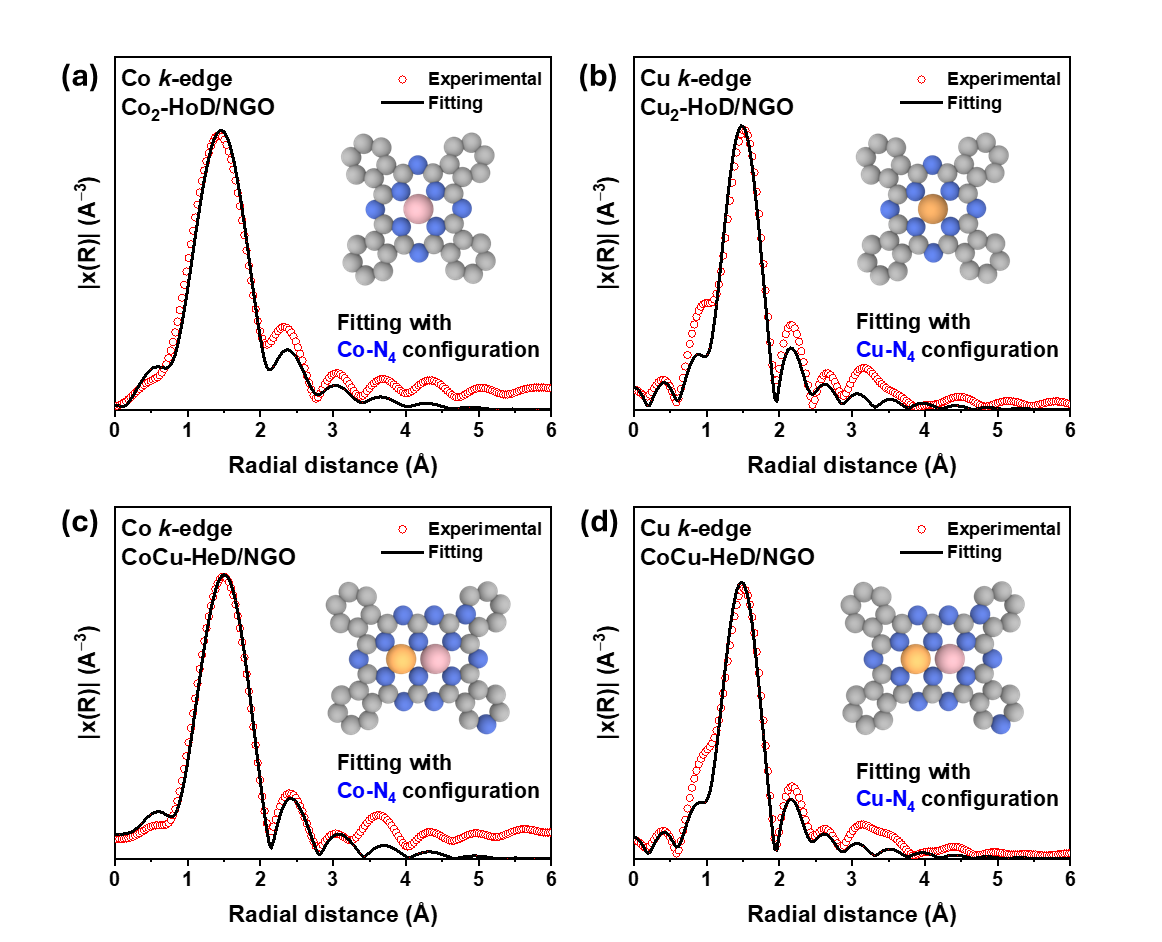


**Figure S17.** Fitting results of the EXAFS spectra of (a) Co_2_-HoD/NGO, (b) Cu_2_-HoD/NGO, and (c & d) CoCu-HeD/NGO in R space of Co *k*-edge and Cu *k*-edge with M-L_4_ coordination configuration.


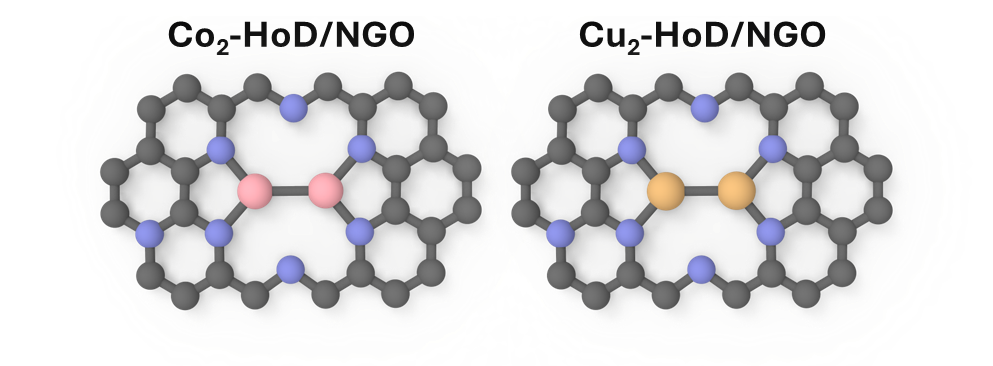


**Figure S18.** Illustrating the pseudo-D_3h_ structure of Co_2_-HoD/NGO, Cu_2_-HoD/NGO, and CoCu-HeD/NGO with three types of N in graphene oxide (N_1_-Pyridinic; N_2_-Pyrrolic; and N_3_-Graphitic).


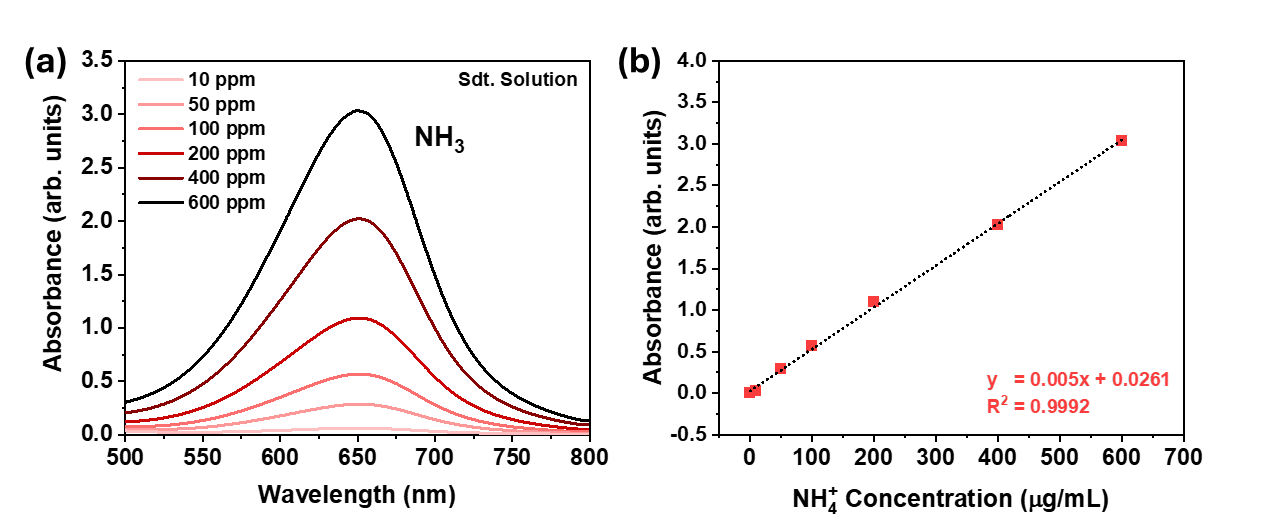


**Figure S19.** UV-vis calibration curves for determining NH_3_ using ammonium chloride solutions of known concentrations as standards: (a) UV-vis spectra using the indophenol blue method with NH_3_, and (b) concentration-absorbance calibration curve at 651 nm for different NH_3_ concentrations.


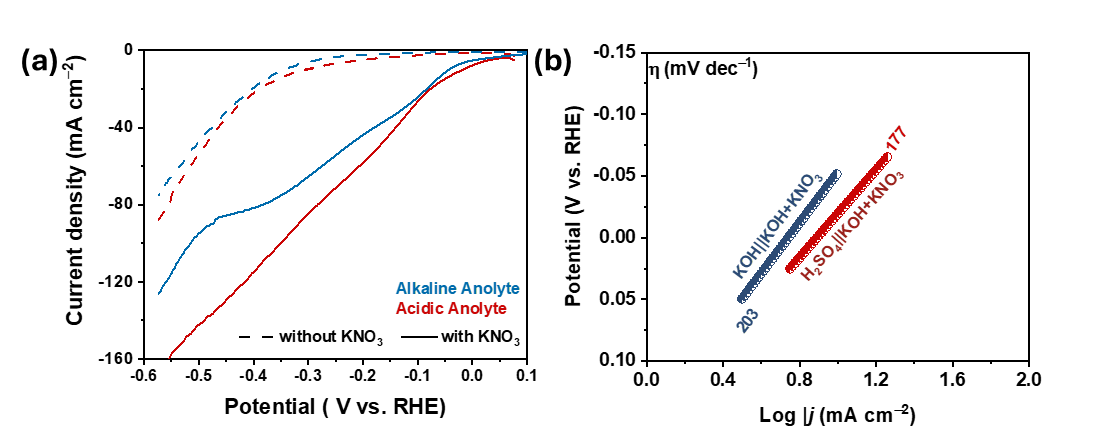


**Figure S20.** Electrocatalytic performance of eNO_3_RR: (a) LSV and (b) Tafel slope in acidic and alkaline anolyte of for CoCu-HeD/NGO.


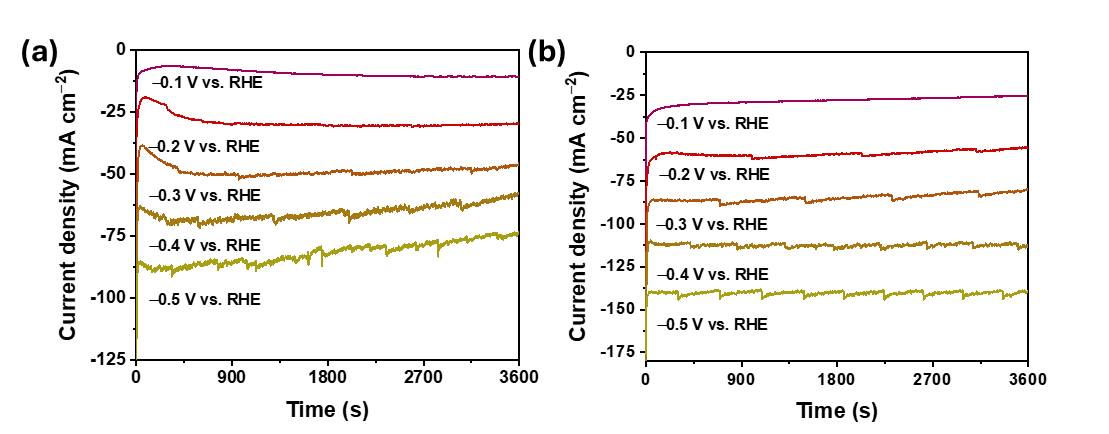


**Figure S21.** Electrolysis profile at different potential for CoCu-HeD/NGO with (a) alkaline and (b) acidic anolyte.


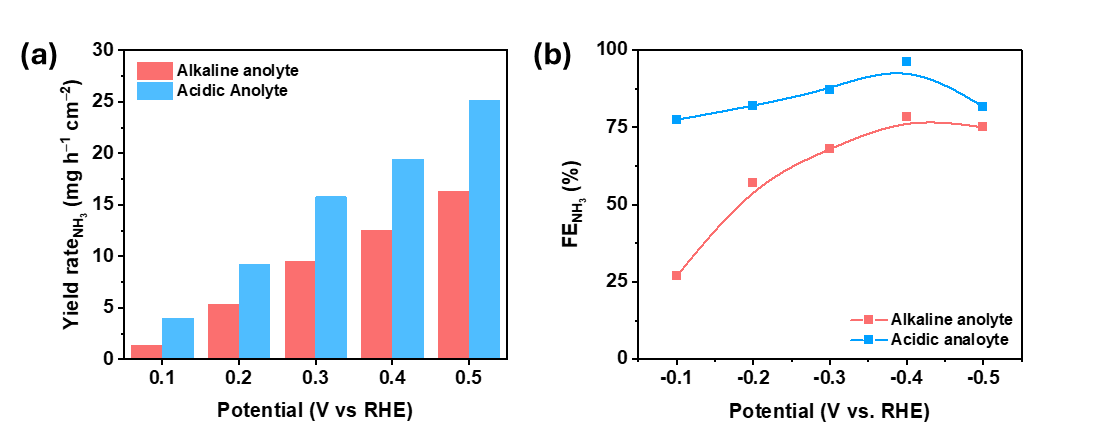


**Figure S22.** Comparing NH_3_ (a) yield rate, and (b) FE for CoCu-HeD/NGO with acidic and alkaline anolyte.


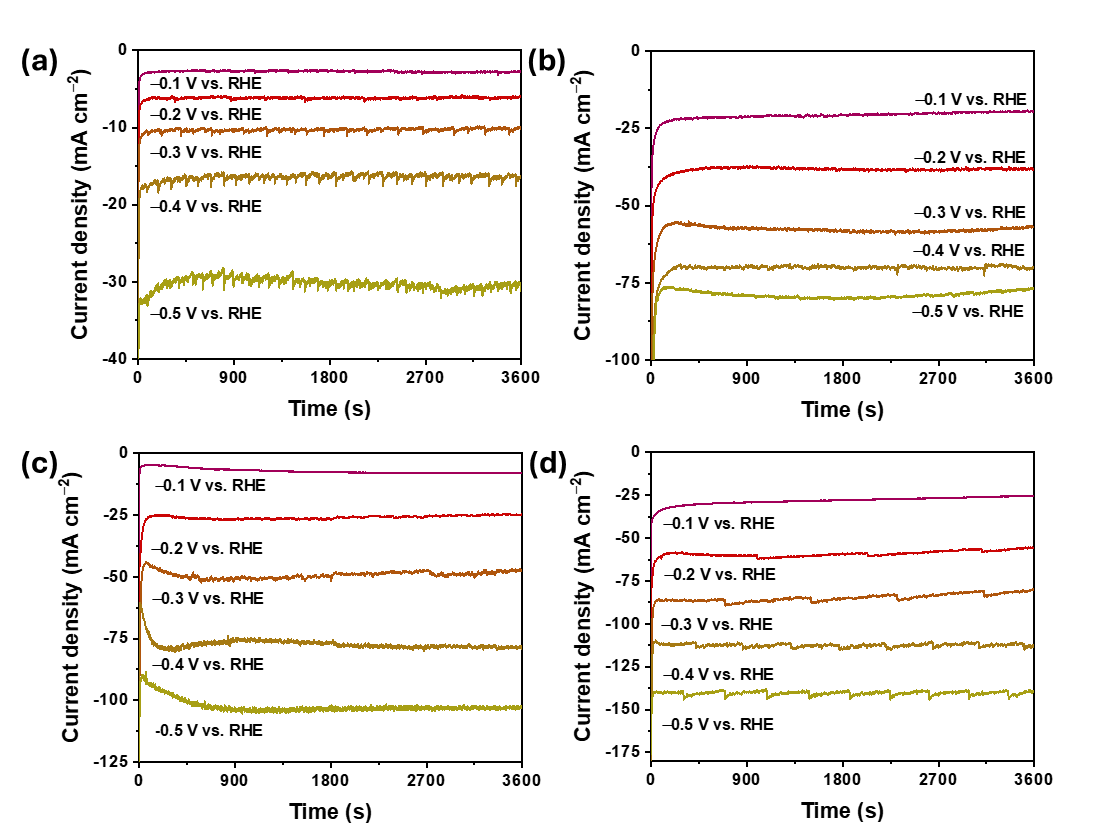


**Figure S23.** One-hour of continuous eNO_3_RR electrolysis profiles of (a) NGO, (b) Cu_2_-HoD/NGO, (c) Co_2_-HoD/NGO, and (d) CoCu-HeD/NGO at different potentials.


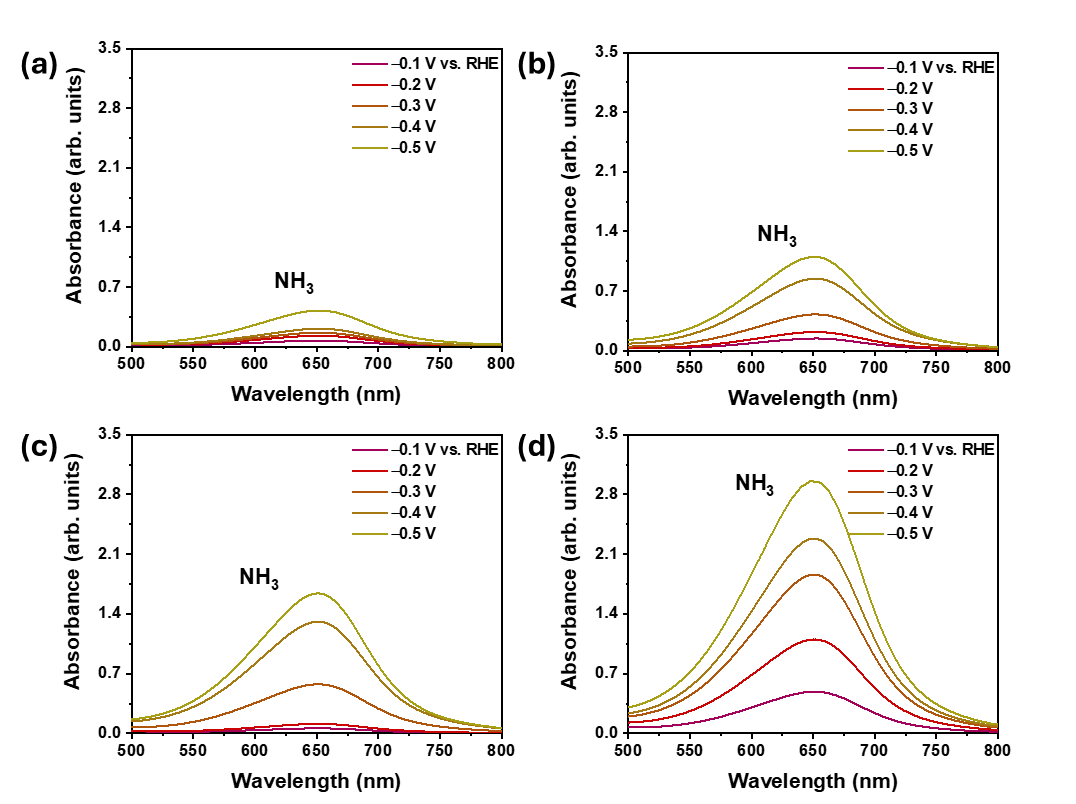


**Figure S24.** UV-visible absorbance spectra of NH_3_ produced during eNO_3_RR electrolysis using (a) NGO, ((b) Cu_2_-HoD/NGO, (c) Co_2_-HoD/NGO, and (d) CoCu-HeD/NGO at different potentials.


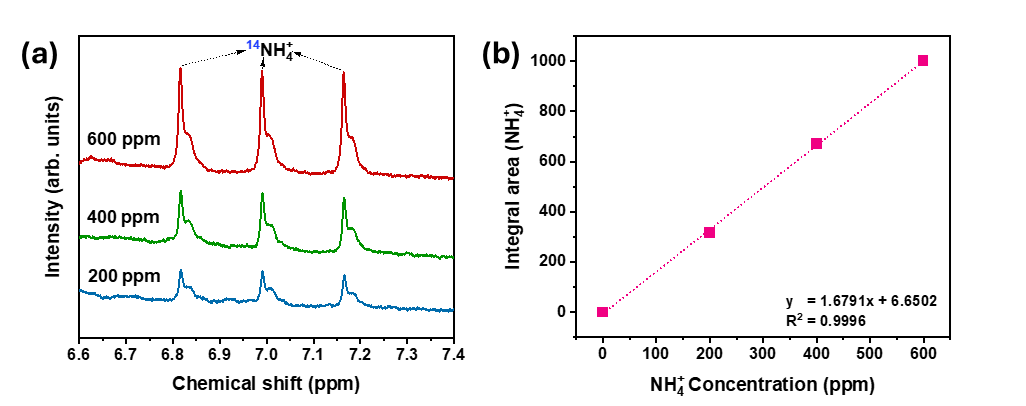


**Figure S25.** NMR calibration curves for determining NH_4_^+^ using ammonium chloride solutions of known concentrations as standards: (a) NMR spectra with different concentration of NH_4_^+^_,_ and (b) concentration-integral area calibration curve for different NH_4_^+^ concentrations.


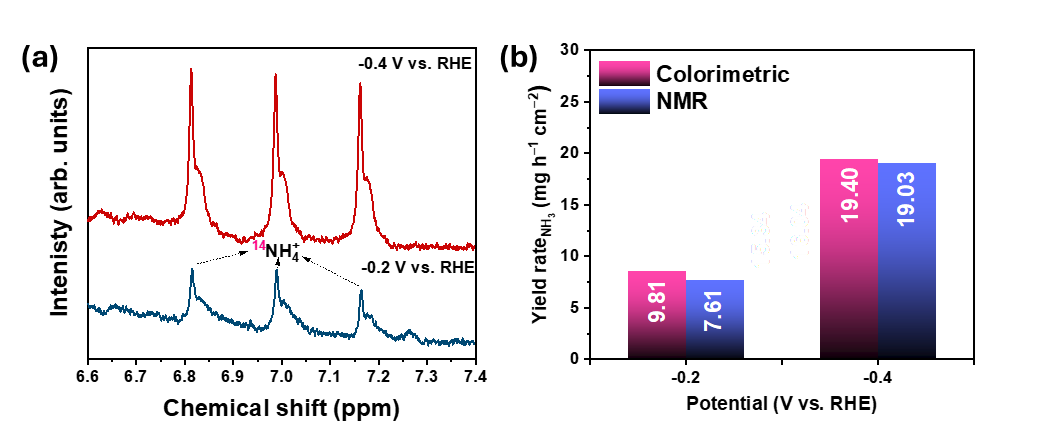


**Figure S26.** (a) The ^1^H NMR spectra for the catholyte after 1 h of eNO_3_RR electrolysis under different potential with CoCu-HD/NGO; and (b) corresponding NH_4_^+^ yield rate comparing with UV-vis results obtained via indophenol blue method.


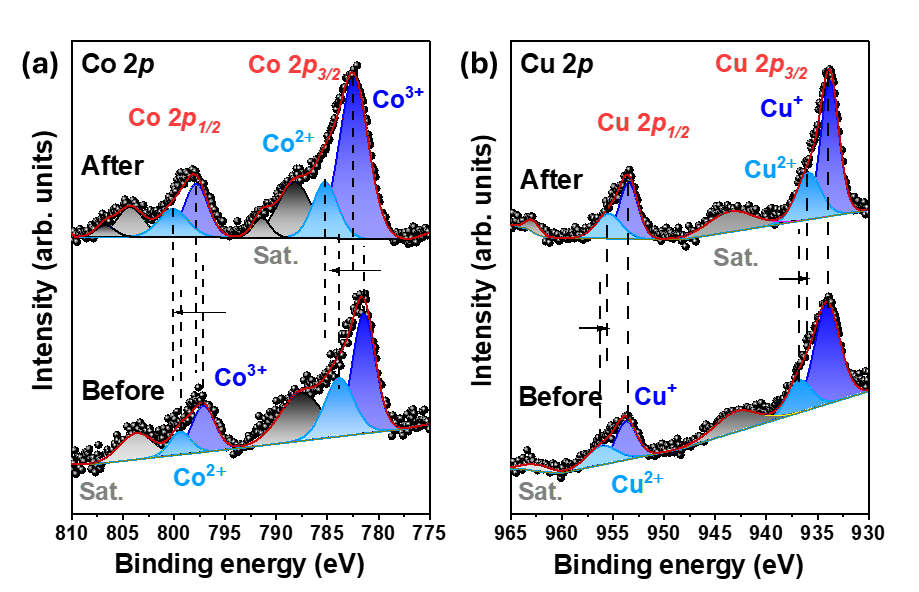


**Figure S27.** Core level XPS spectra of (a) Co 2*p*, and (b) Cu 2*p* for CoCu-HeD/NGO electrode before and after eNO_3_RR cyclic stability for 12 h.


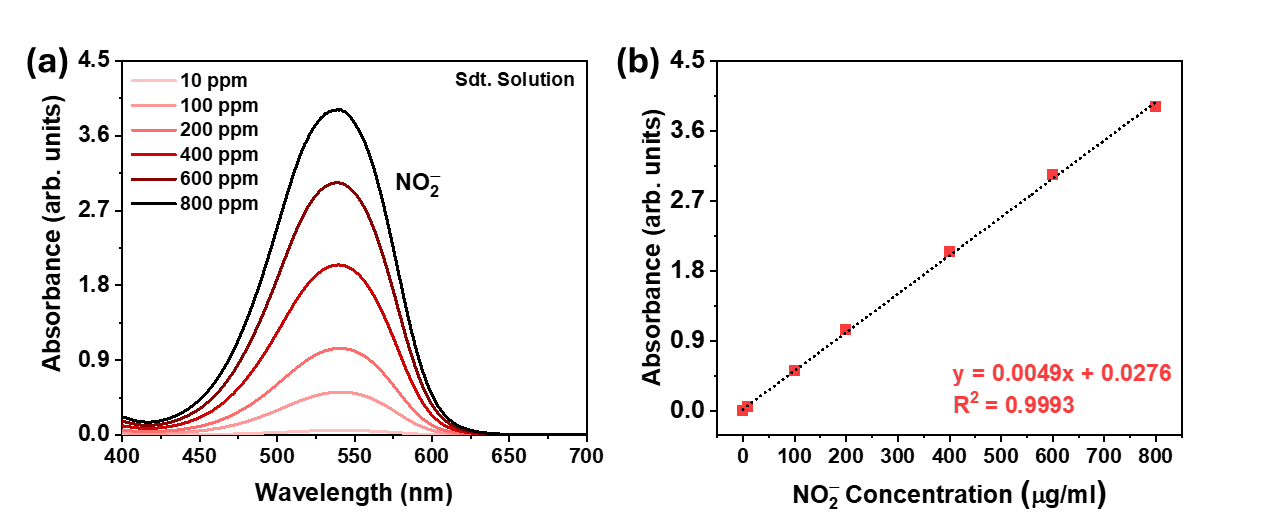


**Figure S28.** UV-vis calibration curves for determining NO_2_^‒^ using KNO_2_ solutions of known concentrations as standards: (a) UV-vis spectra using the indophenol blue method with NO_2_^‒^_,_ and (b) concentration-absorbance calibration curve at 540 nm for different NO_2_^‒^ concentrations.


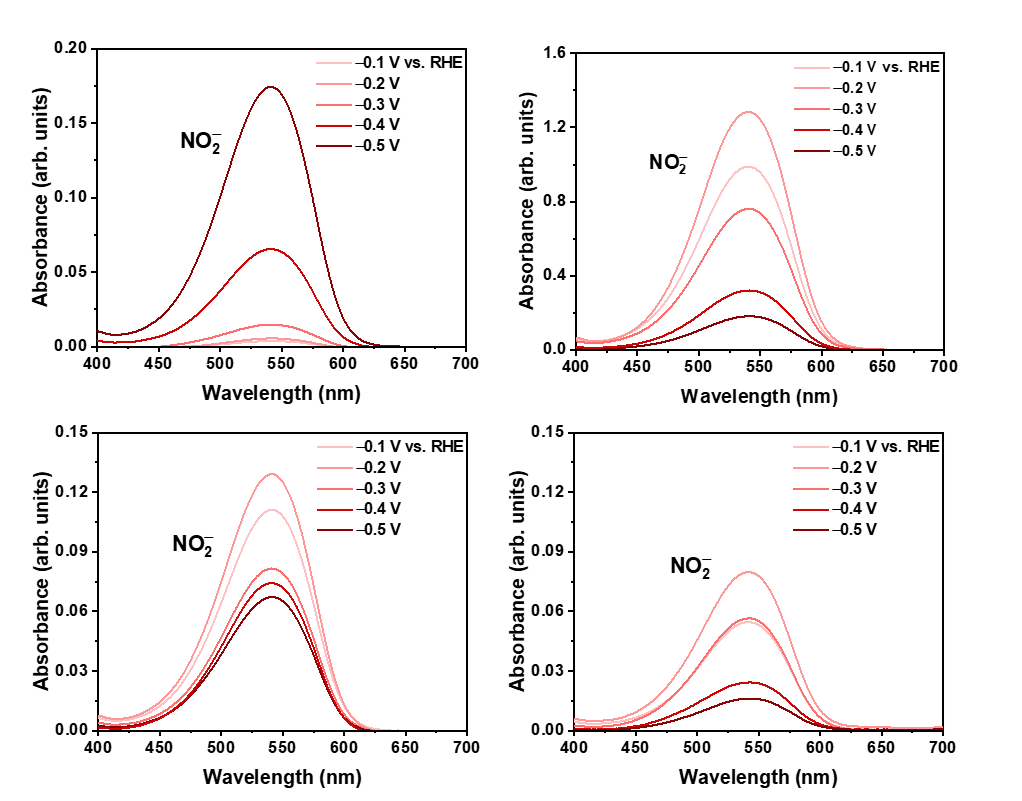


**Figure S29.** UV-visible absorbance spectra of NO_2_^‒^ produced during eNO_3_RR electrolysis using (a) NGO, (b) Cu_2_-HoD/NGO, (c) Co_2_-HoD/NGO, and (d) CoCu-HeD/NGO at different potentials.


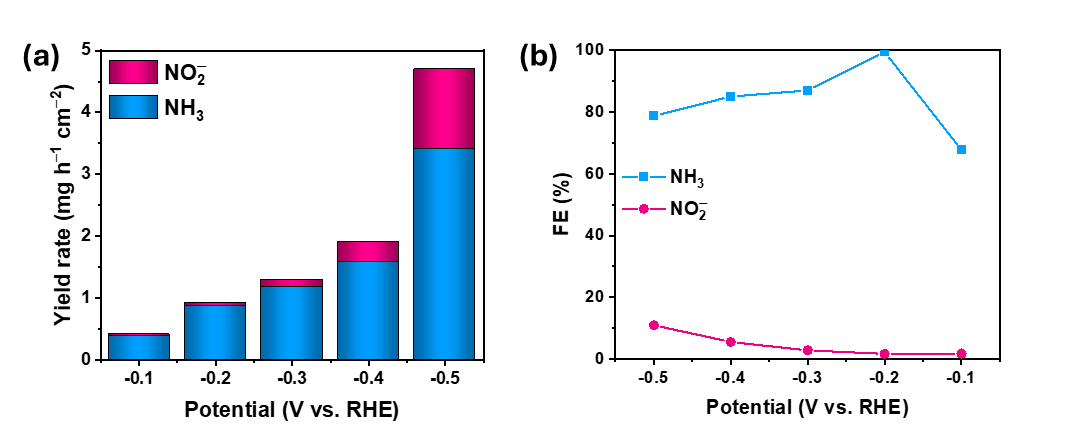


**Figure S30.** Comparison of (a) yield rate and (b) FE for NH_3_ and NO_2_^‒^ during eNO_3_RR using NGO.


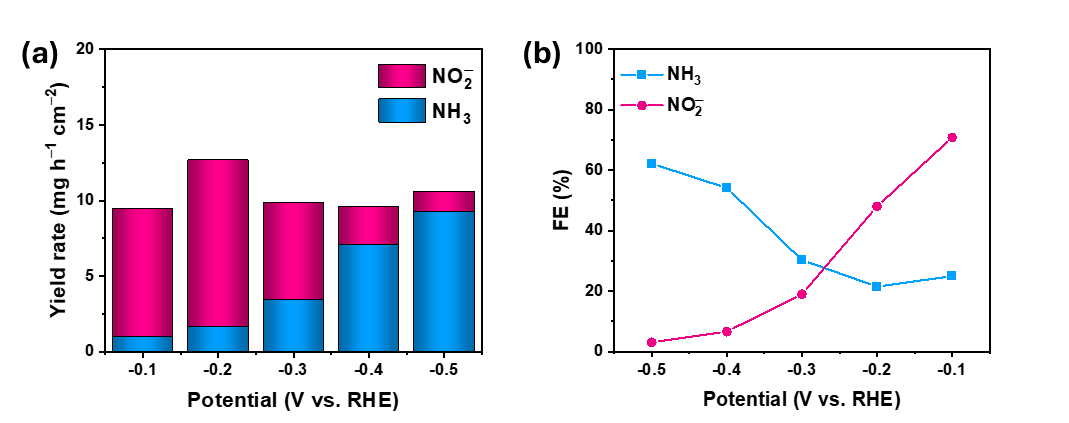


**Figure S31.** Comparison of (a) yield rate and (b) FE for NH_3_ and NO_2_^‒^ during eNO_3_RR using Cu_2_-HoD/NGO.


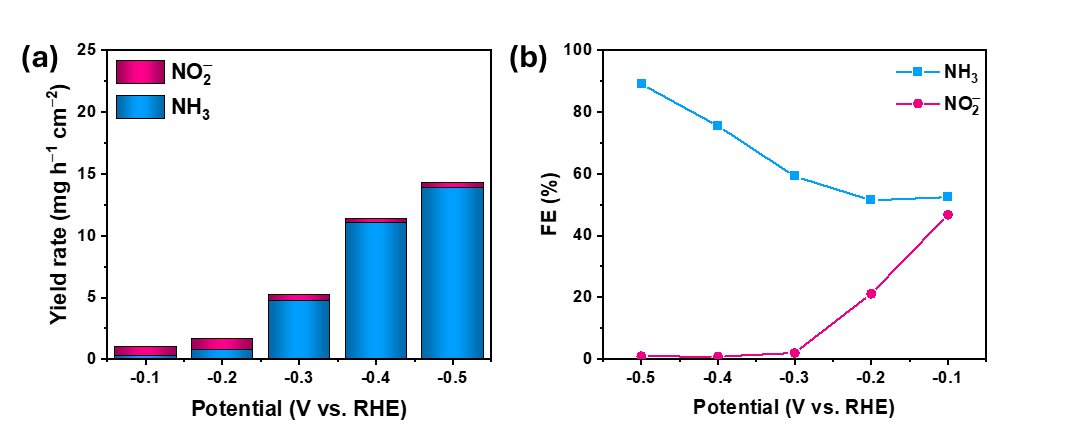


**Figure S32.** Comparison of (a) yield rate and (b) FE for NH_3_ and NO_2_^-^ during eNO_3_RR using Co_2_-HoD/NGO.


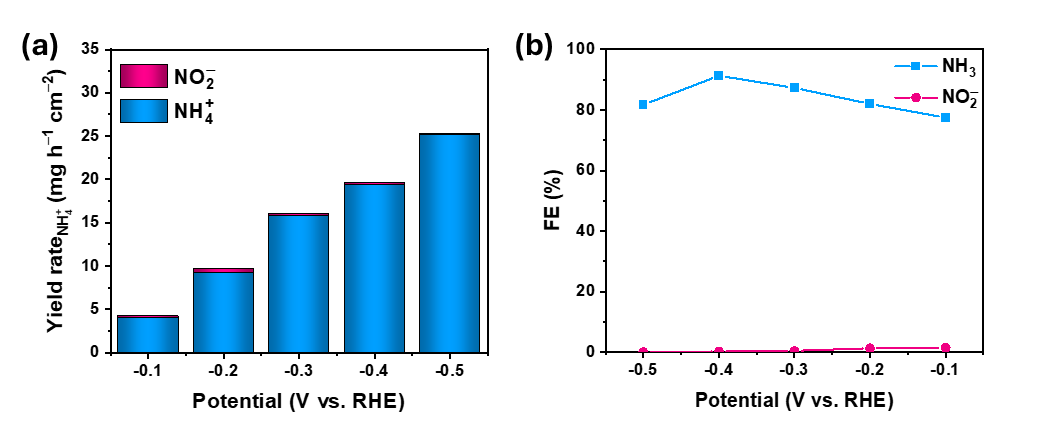


**Figure S33.** Comparison of (a) yield rate and (b) FE for NH_3_ and NO_2_^‒^ during eNO_3_RR using CoCu-HeD/NGO.


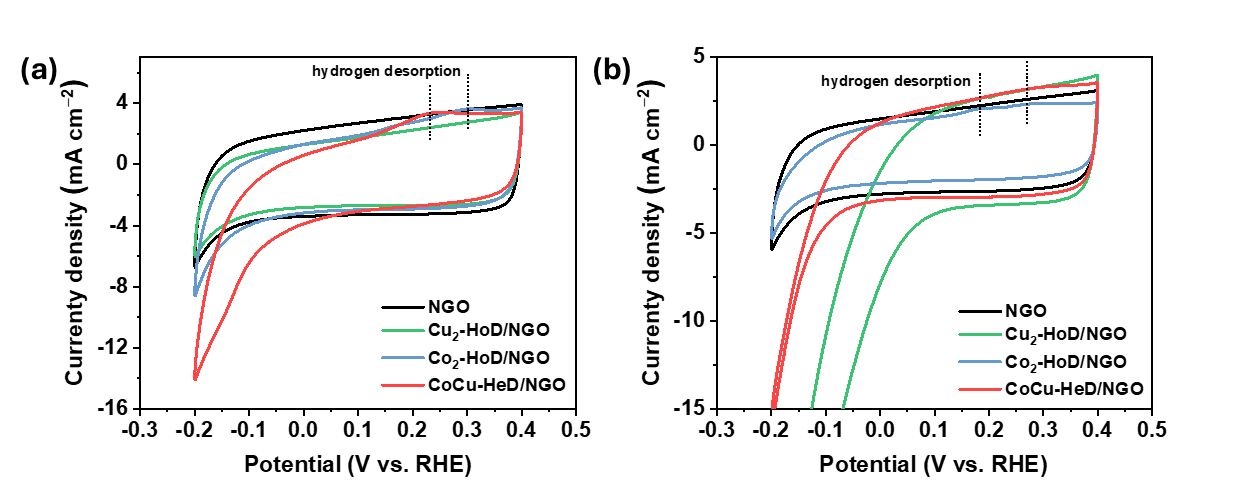


**Figure S34.** CV profiles of the NGO, Cu_2_-HoD/NGO, Co_2_-HoD/NGO, and CoCu-HeD/NGO catalysts with a scan rate of 50 mV s^−1^ in 1.0 M KOH solution: (a) without and (b) with NO_3_^−^ addition.


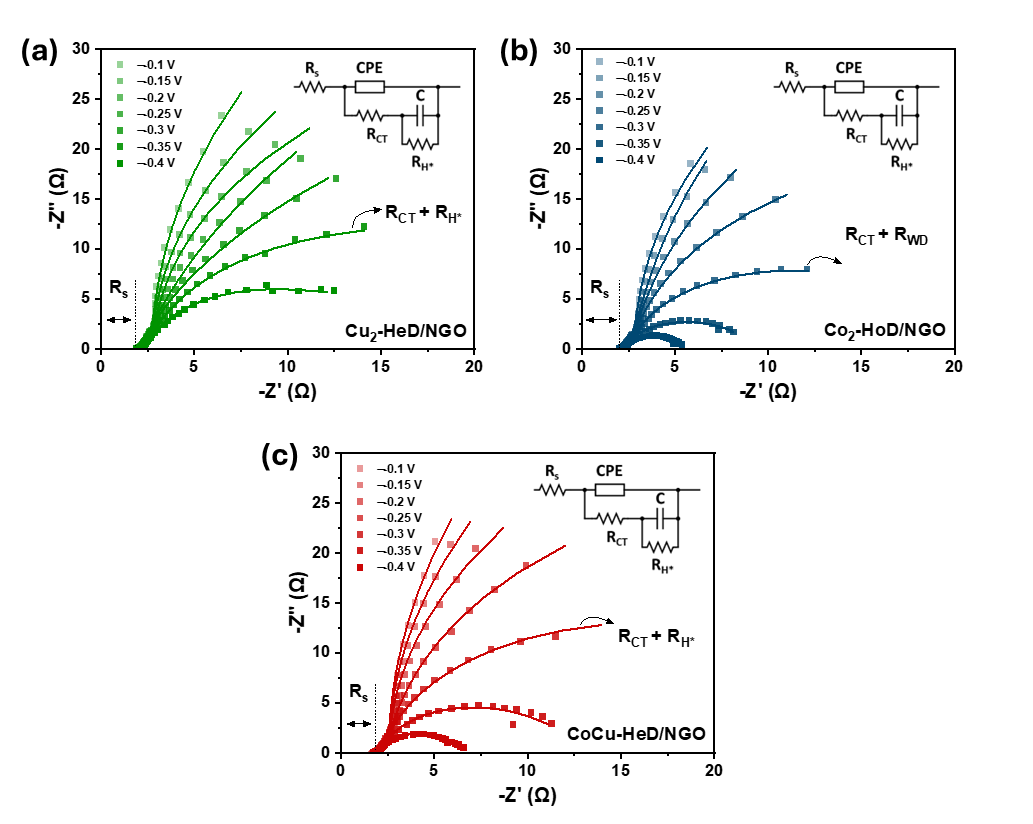


**Figure S35.** Nyquist plots for (a) Cu_2_-HoD/NGO, (b) Co_2_-HoD/NGO, and (c) CoCu-HeD/NGO catalysts at various potentials in 1.0 M KOH solution without NO_3_^−^ addition.


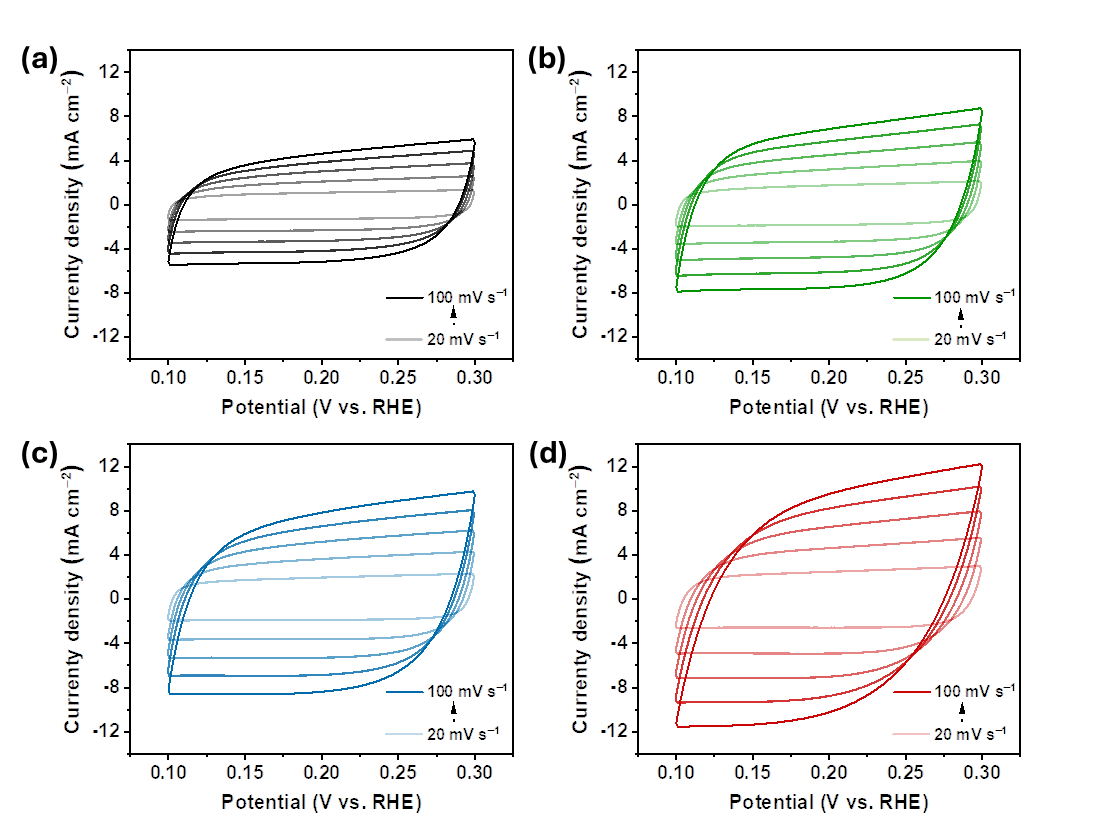


**Figure S36.** CV curves of (a) NGO, (b) Cu_2_-HoD/NGO, (c) Co_2_-HoD/NGO, and (d) CoCu-HeD/NGO catalysts in the non-Faradic regions from 0.1 to 0.3 V vs. RHE at different scan rates from 20-100 mV s^−1^ to estimate C_dl_ values.


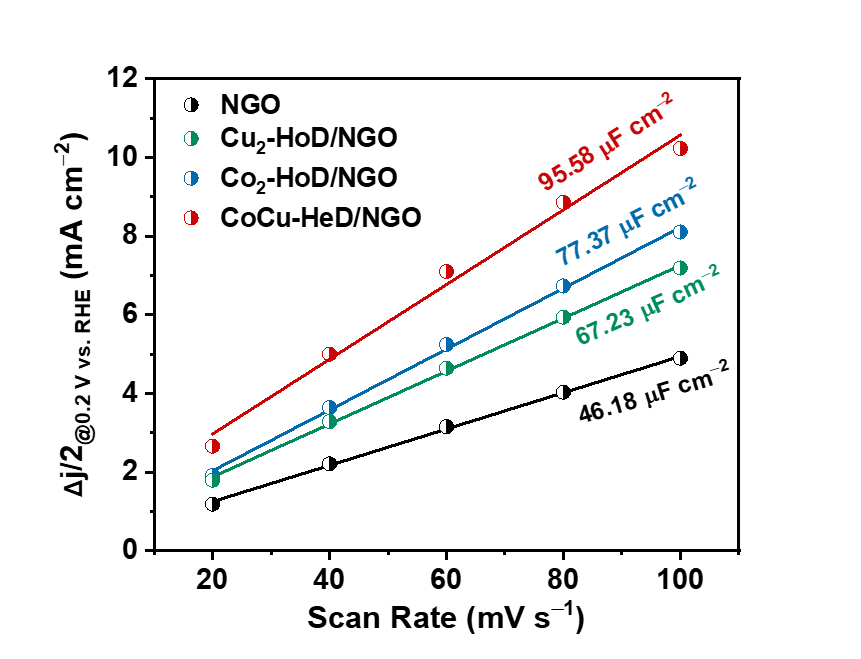


**Figure S37.** Double-layer capacitance (C_dl_) plots for various catalysts calculated at 0.2 V vs. RHE using different scan rate profiles at non-faradic region.


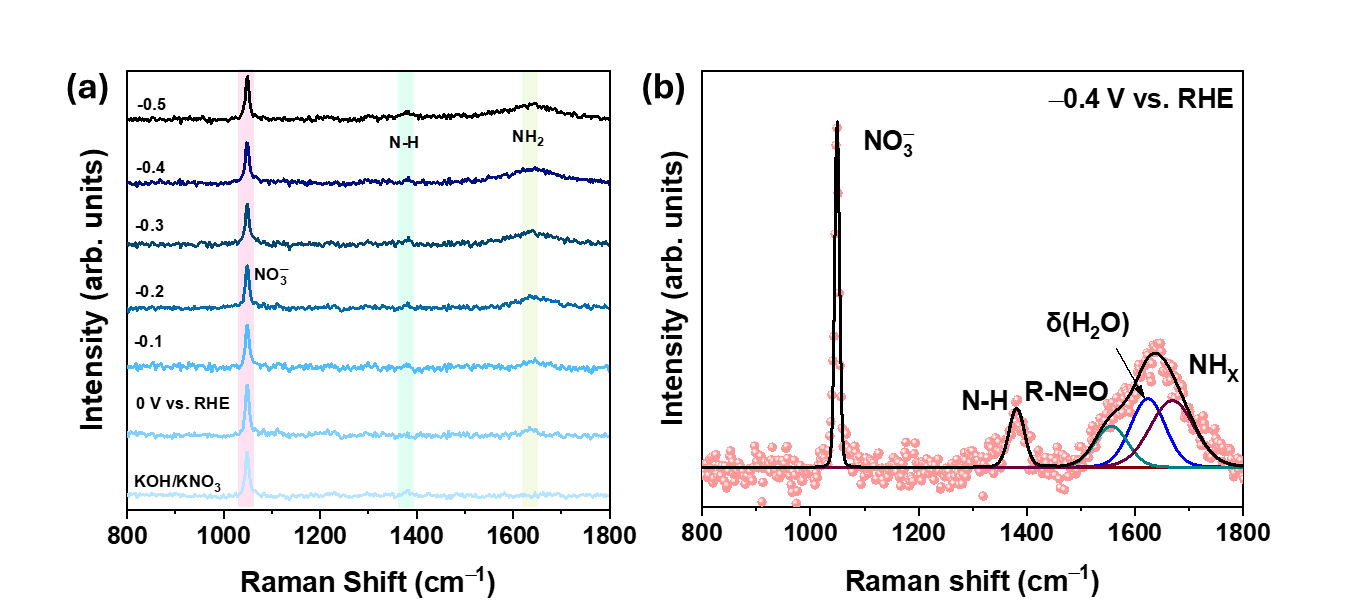


**Figure S38.** (a) In situ Raman spectra in the range of 800 – 1800 cm^−1^ at various potentials from 0 to ‒0.5 V vs. RHE, and (b) deconvoluted Raman spectrum at −0.4 V vs. RHE, highlighting the evolution of vibrational features associated with intermediate species during eNO_3_RR using CoCu-HeD/NGO catalyst.


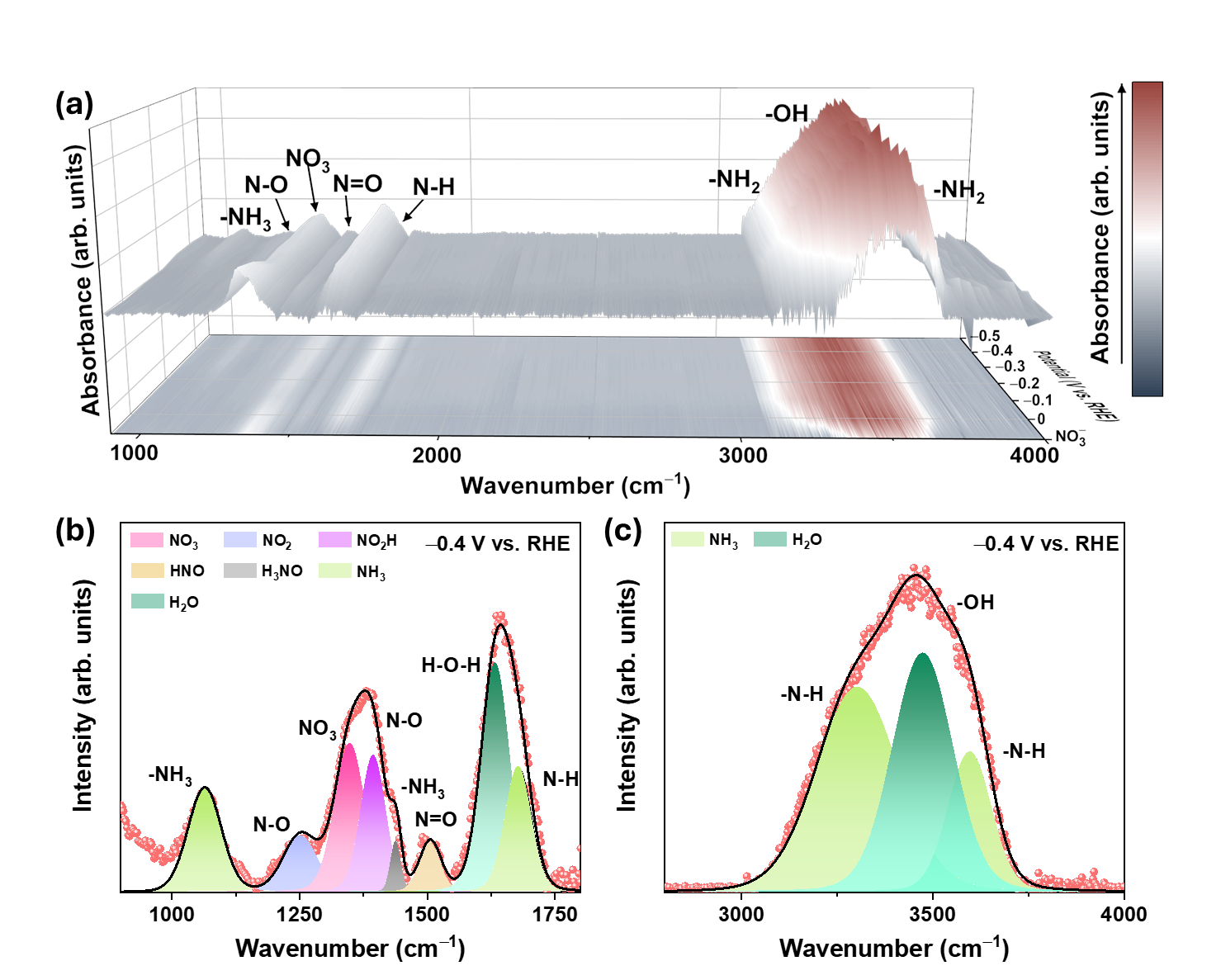


**Figure S39.** (a) Three-dimensional FTIR spectra and corresponding contour plot in the range of 800 – 4000 cm^−1^ for the catholyte obtained after 1 h of eNO_3_RR using CoCu-HeD/NGO at various potentials from 0 to ‒0.5 V vs. RHE, and (b and c) deconvoluted FTIR spectrum at −0.4 V vs. RHE for two different spectral ranges.

**
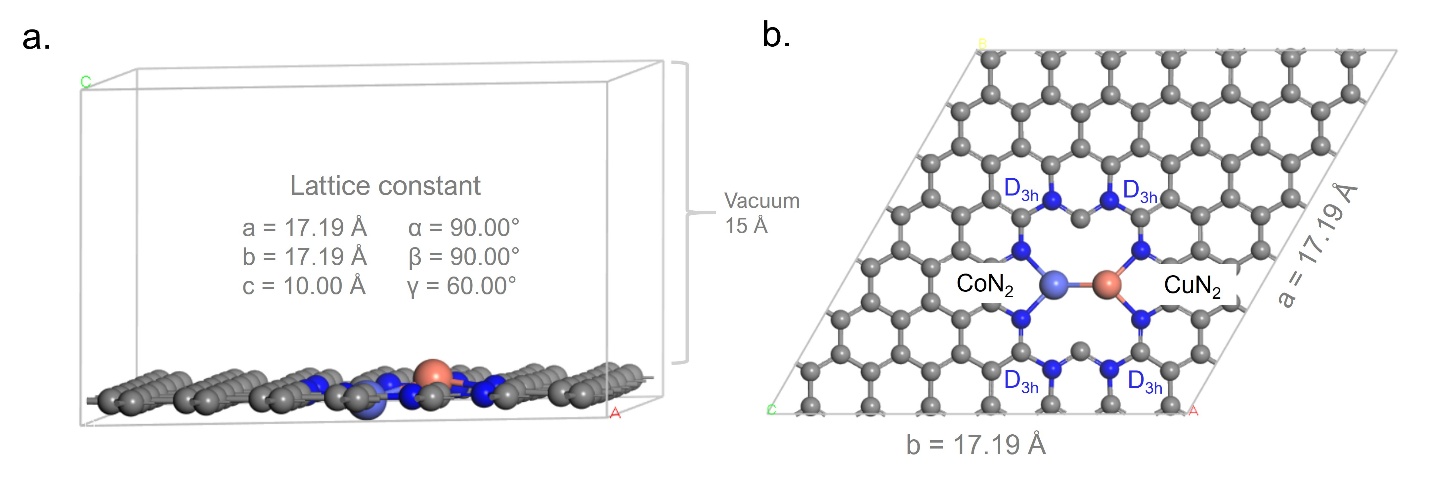
Figure S40.** Schematic representation of the (a) side view with lattice constant, and (b) top view of the CoCu-HeD/NGO catalyst. Atom colors: purple for Co, rose gold for Cu, blue for N, and grey for C.


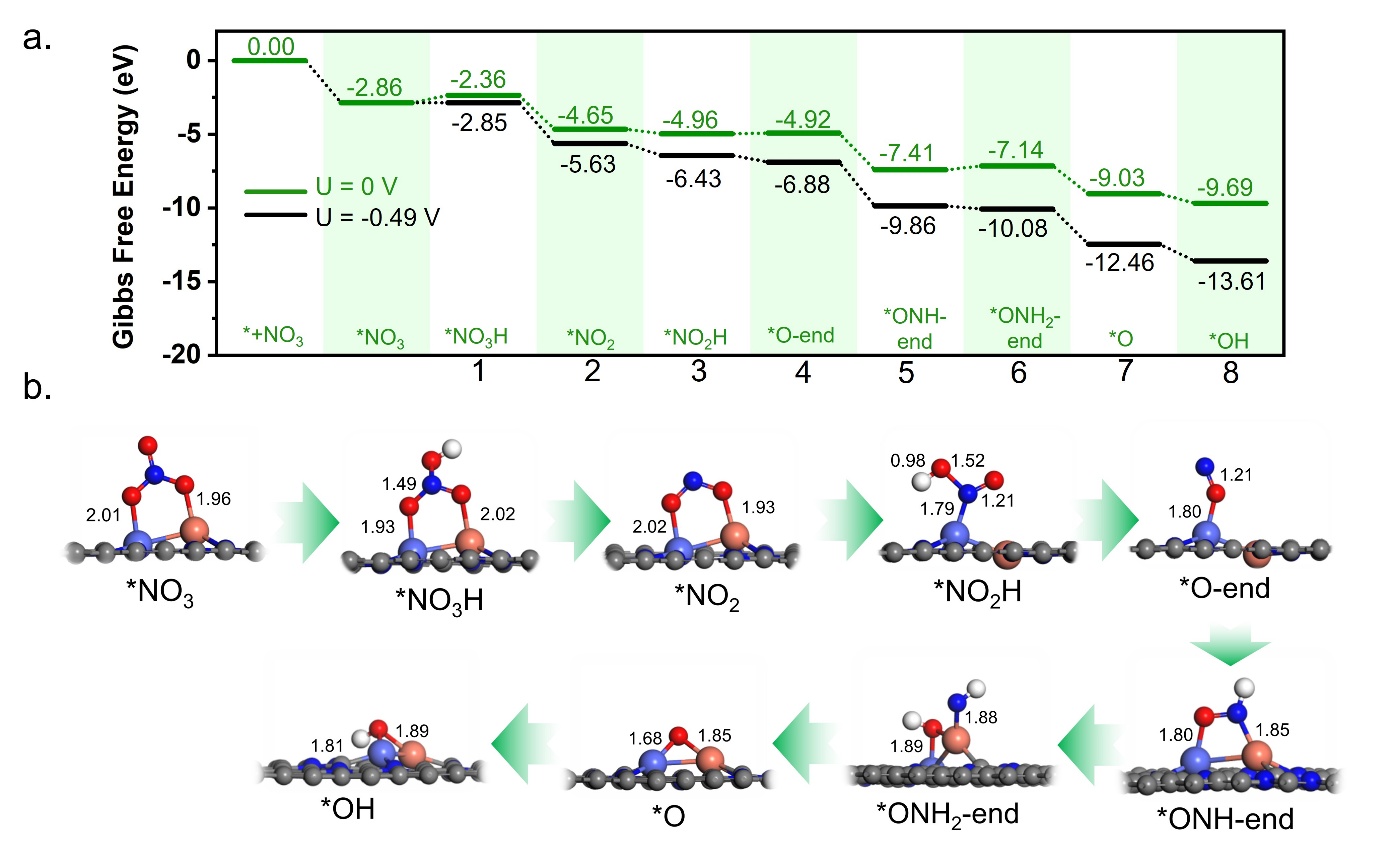


**Figure S41**. (a and b) The Gibbs free energy diagrams for eNO_3_RR on the CoCu-HeD/NGO catalyst through O-end pathway.

**
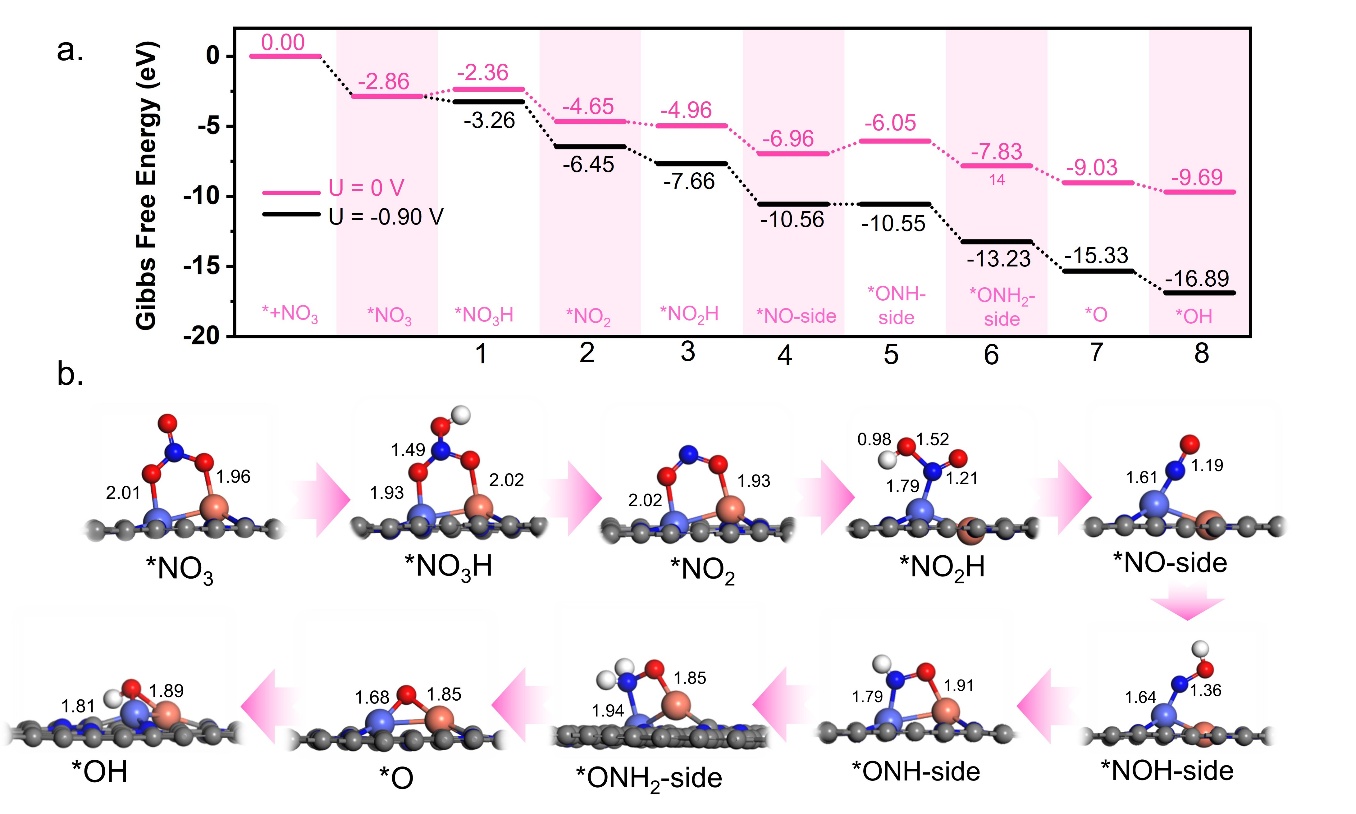
**

**Figure S42**. (a and b) The Gibbs free energy diagrams for eNO_3_RR on the CoCu-HeD/NGO catalyst through ONH-side pathway.


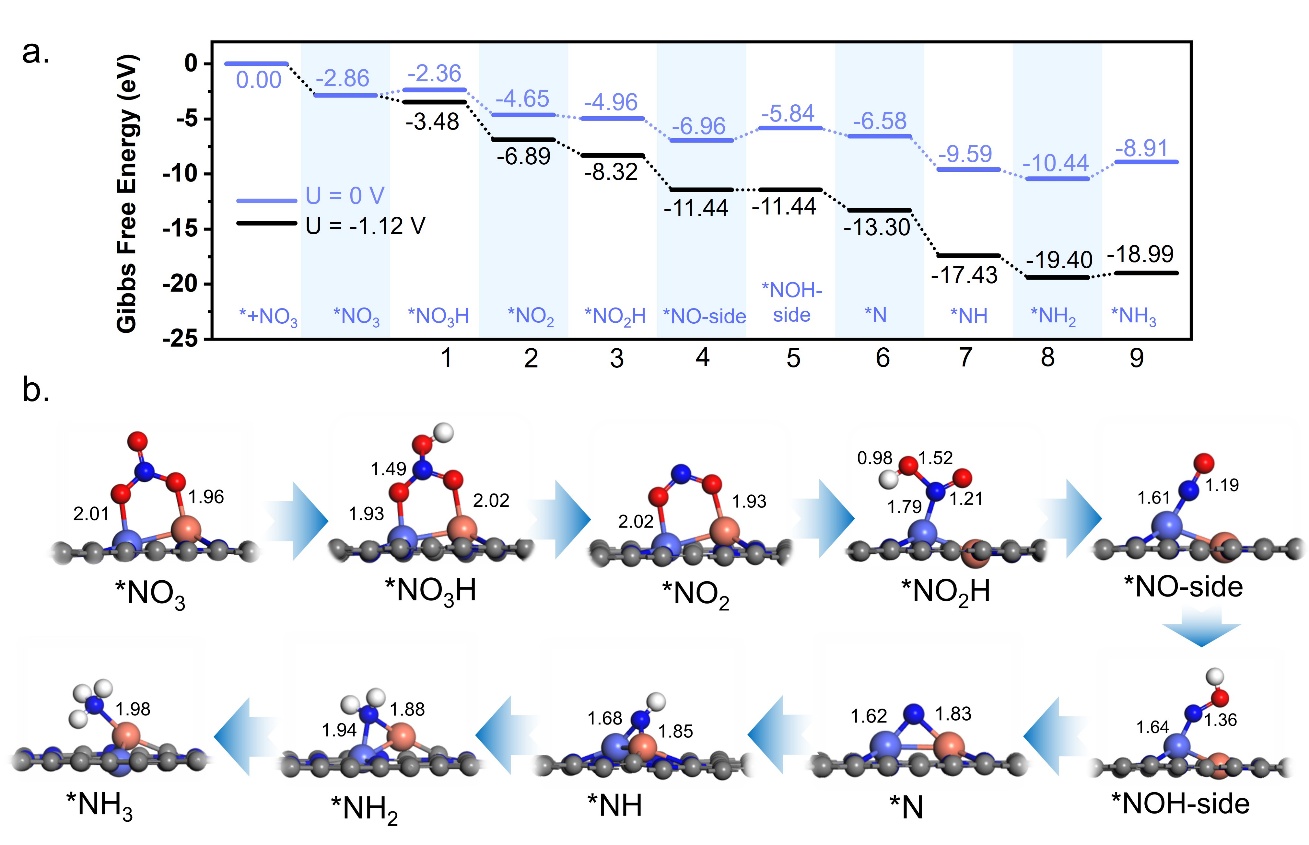


**Figure S43**. (a and b) The Gibbs free energy diagrams for eNO_3_RR on the CoCu-HeD/NGO catalyst through NOH-side pathway.


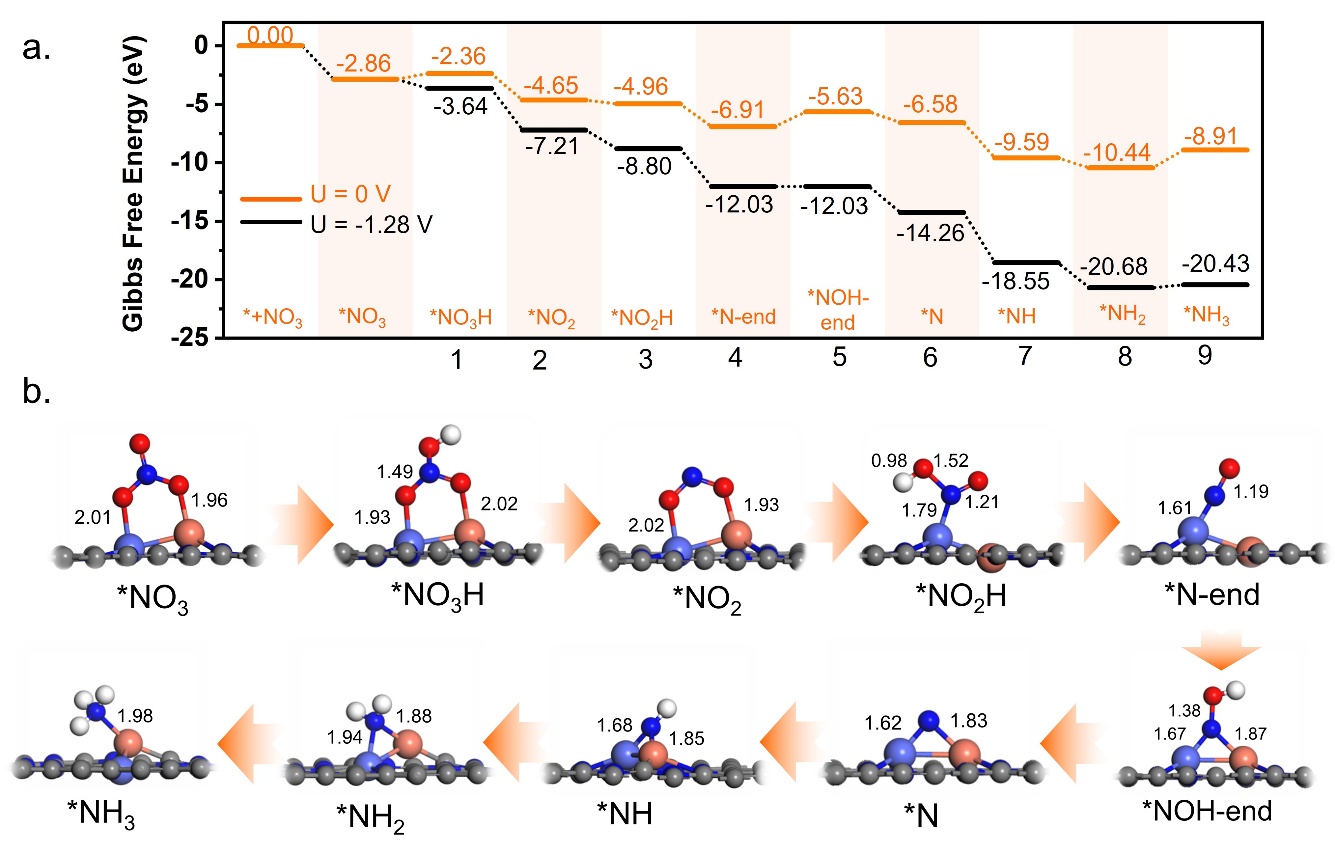


**Figure S44**. (a and b) The Gibbs free energy diagrams for eNO_3_RR on the CoCu-HeD/NGO catalyst through N-end pathway.


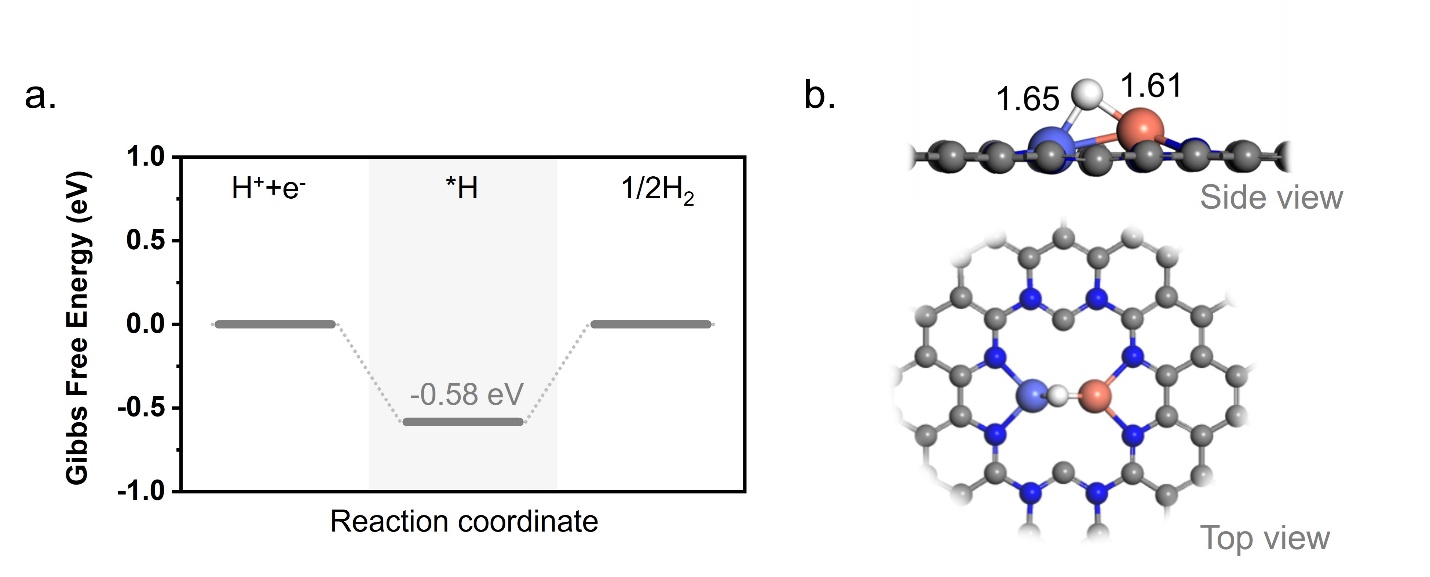


**Figure S45**. (a and b) The Gibbs free energy diagrams for HER on the CoCu-HeD/NGO catalyst through N-end pathway.


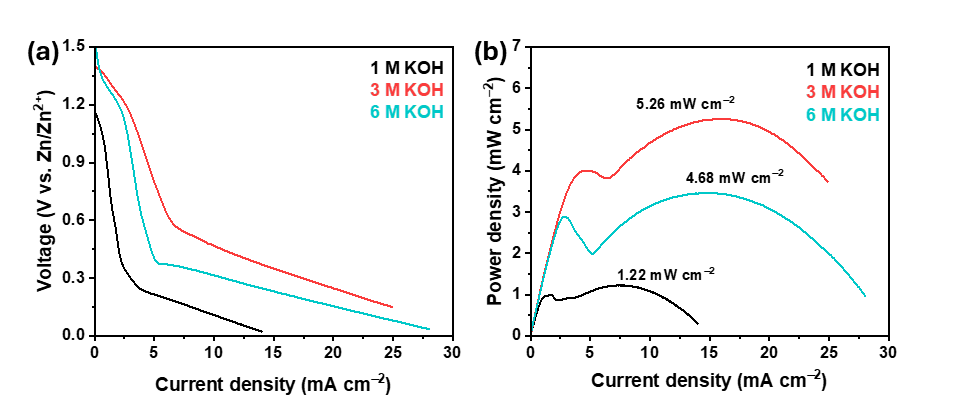


**Figure S46.** (a) Discharge polarization profiles and (b) corresponding power density plot of the assembled Zn-NO_3_^‒^ battery with CoCu-HeD/NGO cathode at different anolyte concentrations (1.0, 3.0, and 6.0 M KOH).


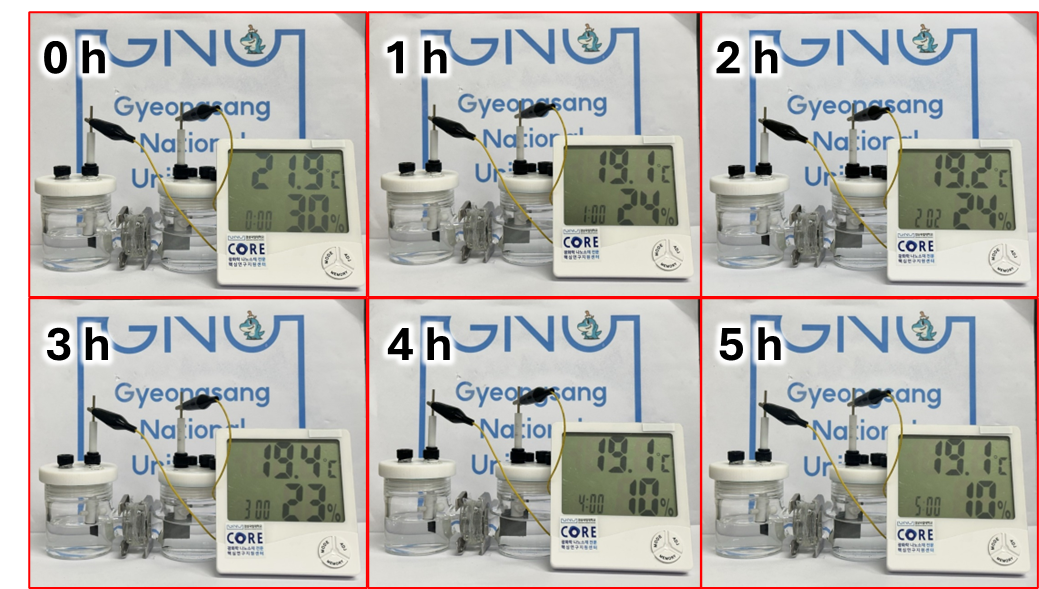


**Figure S47.** Photograph of an electronic timer powered by assembled Zn-NO_3_^‒^ battery with CoCu-HeD/NGO cathode for 5 h.


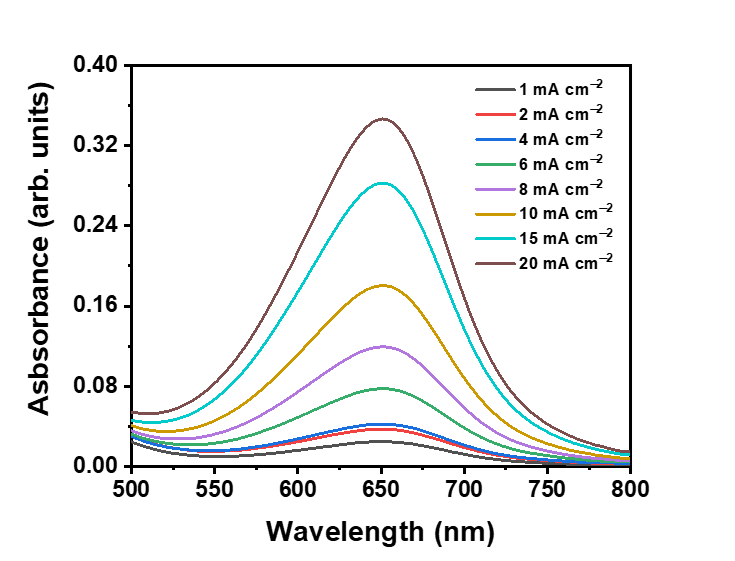


**Figure S48.** UV-vis absorbance spectra of NH_3_ produced during discharging of the Zn-NO_3_^‒^ battery with CoCu-HeD/NGO cathode under various current densities**.**


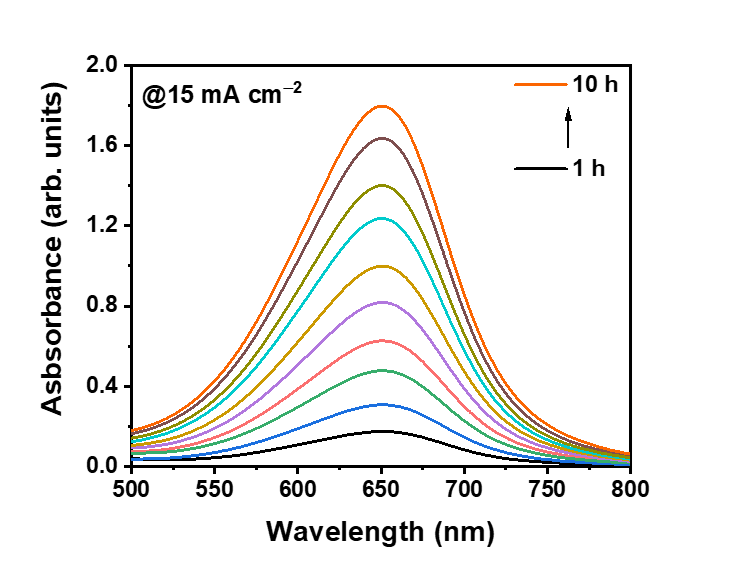


**Figure S49.** Time-dependent UV-vis spectra of NH_3_ produced at fixed time interval during discharging for 10 h of the Zn-NO_3_^‒^ battery with CoCu-HeD/NGO cathode at 15 mA cm^‒2^.


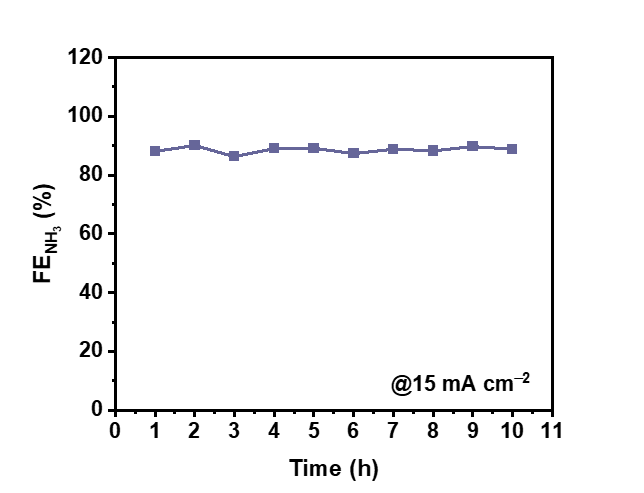


**Figure S50.** Calculated FE% for long-term durability test of the assembled Zn-NO_3_^‒^ battery with CoCu-HeD/NGO cathode at 15 mA cm^‒2^ for 10 h


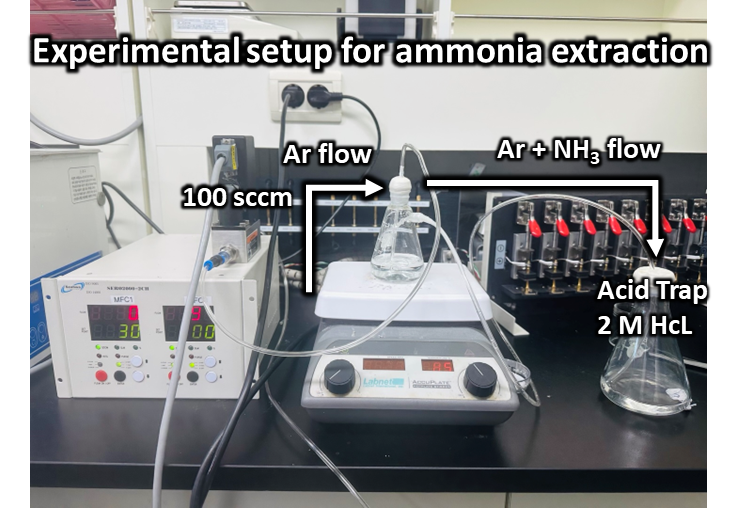


**Figure S51.** Picture represents instrumental setup used for NH_3_ capture via acid trap method.

**Table S1**. Comparison of elemental composition weight percentage of synthesized catalysts obtained from EDS and ICP-OES analyses.

| **Catalyst** | **Element** | **EDS (wt.%)** | **ICP-OES (wt.%)** |
| --- | --- | --- | --- |
| Co_2_-Hod/NGO | Co | 2.95 | - |
| Cu_2_-Hod/NGO | Cu | 3.09 | - |
| CoCu-HeD/NGO | Co | 1.47 | 1.54 |
|  | Cu | 1.82 | 1.65 |

**Table S2.** EXAFS structure parameters for different samples.

| **Catalyst** | ***k*-edge** | **Shell** | **N** | **R (Å)** | **σ^2^/10^−3^Å^2^** | ***R* factor** |
| --- | --- | --- | --- | --- | --- | --- |
| **Fitted with N_2_-M-M-N_2_ dimer configuration** | | | | | | |
| CoCu-HeD/NGO | Co | Co-Cu | 1 | 2.324 | 0.01332 | 0.0133 |
|  |  | Co-N | 2 | 1.999 | 0.02241 |  |
|  | Cu | Cu-Co | 1 | 2.306 | 0.00612 | 0.0220 |
|  |  | Cu-N | 2 | 1.955 | 0.00902 |  |
| Co_2_-HeD/NGO | Co | Co-Co | 1 | 2.279 | 0.01593 | 0.0114 |
|  |  | Co-N | 4 | 2.001 | 0.02693 |  |
| Cu_2_-HeD/NGO | Cu | Cu-Cu | 1 | 2.331 | 0.00664 | 0.0088 |
|  |  | Cu-N | 4 | 1.953 | 0.00834 |  |

**N** is the coordination number; **R** is interatomic distance between two atoms; **σ^2^** is Debye-Waller factor; ***R* factor** is used to value the goodness of the fitting.

**Table S3.** EIS circuit fitting parameters for all catalysts.

| **CoCu-HeD/NGO** | | | | | | | | | | | | |
| --- | --- | --- | --- | --- | --- | --- | --- | --- | --- | --- | --- | --- |
| **Potential** | **R_S_** | | **CPE** | | **n** | | **R_ct_** | | **C** | | **R_H_** | |
| 100 | 1.846 | | 0.00527 | | 0.5 | | 0.6283 | | 0.004619 | | 588.2 | |
| 150 | 1.838 | | 0.01134 | | 0.5 | | 0.5386 | | 0.004677 | | 248.2 | |
| 200 | 1.836 | | 0.02073 | | 0.5 | | 0.4829 | | 0.005233 | | 118.9 | |
| 250 | 1.826 | | 0.02891 | | 0.5 | | 0.4585 | | 0.005663 | | 55.73 | |
| 300 | 1.779 | | 0.03354 | | 0.5 | | 0.4266 | | 0.005678 | | 20.12 | |
| 350 | 1.806 | | 0.03601 | | 0.5 | | 0.4353 | | 0.005726 | | 13.13 | |
| 400 | 1.829 | | 0.04258 | | 0.5 | | 0.2925 | | 0.005734 | | 5.079 | |
| **Co_2_-HoD/NGO** | | | | | | | | | | | | |
| **Potential** | | **R_S_** | | **CPE** | | **n** | | **R_ct_** | | **C** | | **R_H_** |
| 100 | | 2.073 | | 0.02631 | | 0.5 | | 0.3916 | | 0.002618 | | 257.3 |
| 150 | | 2.077 | | 0.03191 | | 0.5 | | 0.3755 | | 0.002759 | | 115.2 |
| 200 | | 2.083 | | 0.03808 | | 0.5 | | 0.3792 | | 0.003682 | | 67.69 |
| 250 | | 2.092 | | 0.05017 | | 0.5 | | 0.3346 | | 0.004287 | | 32.28 |
| 300 | | 2.113 | | 0.05659 | | 0.5 | | 0.3828 | | 0.005027 | | 11.02 |
| 350 | | 2.2 | | 0.05837 | | 0.5 | | 0.3026 | | 0.005121 | | 5.691 |
| 400 | | 2.143 | | 0.05691 | | 0.5 | | 0.2847 | | 0.005416 | | 3.534 |
| **Cu_2_-HoD/NGO** | | | | | | | | | | | | |
| **Potential** | | **R_S_** | | **CPE** | | **n** | | **R_ct_** | | **C** | | **R_H_** |
| 100 | | 1.951 | | 0.02176 | | 0.5 | | 0.3878 | | 0.004452 | | 311.1 |
| 150 | | 1.963 | | 0.02646 | | 0.5 | | 0.3741 | | 0.004381 | | 122.7 |
| 200 | | 1.972 | | 0.03145 | | 0.5 | | 0.3767 | | 0.004103 | | 73.2 |
| 250 | | 1.975 | | 0.03691 | | 0.5 | | 0.3869 | | 0.003739 | | 49.82 |
| 300 | | 1.984 | | 0.04563 | | 0.5 | | 0.4019 | | 0.003201 | | 31.47 |
| 350 | | 2.006 | | 0.04913 | | 0.5 | | 0.4089 | | 0.002579 | | 20.76 |
| 400 | | 2.034 | | 0.05022 | | 0.5 | | 0.3438 | | 0.002603 | | 15.27 |

**R_S_** is solution resistance; **CPE** is constant phase element; **R_CT_** is charge transfer resistance; C is pseudo-capacitance; and **R_H_** is resistance associated with H* behavior**.**

**Table S4. Comparison table for the eNO_3_RR performance.**

| Catalyst | Max. FE (%) | Max. Yield rate (mg h**^−^**^1^ cm**^−^**^2^) | Corresponding potential (‒V vs. RHE) | Nitrate concentration | Ref |
| --- | --- | --- | --- | --- | --- |
| CoCu-HeD/NGO | 96.31 | 19.40 | 0.4 | 100 mM | This work |
| Co_2_-HoD/NGO | 75.49 | 11.03 |  |  |  |
| Cu_2_-HoD/NGO | 54.14 | 7.04 |  |  |  |
| V-Cu NAE | 95.9 | 7.8 | 0.3 | 200 mM | [11] |
| Cu/Ni–NC | 98.5 | 5.5 | 0.7 | 100 ppm | [12] |
| Cu-Cu2O/Ni2P | 96.4 | 14.6 | 1.0 | 100 mM | [13] |
| RuCu DAs/NGA | 95.7 | 3.10 | 0.4 | 100 mM | [14] |
| Cu-N-C SAC | 84.7 | 4.5 | 1.0 | 100 mM | [15] |
| CoCu-NC DAC | 95.3 | 2.41 | 0.6 | 200 mM | [16] |
| CuNi DSACs | 94.1 | 18.30 | 0.4 | 100 mM | [17] |
| Cu_2_/N_3_−6 | 97.4 | 18.2 | 0.8 | 100 mM | [18] |
| Fe@Cu1FeOx | 95.4 | 1.98 | 1.3 V vs. SCE | 1000 ppm | [19] |
| Fe-BCN | 97.48 | 2.06 | 0.3 | 500 mM | [20] |
| CuNiFe–LDH HNCs | 90.1 | 3.58 | 1.2 | 50 mM | [21] |
| Pt_0.8_Fe_0.2_Co_0.2_Ni_0.2_Cu_0.2_ | 98.80 | 11.05 | 3 | 100 mM | [22] |
| Cu-*cis*-N_2_O_2_ SAC | 70 | 28.73 | 2.0 | 1000 ppm | [23] |
| CoCu-NC DAC | 95.3 | 2.41 | 0.6 | - | [24] |
| Cu-CoP | 85.1 | 7.65 | 1.0 | 10mM | [25] |
| CuCo-SNC | 97.8 | 14.99 | 0.3 | 0.1 M | [26] |

**References**

[1] P. Hohenberg, W. Kohn, Inhomogeneous Electron Gas, Physical Review, 136 (1964) B864-B871.

[2] G. Kresse, J. Furthmüller, Efficient iterative schemes for ab initio total-energy calculations using a plane-wave basis set, Physical Review B, 54 (1996) 11169-11186.

[3] J.P. Perdew, J.A. Chevary, S.H. Vosko, K.A. Jackson, M.R. Pederson, D.J. Singh, C. Fiolhais, Atoms, molecules, solids, and surfaces: Applications of the generalized gradient approximation for exchange and correlation, Physical review B, 46 (1992) 6671.

[4] J. Klimeš, D.R. Bowler, A. Michaelides, Van der Waals density functionals applied to solids, Physical Review B, 83 (2011) 195131.

[5] K. Lee, É.D. Murray, L. Kong, B.I. Lundqvist, D.C. Langreth, Higher-accuracy van der Waals density functional, Physical Review B, 82 (2010) 081101.

[6] J.K. Nørskov, J. Rossmeisl, A. Logadottir, L. Lindqvist, J.R. Kitchin, T. Bligaard, H. Jonsson, Origin of the overpotential for oxygen reduction at a fuel-cell cathode, The Journal of Physical Chemistry B, 108 (2004) 17886-17892.

[7] J.H. Montoya, C. Tsai, A. Vojvodic, J.K. Nørskov, The challenge of electrochemical ammonia synthesis: a new perspective on the role of nitrogen scaling relations, ChemSusChem, 8 (2015) 2180-2186.

[8] A. Kulkarni, S. Siahrostami, A. Patel, J.K. Nørskov, Understanding catalytic activity trends in the oxygen reduction reaction, Chemical reviews, 118 (2018) 2302-2312.

[9] F. Calle-Vallejo, M. Huang, J.B. Henry, M.T. Koper, A.S. Bandarenka, Theoretical design and experimental implementation of Ag/Au electrodes for the electrochemical reduction of nitrate, Physical chemistry chemical physics, 15 (2013) 3196-3202.

[10] J.-H. Liu, L.-M. Yang, E. Ganz, Electrocatalytic reduction of CO2 by two-dimensional transition metal porphyrin sheets, Journal of Materials Chemistry A, 7 (2019) 11944-11952.

[11] B. Zhang, Z. Dai, Y. Chen, M. Cheng, H. Zhang, P. Feng, B. Ke, Y. Zhang, G. Zhang, Defect-induced triple synergistic modulation in copper for superior electrochemical ammonia production across broad nitrate concentrations, Nature Communications, 15 (2024) 2816.

[12] Y. Wang, H. Yin, F. Dong, X. Zhao, Y. Qu, L. Wang, Y. Peng, D. Wang, W. Fang, J. Li, N‐coordinated Cu–Ni dual‐single‐atom catalyst for highly selective electrocatalytic reduction of nitrate to ammonia, Small, 19 (2023) 2207695.

[13] H. Zhao, P. Liu, X. Cheng, C. Fan, J. Liu, D. Kan, Y.Q. Wang, A Cu‐Cu2O/Ni2P Heterostructure for Efficient Tandem Catalysis of Electrosynthesis of Ammonia from Nitrate Reduction Reaction in Neutral Medium, Advanced Functional Materials, 2425459.

[14] K. Liu, Z. Sun, X. Peng, X. Liu, X. Zhang, B. Zhou, K. Yu, Z. Chen, Q. Zhou, F. Zhang, Tailoring asymmetric RuCu dual-atom electrocatalyst toward ammonia synthesis from nitrate, Nature Communications, 16 (2025) 2167.

[15] J. Yang, H. Qi, A. Li, X. Liu, X. Yang, S. Zhang, Q. Zhao, Q. Jiang, Y. Su, L. Zhang, Potential-driven restructuring of Cu single atoms to nanoparticles for boosting the electrochemical reduction of nitrate to ammonia, Journal of the American Chemical Society, 144 (2022) 12062-12071.

[16] J. Wei, H. Lin, Y. Li, Y. Guo, S. Liu, M. Sun, Y.-y. Li, Cobalt-copper dual-atom catalyst boosts electrocatalytic nitrate reduction from water, Journal of Hazardous Materials, (2025) 138264.

[17] Y. Hu, H. Lan, J. He, W. Fang, W.-D. Zhang, S. Lu, F. Duan, M. Du, Entropy-Engineered Middle-In Synthesis of Dual Single-Atom Compounds for Nitrate Reduction Reaction, ACS nano, 18 (2024) 23168-23180.

[18] T. Zhao, K. Chen, X. Xu, X. Li, X. Zhao, Q. Cai, K. Chu, J. Zhao, Homonuclear dual-atom catalysts embedded on N-doped graphene for highly efficient nitrate reduction to ammonia: from theoretical prediction to experimental validation, Applied Catalysis B: Environmental, 339 (2023) 123156.

[19] B. Zhou, L. Yu, W. Zhang, X. Liu, H. Zhang, J. Cheng, Z. Chen, H. Zhang, M. Li, Y. Shi, Cu1− Fe Dual Sites for Superior Neutral Ammonia Electrosynthesis from Nitrate, Angewandte Chemie International Edition, 63 (2024) e202406046.

[20] X. Lu, J. Wei, H. Lin, Y. Li, Y.-y. Li, Boron regulated Fe single-atom structures for electrocatalytic nitrate reduction to ammonia, ACS Applied Nano Materials, 7 (2024) 14654-14664.

[21] Y. Fu, Y. Li, F. Fan, B. Chen, X. Hou, Y. Li, H. Li, Y. Fu, W. Qi, Atomic-Level Dispersed Cu in NiFe–LDH Hollow Nanocages for Highly Efficient Electrochemical Nitrate Reduction Reaction, ACS Catalysis, 15 (2025) 6918-6928.

[22] G. Zhu, W. Bao, M. Xie, C. Qi, F. Xu, Y. Jiang, B. Chen, Y. Fan, B. Liu, L. Wang, Accelerating Tandem Electroreduction of Nitrate to Ammonia via Multi‐Site Synergy in Mesoporous Carbon‐Supported High‐Entropy Intermetallics, Advanced Materials, 37 (2025) 2413560.

[23] X.F. Cheng, J.H. He, H.Q. Ji, H.Y. Zhang, Q. Cao, W.J. Sun, C.L. Yan, J.M. Lu, Coordination symmetry breaking of single‐atom catalysts for robust and efficient nitrate electroreduction to ammonia, Advanced Materials, 34 (2022) 2205767.

[24] J. Wei, H. Lin, Y. Li, Y. Guo, S. Liu, M. Sun, Y.-y. Li, Cobalt-copper dual-atom catalyst boosts electrocatalytic nitrate reduction from water, Journal of Hazardous Materials, 493 (2025) 138264.

[25] W. Yang, Z. Chang, X. Yu, P. Wu, R. Shen, L. Wang, X. Cui, J. Shi, Cu‐Co Dual Sites Tandem Synergistic Effect Boosting Neutral Low Concentration Nitrate Electroreduction to Ammonia, Advanced Science, 12 (2025) 2416386.

[26] W. Chen, Z. Sun, S. Zhen, Y. Wang, J. Sun, M. Liu, W.J. Han, L. Lai, W. Wei, L. Zhang, Tuning Asymmetric S‐Bridged Cu─ Co Dual Sites at Atomic‐Level for Efficient Ammonia Electrosynthesis, Advanced Functional Materials, n/a (2025) e09200.
